# Genetic mechanisms involved in the evolution of the cephalopod camera eye revealed by transcriptomic and developmental studies

**DOI:** 10.1186/1471-2148-11-180

**Published:** 2011-06-24

**Authors:** Masa-aki Yoshida, Atsushi Ogura

**Affiliations:** 1 Ochadai Academic Production, Ochanomizu University, Ohtsuka 2-1-1, Bunkyo, Tokyo, Japan

## Abstract

**Background:**

Coleoid cephalopods (squids and octopuses) have evolved a camera eye, the structure of which is very similar to that found in vertebrates and which is considered a classic example of convergent evolution. Other molluscs, however, possess mirror, pin-hole, or compound eyes, all of which differ from the camera eye in the degree of complexity of the eye structures and neurons participating in the visual circuit. Therefore, genes expressed in the cephalopod eye after divergence from the common molluscan ancestor could be involved in eye evolution through association with the acquisition of new structural components. To clarify the genetic mechanisms that contributed to the evolution of the cephalopod camera eye, we applied comprehensive transcriptomic analysis and conducted developmental validation of candidate genes involved in coleoid cephalopod eye evolution.

**Results:**

We compared gene expression in the eyes of 6 molluscan (3 cephalopod and 3 non-cephalopod) species and selected 5,707 genes as cephalopod camera eye-specific candidate genes on the basis of homology searches against 3 molluscan species without camera eyes. First, we confirmed the expression of these 5,707 genes in the cephalopod camera eye formation processes by developmental array analysis. Second, using molecular evolutionary (dN/dS) analysis to detect positive selection in the cephalopod lineage, we identified 156 of these genes in which functions appeared to have changed after the divergence of cephalopods from the molluscan ancestor and which contributed to structural and functional diversification. Third, we selected 1,571 genes, expressed in the camera eyes of both cephalopods and vertebrates, which could have independently acquired a function related to eye development at the expression level. Finally, as experimental validation, we identified three functionally novel cephalopod camera eye genes related to optic lobe formation in cephalopods by *in situ *hybridization analysis of embryonic pygmy squid.

**Conclusion:**

We identified 156 genes positively selected in the cephalopod lineage and 1,571 genes commonly found in the cephalopod and vertebrate camera eyes from the analysis of cephalopod camera eye specificity at the expression level. Experimental validation showed that the cephalopod camera eye-specific candidate genes include those expressed in the outer part of the optic lobes, which unique to coleoid cephalopods. The results of this study suggest that changes in gene expression and in the primary structure of proteins (through positive selection) from those in the common molluscan ancestor could have contributed, at least in part, to cephalopod camera eye acquisition.

## Background

Animal eyes have long been considered a classic example of convergent evolution. In recent decades, this view has changed due to the discovery of shared developmental regulatory genes for eye formation. Several genes, such as Pax-6/*eyeless *(*ey*) [1], *eyes absent *[2], *dachshund *[3], and *sine oculis *[4], together with their orthologs in metazoan animals, are able to induce the formation of ectopic eyes in flies and have been regarded as essential eye regulator genes among metazoan animals [5,6]. Most of the genes involved in eye development had already existed in the last common ancestors of cnidarians and bilaterians [7]. Such evidence suggests that some conserved genes have similarly contributed to eye development across a wide range of animals.

**Figure 1:**
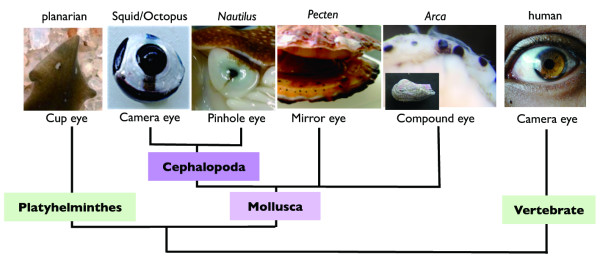
**Phylogenetic view of molluscan eye diversification**. Camera eyes were independently acquired in the coleoid cephalopod (squids and octopuses) and vertebrate lineages.

In contrast to the above discovery, the structural diversity of the eye is also evident among metazoan animals, and might have affected the diversification of species themselves by changing their morphology, behavior, and ecological strategy. The morphological unit of the eye has many different components such as muscle, lens, photoreceptor, optic nerve and visual center of brain, each with there own evolutionary histories [8,9]. Molluscs provide an appropriate model for the study of the evolutionary history of these various eye components as a number of different eye types are present in one phylum. In this study, we focused on the evolution of the camera eye in coleoid cephalopods (octopuses, cuttlefishes, and squids). There are two well-known cephalopod eye types; the pin-hole eye, found in nautiloids, and the camera eye, seen in coleoid cephalopods (Figure 1). Comparative studies on the camera eye of coleoid cephalopods and the pin-hole eye of nautiloids have begun to reveal the evolutionary histories of the various eye components and their genetic backgrounds. The coleoid cephalopods have an iris, a nearly circular lens, a vitreous cavity, and photoreceptor cells that form a retina. The nautiloid eye, however, consists only of a retina. These structural differences are the result of modifications that occurred after the divergence of cephalopods from the common molluscan ancestor [10]. Differences in visual cognition between the eye of the coleoid cephalopods and that of other molluscs appear to be a reflection of their complicated brain anatomy, as well as of their elaborated accessory eye structures [11]. The optic lobes of the coleoid cephalopods include secondary interneurons connected to photoreceptor cells, the cortex of which is arranged in four layers and resembles the organization of the deep layers of the vertebrate retina [12]. In contrast, lower molluscs, such as *Aplysia*, have tiny neuronal clusters between photoreceptors and the brain [13]. Therefore, the complicated cortex strucutre of the optic lobes of the coleiod cephalopods might be a new phenotype obtained in the coleoid cephalopod lineage.

The question remains as to what genetic background could have contributed to the evolution of the coleoid cephalopod camera eye. We hypothesized that changes in expresssion patterns and functions of pre-existing genes as well as the gain and loss of genes have played important roles in the evolution of the camera eye in coleoid cephalopods. Previously, Ogura et al. [14] revealed that more than 60% of octopus eye ESTs were commonly observed in the human eye, indicating that changes of expression patterns and functions have been involved in the evolution of the camera eyes in the octopus and humans.

**Figure 2:**
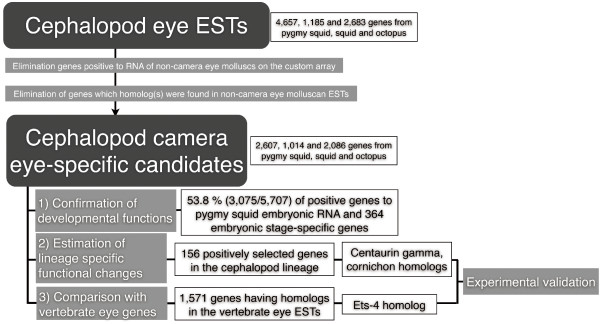
**Estimation and analysis procedures for cephalopod camera eye-specific candidate genes**.

To determine the genes specifically involved in camera-eye development and those for which functions may have changed during camera eye evolution, we applied the following three strategies (Figure 2). First, we utilized "*in vitro *homology search array technology" to remove genes commonly expressed across molluscan eyes and estimate genes specifically expressed in the coleoid cephalopod camera eye. This strategy was developed for the comparative genomic study of non-sequenced species with the aid of a bioinformatic approach to probe design [15]. In this analysis we used a 60 mer oligonucleotide library based on the expressed gene sequences of the octopus, squid, nautilus and scallop, in which nearly 10,000 annotated genes were examined. We then estimated candidate genes uniquely expressed in camera-type coleoid cephalopod eyes. Second, we compared gene expression patterns among three developmental stages in pygmy squid, which we used as a coleoid cephalopod model, to validate and elucidate gene expression in the camera eye formation process. It is known that the eye formation process can be observed during stages 20-25 in the pygmy squid, and we utilized embryos at stage 20, 25 and 30 for the developmental array. Third, to explore genes that have changed functionally, we determined whether the candidate genes in the coleoid cephalopods were under positive selection pressure. Finally, we selected four candidate genes, two transcription factor genes that might have changed the expression patterns of down-stream genes, and two positively selected genes that might have experienced functional changes. We then validated their localization at various developmental stages in our cephalopod model, the pygmy squid.

## Results

### Identifying genes specifically expressed in the coleoid cephalopod camera eye

To collect cephalopod camera eye-specific candidate genes, we performed an *in vitro *homology search using an array designed to estimate gene expression among species on the basis of inter-species hybridization signals. In this *in vitro *homology search array, probes were designed, using the probe design method described in Ogura et al. [15], to estimate distantly related homologs of molluscan eye genes. Microarray probes from *Octopus vulgaris *(octopus), *Loligo bleekeri *(squid), *Idiosepius paradoxus *(pygmy squid), *Mizuhopecten *(*Patinopecten*) *yessoensis *(scallop) and *Nautilus pompilius *(nautilus) were derived from our eye EST libraries and the NCBI EST database. We applied total RNA samples from the pygmy squid, scallop and nautilus to a custom Agilent microarray designed to detect specific gene expression in molluscan eyes. The microarray results for camera eye-specific genes allowed us to eliminate 173 and 162 genes found to be significantly expressed, using the Feature Extraction output (see methods), in the nautilus and scallop eye RNA, respectively. In addition to elimination on the basis of the microarray results, we also eliminated genes having homolog(s) among the nautilus or scallop eye ESTs, based on BLASTN and TBLASTX searches (<1e-4). As a result, a total of 5,707 genes (2,607, 1,014 and 2,086 genes from the pygmy squid, squid, and octopus, respectively) were selected as cephalopod camera eye-specific candidate genes (Figure 2). Although this array-based estimation may include false-positive genes (expressed in nautilus or scallop eyes but not detected in the array), as described in Gilad et al. [16], we expect highly conserved genes (common eye genes and housekeeping genes) to be eliminated using this analysis.

### Confirmation of cephalopod camera eye-specific genes expression in the developmental stages of the pygmy squid by developmental array

Next, we performed microarray experiments using pygmy squid embryonic RNA to estimate genes contributing to the camera eye developmental process in coleoid cephalopods. We extracted total RNAs from whole embryos at stage 20 and stage 30 (hatchlings), and from the eyes at stage 25. The reason for the selection of these three stages is that essential developmental events occur in each stage: stage 20 sees the establishment of eye placodes, stage 25 sees eye vesicle closure and the onset of lens formation, and stage 30 sees the completion of eye development (though genes used for the growth and maintenance of eyes are still active). Among the cephalopod camera eye-specific candidate genes selected above, 53.8% (3,075/5,707 genes) of the genes were found to have a positive signal against pygmy squid embryonic RNA.

**Figure 3:**
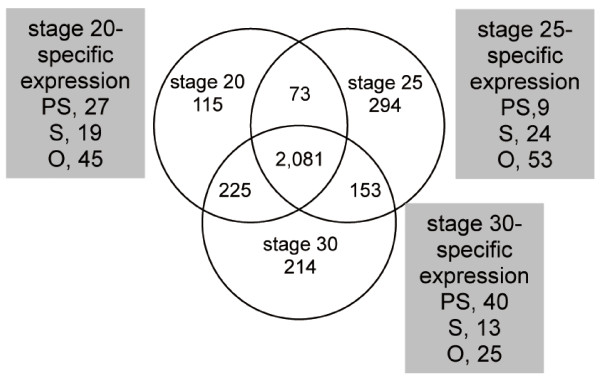
**Differential gene expression profiles of camera eye genes at different developmental stages**. The Venn diagram indicates numbers of cephalopod camera eye-specific genes for three cephalopod species (PS, the pygmy squid; S, squid; O, octopus). Each box represent the number of genes expressed at the three embryonic stages of the pygmy squid found in the developmental array. In total, 3,075 genes (2,320, 235 and 519 from PS, S, and O, respectively) were positive against pygmy squid embryonic RNA.

We then checked positive gene expression at the three developmental stages, as the highly expressed genes are expected to be specifically involved in the developmental events occurring at each stage. We isolated 115 genes positive against only embryonic RNA at stage 20. Similarly, 294 and 214 genes were isolated at stage 25 and stage 30, respectively (Figure 3). After eliminating genes expressed in the adult eye of the pygmy squid, 6.38% (141, 73, 150 from the pygmy squid, squid, and octopus, respectively) of the genes were selected as showing high expression at these embryonic stages.

### Estimation of lineage-specific functional changes in coleoid cephalopod camera eye-specific genes by molecular evolutionary analysis

We hypothesized that taxon-specific gene modification contributed to the evolutionary steps in coleoid cephalopod camera eye development. To clarify the involvement of gene recruitment and subsequent positive selection in camera eye acquisition, we estimated the dN/dS ratio of camera eye-specific candidate genes after the divergence of cephalopods from the common molluscan ancestor. The cephalopod camera eye-specific data were concatenated together with homologous genes estimated by BLASTN and TBLASTX searches against the molluscan NCBI nucleotide collection and molluscan ESTs (an e-value of less than 1e-20). Phylogenetic trees based on the gene sets were analyzed using the Codeml program in PAML ver. 4.4 b [17] under the F3x4 model. Finally, we obtained 1,391 sets of multiple alignments including cephalopods and other molluscs, and dN/dS values of the camera eye-specific genes. Of the camera eye-specific genes, 156 (68, 43, 24 from the pygmy squid, octopus, and squid, respectively) were identified as positively selected genes in the cephalopod lineage (the dN/dS ratio was significantly higher than 2, Additional file 1, Table S1).

Homologs of the cornichon gene were found among the octopus and the pygmy squid ESTs (5primeCluster0328, DB919298), and both homologs were found to be positively selected genes in the cephalopod lineage. Cornichon-like genes in vertebrates have been shown to be located directly downstream of Pax-6 [18]. It is of great interest that the region downstream of Pax-6 is included among the camera eye-specific genes, although it is still unclear whether the squid cornichon is controlled by Pax-6 or not.

Centaurin gamma is a small GTP family member with NTPase activity [19] (Additional file 2, Figure S1, Additional file 3, Supplementary data S1). It is known to be expressed in human cancer cells is known; however, its function in vertebrate development is unclear. Localization of the centaurin gamma homolog (OctEye_3919F_082) is described below.

### Determination of common and unique genes by comparative gene expression analysis of cephalopods and vertebrates

We next investigated whether coleoid cephalopod camera eye-specific genes could be found in the vertebrate camera eye by comparing gene expression profiles across a wide range of animal phyla. A comparison of the coleoid cephalopod eye-specific candidate genes showed that 1,571 have an e-value of less than 1e-04 in BLASTN and TBLASTX searches against vertebrate eye EST data including the National Eye Institute's NEI Bank, NCBI UniGene, and Bodymap data (Figure 2, Additional file 1, Table S1). We found that 56.2% (839, 16, 28 genes from the pygmy squid, squid and octopus, respectively) of these probes have a positive signal against pygmy squid adult eye RNA. This indicates that these candidates include genes commonly expressed in cephalopod species, and that these genes are also involved in coleoid cephalopod camera eye. The list of homologous genes indicates that 63.7% of the genes (578, 38, 384 genes from the pygmy squid, squid and octopus, respectively) are function-known in vertebrate genome annotation and GO classification analysis. We then examined the biological functions of the camera eye-specific genes using single enrichment analysis against total cephalopod ESTs except the coleoid cephalopod camera eye-specific genes (those with an e-value of less than 0.01, Additional file 4, Figure S2). The GO classification analysis showed that fifteen terms associated with "Molecular Function" were significantly over-represented in the camera eye-specific genes, including genes for protein binding, nucleotide binding, structural constituent of ribosomes, ATP binding, translation initiation factor activity, GTP binding, zinc ion binding, metal ion binding, catalytic activity, binding, protein homodimerization activity, and transferase activity (single enrichment analysis using the blast2go, an P < 0.01, Table 1). Terms of "Biological Process" category were also found to be an over-represented, including translation, intracellular protein transport, signal transduction, small GTPase mediated signal transduction, protein transport, protein modification process, and anti-apoptosis. On the other hand, no particular term of "Cellular Component" was over-reperesented in the camera eye-specific genes. These over-represented GO terms shown above also represent many Ras-like signal transduction proteins and zinc finger proteins in the camera eye-specific gene set.

**Table 1:** Over-represented GO terms in the camera eye-specific genes.

GO term	GO name*	P-Value
GO:0005515	protein binding (F)	9.76E-43
GO:0000166	nucleotide binding (F)	1.63E-06
GO:0003735	structural constituent of ribosome (F)	3.17E-06
GO:0006412	translation (P)	7.30E-05
GO:0006886	intracellular protein transport (P)	5.08E-10
GO:0005524	ATP binding (F)	4.04E-21
GO:0003743	translation initiation factor activity (F)	1.09E-04
GO:0005525	GTP binding (F)	6.87E-08
GO:0007165	signal transduction (P)	9.48E-06
GO:0008270	zinc ion binding (F)	1.20E-08
GO:0007264	small GTPase mediated signal transduction (P)	1.15E-04
GO:0015031	protein transport (P)	5.43E-07
GO:0046872	metal ion binding (F)	3.38E-05
GO:0003824	catalytic activity (F)	1.18E-05
GO:0005488	binding (F)	1.57E-07
GO:0042803	protein homodimerization activity (F)	6.54E-05
GO:0006464	protein modification process (P)	6.67E-05
GO:0006916	anti-apoptosis (P)	1.27E-06
GO:0016740	transferase activity (F)	1.07E-05

* (F), GO terms Molecular Function; (P), Biological Process.

**Figure 4:**
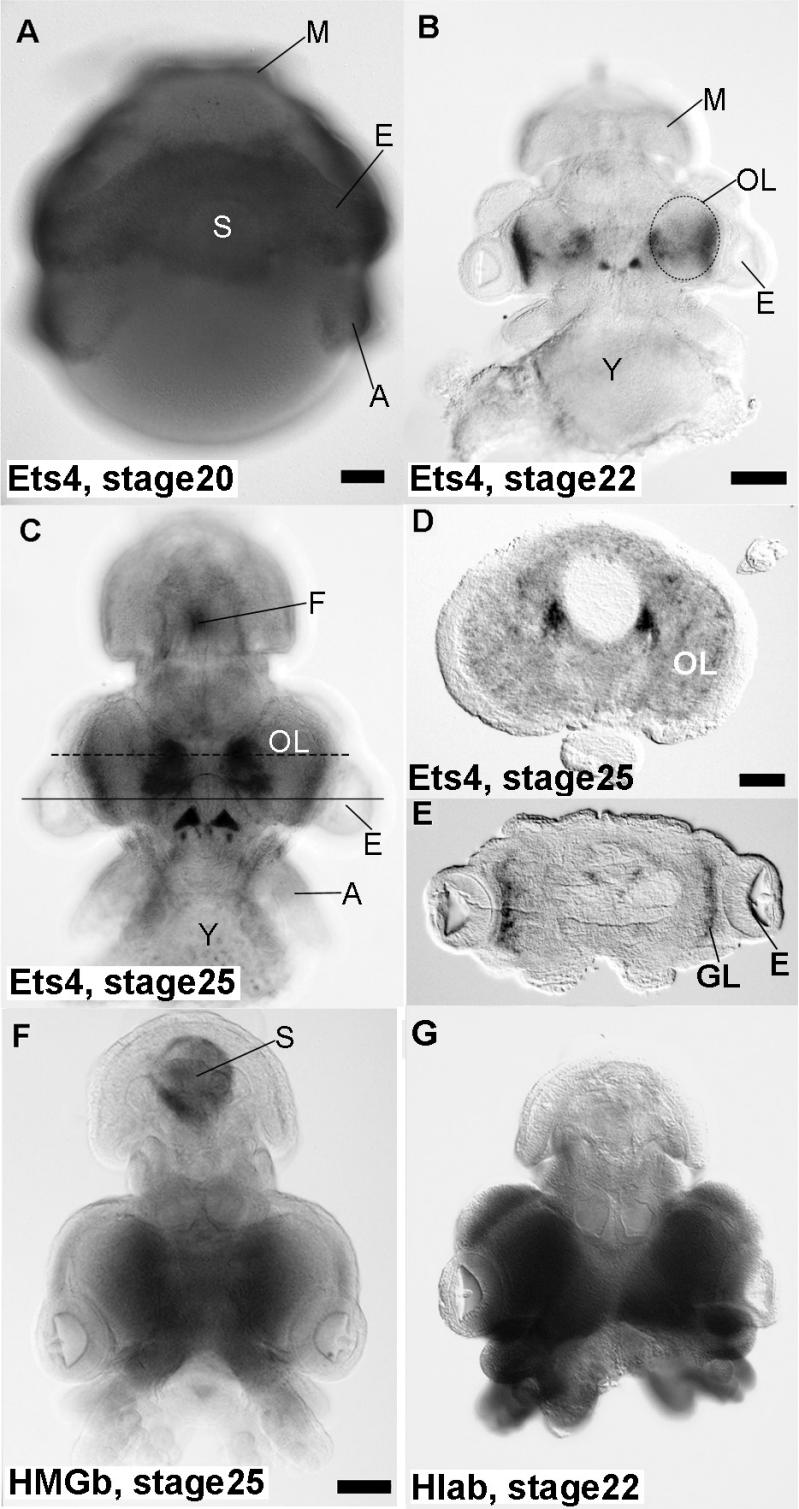
**Localization of camera eye-specific genes in the pygmy squid embryos**. (A-C) Whole-mount *in situ *hybridization with probes for the Ets-4 homolog. (D, E) Whole-mount *in situ *hybridization with probes for the Hla-b associated transcript homolog. (F) Whole-mount *in situ *hybridization with probes for the HMGb3 homolog. (A) Tissue surrounding the eye primodia (E) and tips of the arms (A) at stage 20 expressed the Ets-4 homolog. The Ets-4 transcripts were not detected in the mantle (M). (B) The Ets-4 transcript localized in the external part of the optic lobes (OL) and the central part of brain at stage 22. The yolk sac (Y) was removed using forceps. (C) Specific staining was localized in the optic lobes and central part of the brain at stage 25. The Ets-4 homolog was also expressed in the funnel organ (F). (D) A horizontal cryosection at the dotted line in (C). The Ets-4 transcripts appeared to be localozed in the part of the brain (the middle esophageal mass). Up side showes dorsal of the body. (E) A horizontal cryosection at the solid line in (C). The Ets-4 transcripts apper to be restricted in glanular cell layer (GL) in the optic lobes. (F) The HMGb3 transcripts were restricted to the internal part of the optic lobes at stage 25. Non-specific staining was found in the shell sac (S), as shown in the control experiment (Figure 5c). (G) The specific staining of the Hla-b-associated transcript was found in the head region, including the optic lobes, at stage 22. (A), stage 20. (B), (G), stage 22. (C), (D), (E), (F), stage 25. Bar = 100 μm in (A)-(C), (F), (G); 50 μm in (D), (E).

To estimate the genes possibly related to camera eye specification through subsequent structural and functional gene expression, we further manually identified several transcriptional factors using the GO classification and domain estimation results obtained by the Pfam. Homologs of six homeobox 2 (00672_Oc_096), ets-related isoform 4 (DB913089), lim domain-containing (06182_Oc_5_043), lim and sh3 domain protein (OctEye_4576F_074) and high mobility group b3b (01791_Oc_5_056) were found among the camera eye-specific genes. There were also many zinc finger homologs (02158_Oc_5_047, 06176_Oc_5_032, 5primeCluster0192, OctRet_1427F_085, DB912036, DB912793, DB914855, DB918248 and DB918617). Molecular phylogenetic analysis revealed that the squid Ets-4 homolog was closely related to the vertebrate Erg and Fli-1 genes (Additional file 5, Figure S3, Additional file 6, Supplementary data S2), which are major regulators of blood vessel development [20]. The squid Ets-4 homolog is thought not to have any putative function in the squid blood vessels due to its expression pattern (described below, Figure 4), although many small vessels supply blood to the cephalopod retina [21]. When we applied an *in vitro *homology search array technique to a comparison of the pygmy squid and human transcripts [15], a probe based on the Ets-4 homolog reproducibly cross-reacted with human RNA (unpublished data). These results indicate not only that our array could effectively identify similar expression patterns across animal phyla, but also that the expression of the Ets-4 homolog was restricted to the camera eye of vertebrates and cephalopods.

### Functional analysis of four candidate genes selected among the coleoid cephalopod camera eye-expressed genes

To clarify the function of the coleoid cephalopod camera eye-specific candidate genes in the camera eye formation process, we cloned and analyzed the localization of transcripts of three candidate genes in the pygmy squid embryo. We selected the Ets-4, HMGb3 and Hla-b associated transcript homologs from the transcription factor genes commonly expressed in vertebrates and cephalopods due to their high expression signals and the existence of human orthologs for further functional analysis (Additional file 1, Table S1). The centaurin gamma homolog was also selected from the positively selected gene candidate genes in the cephalopod lineage as it showed a high dN/dS ratio and had a human ortholog (Additional file 1, Table S1, Additional file 2, Figure S1, Additional file 3, Supplementary data S1).

**Figure 5:**
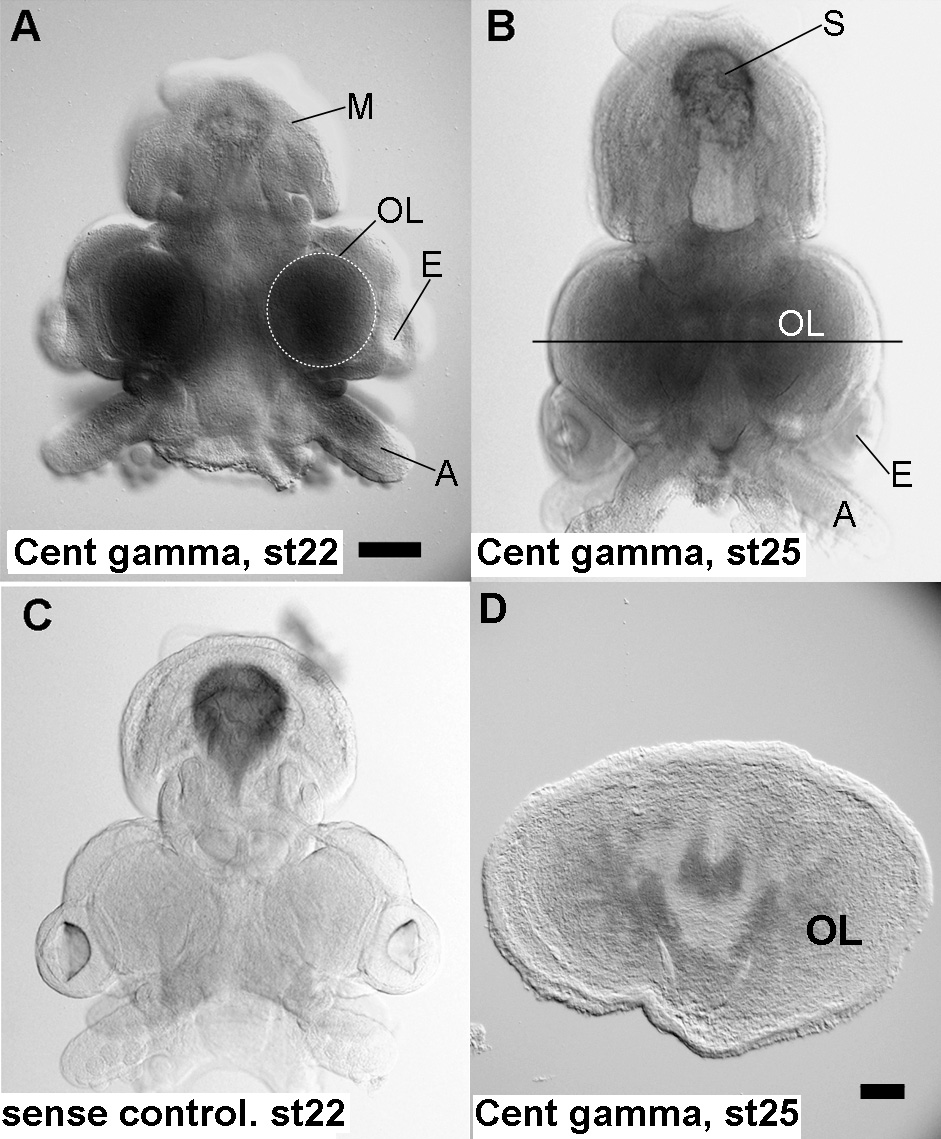
**Localization of positively selected genes within the cephalopod camera eye**. (A, B) Whole-mount *in situ *hybridization with probes for the centaurin gamma homolog. (C) Control experiment using whole-mount *in situ *hybridization with sense probes for the centaurin gamma homolog. (A) The centaurin gamma transcripts were detected in the optic lobes (OL), but not in the mantle (M) or eyes (E) at stage 22. (B) Specific staining was found in the head region, including the optic lobes (OL), at stage 25. Non-specific staining was detected in the shell sac (S), as shown in (C). (C) Non-specific staining was detected in the shell sac, but not in the head region. (D) A horizontal cryosection at the solid line in (B). The Centaurin gamma transcripts appears to be restricted in the perikaryal part of the central brain and optic lobes (OL). (A), (C), stage 22. (B), stage 25. Bar = 100 μm in (A-C); 50 μm in (D).

Among the above-mentioned genes common to both cephalopods and vertebrates, the Ets-4 homolog showed positive expression against embryonic RNA. We validated its expression in the three developmental stages (20, 25 and 30) as well as its localization by *in situ *hybridization using pygmy squid embryos. Ets-4 transcripts were localized in the surface of the optic lobe and in part of the brain (Figure 4a-c). The transcripts appeared in tissues surrounding the future eye and the tips of the arm anlagens of the embryo at the stage 20 (Figure 4a). In terms of brain development, primodial brain lobes appears before stage 20 (even in stage 18) [22]. Optic lobes become visible on the inner side of the eye vesicles at stage 21. The retinal primodium is distinguishable by rectangular cells at stage 18, and retinal pigmentation starts at stage 20. Embryos at stage 25 show a dark brown retina. The Ets-4 expression then appear to be restricted to the part of middle subesophageal mass (Figure 4d), the external part of the optic lobes (Figure 4e) and paired structure aside the buccal mass at stage 22 (Figure 4b) and stage 25 (Figure 4c) in comparison to the brain atlass [23]. Strong siganals were observed in the most external layer, which correspond to outer granular layer of the optic lobes (Figure 4e). This expression pattern is possibly related to the visual cognition system of the squid as the granular layer of the optic lobes are directly connected to the photoreceptors and act as secondary neurons of the visual circuit. HMGb3 transcripts appeared in the body of the stage 20 embryo including the eye field, mantle, and the tip of the arm anlagens (data not shown), but were restricted to the inner part of the optic lobes at stage 25 (Figure 4f). Hla-b associated transcript-specific staining was observed in the head region, including the optic lobes, of the stage 22 embryo (Figure 4g). Localization of the centaurin gamma homolog from positively selected genes within the squid embryo was also analyzed using an *in situ *hybridization assay (Figure 5a, b). Transcripts were continuously distributed in the optic lobes (Figure 5d), and this expression pattern suggests that the centaurin gamma homolog is related to developmental or proliferative steps in the optic lobe neurons.

## Discussion

### Genetic mechanisms involved in camera eye evolution of the coleoid cephalopods

The coleoid cephalopod camera eye-specific candidate genes selected by our analysis might be divided into two groups. The first group consists of genes that commonly exist in animals, but whose expression level has been changed through the structural changes of the eyes after the divergence of the cephalopods. The Ets-4 homolog, for example, was expressed in the granular cell layers specifically found in the optic lobes in coleoid cephalopods. The fact that a large proportion of the genes expressed in the octopus eye was found in the genomes of other animals [14] supports the notion that the role of the genes might have changed in relation to the structural changes in the various eye types. The second group comprises genes that commonly exist, even in molluscan eyes, but whose functions have been changed by positive selection in relation to camera eye development in cephalopods. We identified more than a hundred genes that appear to have undergone positive selection (dN/dS values of more than 2) in the coleoid cephalopod lineage. The genetic mechanism represented by these two groups seems to have been contributed to acquisition of the camera eye.

Because the *in vitro *homology search array could be used to detect highly expressed genes in humans and squids [15], we thought it possible to remove highly expressed molluscan eye genes, including housekeeping genes, from the list of candidate genes to estimate cephalopod specificity. The cephalopod camera eye-specific candidate gene set also contain genes common among animals with eyes, all of which should, ideally, be removed along with the nautilus and scallop gene pool but which nevertheless remained due to the use of incomplete gene sets.

### Functions of the coleoid cephalopod camera eye-specific genes found in this study

One of the most intriguing events in coleoid cephalopod camera eye evolution is the transition from the simple eye to a more complex visual eye. The transition was accompanied by the evolution of the eye components including the optic lobes, visual center of the brain. In this transition, expression changes and functional chagnges of the camera eye specific candidate genes might have been contributed to the acquisition of the eye compnents. For example, Ets-4 localization in the five-layered optic lobes of the pygmy squid appears to be involved in the organization of outer granular layer. As Erg and Fli-1, orthologs of Ets-4 in vertebretes, are major inducers of blood vessel development [20] and D-ets-3 and D-ets-6, orthologs of Ets-4 in *Drosophila*, are known to be expressed in the ventral nerve cord [24,25], it is speculated that Ets-4 of the pygmy squid was recruited for the development of different organs, particularly in the conversion of neuron responses in cephalopod optic lobes.

The cornichon and centaurin gamma homologs identified as camera eye specific genes in cephalopods are also known to be expressed in the vertebrate camera eye. Even though these transduction-related genes are completely function-unknown, together with their upstream and downstream, their observed localization in the optic lobe suggests their involvement in the neuronal development of the coleoid cephalopod camera eye. The HMGb3 and Hla-b associated transcript homologs may be involved in visual circuit formation through chromatin remodeling and initiation of subsequent gene expressions. As a consequence of our study, a number of common genes from the common molluscan ancestor have been shown to be involved in coleoid cephalopod camera eye evolution through changes in their expression patterns and functions.

## Conclusion

In this study, we selected 5,707 genes as coleoid cephalopod camera eye-specific candidates by homology searches and comparative expression against the nautilus and scallop eyes. We applied a molecular evolutionary approach to the cephalopod camera eye candidates to identify genes that had functionally changed after the divergence of cephalopods from the common ancestor. Our developmental approach, which included developmental arrays and *in situ *hybridization using the pygmy squid embryos, indicated the contribution of camera eye-specific genes to the eye developmental process. In particular, some camera eye specific genes, such as the Ets-4, cornichon, and centaurin gamma homologs, was shown to have contributed to camera eye evolution through the development of a complex visual circuit.

## Methods

### Pygmy squid cultures and RNA isolation

Japanese pygmy squid, *Idiosepius paradoxus*, specimens were obtained from Chita Peninsula, Nagoya, Japan and kept in our laboratory, as previously described [26], where the embryos were staged [22]. The embryonic eyes of stage 25 embryos were dissected from the body using forceps and used for RNA extraction. A specimen of *Nautilus pompilius *was purchased from a retail aquarium in Kanagawa, Japan. A specimen of the Japanese scallop, *Mizuhopecten (Patinopecten) yessoensis*, was purchased from the Tokyo Central Wholesale Fish Market (caught in Aomori). Total RNA was extracted from the embryos or adult tissues using an E. Z. N. A. Mollusc RNA kit (Omega Bio-Tek, Inc.), according to the manufacturer's protocol.

### Probe design for the *in vitro *homology search of the molluscan eyes

Molluscan EST data for *Loligo bleekeri *(squid), *Mizuhopecten (Patinopecten) yessoensis *(scallop) and *Nautilus pompilius *(nautilus) were obtained from cDNA libraries constructed using the Creator™ SMART™ Library Construction Kit (Takara Clontech) with the TRIMMER-DIRECT cDNA normalization kit (Evrogen) and introduced into DH5α electro competent cells (Takara). The 5' regions of the cDNA libraries were sequenced using the M13 forward primer (5-TGT AAA ACG ACG GCC AGT-3) and Big Dye Terminator v3.1 (Applied Biosystems) on an ABI3030 sequencer (Applied Biosystems). Raw data for these ESTs are available under the following accession numbers: [DDBJ:FY298839-FY302524]. The sequence clean-up program Lucy (The Institute for Genomic Research, Rockville, MD; http://www.tigr.org/softlab) was used to trim vector sequences and low-quality sequences from the raw data. These ESTs were assembled using the MIRA assembler 3.2.1 [27], and then used for the subsequent analyses. The octopus ESTs were obtained as previously described [14] and from the following website http://www.cib.nig.ac.jp/dda/database/octopus.htm. We adopted ESTs of *I. paradoxus *from the NCBI EST database under the following accession numbers: [DDBJ, DB910977-DB920055]. Redundant sequences in the *I. paradoxus *ESTs were found using BLASTCLUST and removed. These data were concatenated and used as molluscan eye ESTs in the subsequent analyses, and are available upon request. We added the pubmed data sets for vertebrate eye genes, obtained from a public database, to identify eye gene expression across distantly related species. Finally, we obtained 12,128 molluscan probes (4,657, 1,483, 3,377, 1,493 and 1,118 from the pygmy squid, squid, octopus, scallop and nautilus, respectively) and designed a custom microarray with the Agilent 8 × 15K format custom microarray service.

### Developmental array

To perform the comparative analysis of various developmental stages, we applied total RNA samples from the molluscan species onto the custom microarray. Total RNA from the embryonic stages were labeled and applied separately. Briefly, 0.5 μg of total RNA from each sample was used to synthesize fluorescent-labelled cRNA using Cyanine 3-CTP according to the manufacturer's protocol (Agilent Quick Amp Labeling Kit, one-color). Labeled DNA was hybridized for 16 hr at 65°C on the custom array. After hybridization, the microarray slides were washed according to the standard protocol (Agilent Technologies) and scanned on an Agilent microarray scanner. Data were analyzed using the Agilent Feature Extraction Software (v10.7, Additional file 7, Table S2). We adopted the microarray data under the following accession number [CIBEX, CBX187]. We selected wells (spots) exhibited a signal "well above the background" and "positive and siginificant" as siginificantly expressed genes using the Feature Extraction output.

### Databases for homology searches among molluscs and vertebrates

Molluscan eye ESTs were obtained as described above. For comparison with the vertebrate eye EST data, we used data from Bodymap, UniGene, and NEI Bank (Accession numbers are listed as Additional file 8, Supplement data S3, Additional file 9, Supplement data S4, Additional file 10, Supplement data S5, respectively). For the comparative analysis of gene expression, we obtained human-eye ESTs (929 contigs) of the retina, corneal endothelium, cornea, and iris from BodyMap [28]. NEI Bank, maintained by the National Eye Institute, a division of the NIH (USA), contains data (680,045 contigs) for several human-eye cDNA libraries [29], including tissue-separated databases of the ciliary body, cornea, fovea, iris, lens, optic nerve, retina, RPE choroids, trabecular meshwork, and other tissues/organs. We also used data sets from the vertebrate lens, retinal and eye libraries (484, 202 contigs from humans, mice, rats, rabbits, dogs, pigs, sticklebacks, zebrafish, salmon, and pufferfish) in the NCBI UniGene [30].

### Functional annotation

Functional annotation was conducted as follows. Gene ontology, which is defined by the Gene Ontology Consortium [31,32], and used to categorize genes by (1) Molecular Function, (2) Biological Process, or (3) Cellular Component, was used to categorize cephalopod eye ESTs for the octopus, squid and pygmy squid. We adopted all GO terms for categorizing cephalopod eye genes using a blast2GO [33]. Enrichment analysis of the camera eye-specific genes against total cephalopod ESTs excluding the camera eye-specific genes, was carried out with the blast2GO program (using Fisher's exact test, an e-value of less than 0.01, Table 1). The Pfam database [34] was used to estimate the domain structure of the molluscan eye ESTs. Domains in the translated amino acid sequences of the molluscan eye ESTs were estimated using batch search from the Pfam website.

We determined transcriptional factors in the cephalopod camera eye-specific candidate genes manually using the sequence description obtained by the blast2GO, and the HMM-name obtained by the Pfam programs. Homologs of six homeobox 2 (00672_Oc_096), ets-related isoform 4 (DB913089), lim domain-containing (06182_Oc_5_043), lim and sh3 domain protein (OctEye_4576F_074) and high mobility group b3b (01791_Oc_5_056) were identified as they have homeobox, ETS, LIM, LIM and high mobility group domains. We also identified genes involved in signal transduction cascades, homologs of rab5 protein (02945_Oc_5_065), ras-related protein rab-2 (DB916866), member ras oncogene family (ika1224-No19_E06_001, DB913730 and DB919285), ran gtpase-activating protein (DB912649), and dishevelled associated activator of morphogenesis 1 like (03249_Oc_5_081), which are possibly related to camera eye specification. Indeed, the Ras superfamily of small GTPases regulate many cellular regulatory and developmental pathways, including diurnal regulation of the octopus photoreceptors [35]. As previously reported [14], retinal arrestin (08322_Oc_5_073), retinal dehydrogenase (DB913426), neuron-specific enolase (5primeCluster0558), and gelsolin (07345_Oc_5_049, 08462_Oc_5_015) are commonly expressed and are thought to be involved in camera eye formation.

### Molecular evolutionary (dN/dS) analysis

We calculated the dN/dS ratio in the cephalopod lineage (squids or octopus) after divergence from other molluscs to estimate genes changed through positive selection in the cephalopod camera eye. The nucleotide sequences and deduced amino-acid sequences of the cephalopod eye cDNAs isolated in the present study were aligned together with estimated homologs of other molluscs using homology searches. The homologs were selected using BLASTN and TBLASTX against the non-cephalopod molluscan eye ESTs (scallop and nautilus) and the molluscan gene sets obtained from the NCBI nucleotide and EST collections (Taxonomy ID: 6447). Homology searches of the GenBank non-redundant database using BLAST with a cutoff value of < 1e-20 produced matches against 1,391 of the contigs. The homologs were concatenated and aligned using the Clustal W program (default options) [36]. After removing gap positions, the dN/dS ratio (Kn divergence per Ks divergence) between each homologous gene set was estimated using the maximum likelihood method of Goldman and Yang [37], as implemented in the codeml program of the PAML package [17] under the F3 × 4 model. The statistical significance of positively selected genes was determined using a one-tailed student's *t*-test. The above-mentioned protocol was systematically performed using both Perl and shell scripts.

### Targeted-gene cloning in the pygmy squid and expression analysis

We selected the cephalopod camera eye candidate genes using the above-mentioned criteria. Orthologous candidates in the pygmy squid were then cloned to validate their expression pattern. Fragments of the Ets-4, HMGb3, Hla-b associated transcript, cornichon and centaurin gamma homologs were obtained by PCR reaction using pygmy squid eye cDNA. Reverse transcription was carried out using a PrimeScript™ RT Reagent kit (TaKaRa), and the primers used to amplify the target genes are listed in Additional file 11, Table S3.

Longer length cDNAs of the Ets-4 and centaurin gamma homologs were obtained using a BD SMARTer RACE (rapid amplification of cDNA ends) cDNA amplification kit (Clontech). Ready-to-use first strand cDNA was synthesized according to the manufacturer's protocol using pygmy squid embryonic eye RNA. Total RNA was prepared from a number of pygmy squid hatchling specimens using an RNeasy mini RNA extraction kit (QIAGEN). The primers used to amplify the cDNA extremities were Ets4-5RACE, AAGTTGTATCACCTGGGAGGGCCG, Ets4-3RACE, CTTTTTGCAGCGCCGGTTCCTATT, centaurin gamma-5RACE, ACGCGAGGATTGCTTTCACTGATGG, and centaurin gamma-3RACE, GGTCCGCCGGAGATGCAGTTTACTC. The RACE reaction products were cloned into a plasmid using a pGEM T-vector system (Promega). Plasmid DNA from transformant colonies was purified with a GenElute™ Plasmid Miniprep kit (Sigma-Aldrich). Both strands of the plasmid DNA were fully sequenced downstream of an insert site by the dideoxy chain-termination method using a BigDye^® ^Terminator v. 3.1. The sequences are available under the following accession numbers: Ets-4 homolog, AB586703; centaurin gamma homolog, AB586704.

Short fragments of these genes were amplified by PCR and subcloned into the pGEM T-vector. These were then used as a template to generate digoxigenin (DIG)-labeled antisense and sense probes by *in vitro *transcription with a DIG-RNA labeling kit (Roche) using SP6 RNA polymerase (Roche) and T7 RNA polymerase (Roche). Whole-mount *in situ *hybridization for stage 20, 22, and 25 of the pygmy squid embryos was carried out using established procedures [26].

## Authors' contributions

MY performed mRNA extraction, *in situ *hybridization analyses and data analysis. AO conceived of the study, participated in its design and performed the statistical analysis. MY and AO conducted the microarray experiments. Both authors read and approved the final manuscript.

## Supplementary Material

Additional file 1 **TableS1**. A list of all the camera eye-specific genes commonly expressed in cephalopods and vertebrates. Click here for file

Additional file 2 **FigureS1**. Phylogenetic tree based on the Ras domain sequences of centaurin superfamily members. Click here for file

Additional file 3 **Supplementary_data_S1_ Cent_Rasdomain.fasta**. A fasta file based on phylogenetic analysis in Figure S1. Click here for file

Additional file 4 **FigureS2**. Distribution in camera eye-specific genes of GO terms at level 2. Click here for file

Additional file 5 **FigureS3**. Phylogenetic tree based on Ets domain sequences of Ets transcription factor superfamily members. Click here for file

Additional file 6 **Supplementary_data_S2_ Ets_domain.fasta**. A fasta file based on phylogenetic analysis in Figure S3. Click here for file

Additional file 7 **TableS2**. Numbers of microarray probes showing positive signals to the molluscan RNAs. Click here for file

Additional file 8 **Supplementary data S3_NEIB_ID_list**. A list of accession numbers obtained from the NEI bank. Click here for file

Additional file 9 **Supplementary_data_S4_Unigene_ID.list**. A list of accession numbers obtained from the Unigene database. Click here for file

Additional file 10 **Supplementary_data_S5_Bodymap_ID.list**. A list of accession numbers obtained from the Bodymap database. Click here for file

Additional file 11 **TableS3**. Primers to amplify homologs in the pygmy squid. Click here for file
