# Supplementary material for: Genetic mechanisms involved in the evolution of the cephalopod camera eye revealed by transcriptomic and developmental studies
Source: BMC Evol Biol. 2011 Jun 24;11:180. doi: 10.1186/1471-2148-11-180 (PMC3141435; doi:10.1186/1471-2148-11-180)
Supplement: Additional file 1 — TableS1. A list of all the camera eye-specific genes commonly expressed in cephalopods and vertebrates. [file 1471-2148-11-180-S1.PDF]

Table S1. List of camera eye-specific genes commonly expressed in cephalopods and vertebrates

sid	Annotations	best_hit by BLAST searches against vertebrate eye ESTs	e_value	bit_score	GO annotation	GO_id	domain	dN/dS mean
00016_Oc_016		gi|19004791|gb|BM691533.1|BM691533	8.00E-05	41.8	mediator of rna polymerase iisubunit 28			
00048_Oc_048		gi|62889664|gb|DN904901.1|DN904901	0	174	inositol-1(or 4)-monophosphatase 1	F:GO:0005488		
00058_Oc_061		gi|62854000|gb|DN884045.1|DN884045	0.004	44.1				
00123_Oc_022		gi|75848117|gb|DT878377.1|DT878377	0	222	comm domain containing 2	P:GO:0008150,C:GO:0005575		0.11667
00164_Oc_074		BY795245	6.00E-40	129	nadh dehydrogenase1 beta19kda	C:GO:0044464		1.0398
00258_Oc_073		gi|75832801|gb|DT863061.1|DT863061	3.00E-09	41.4	mitochondrial ribosomal protein l32-like protein			0.82538
00342_Oc_059	**Embryo specific expression**	gi|62850164|gb|DN880209.1|DN880209	0	202	p300 cbp-associated		Acetyltransf_1	
00348_Oc_062		gi|13288556|gb|BG395108.1|BG395108	0	250	vacuolar atp synthase subunit e			0.11737
00431_Oc_040		gi|37636484|gb|CF740146.1|CF740146	1.00E-06	56			DSS1_SEM1	
00547_Oc_066		gi|41338221|gb|CK617335.1|CK617335	0	210	fumarate hydratase 1	F:GO:0043565,F:GO:0003700,C:GO:0005634,P:GO:0006355	Lyase_1	
00659_Oc_082		gi|22810228|gb|BU503995.1|BU503995	0.001	28.1				
00672_Oc_096	**GO annotation**	gi|85198094|gb|DW604467.1|DW604467	0	262	six homeobox 2	F:GO:0043565,F:GO:0003700,C:GO:0005634,P:GO:0006355	Homeobox	

00697_Oc_021		gi|54449080|gb|CV560442.1|CV560442	9.00E-33	140	26s proteasome non-atpase regulatory subunit 13	C:GO:0044424,C:GO:0043234		0.8005
00722_Oc_057		gi|49671858|gb|CO425564.1|CO425564	2.00E-29	87.7	tho complex 5	F:GO:0005515,P:GO:0045650	FimP	
00738_Oc_073		gi|24730764|gb|CA395363.1|CA395363	0	133	26s protease regulatory subunit 6a		Pfam-B_6879, DUF3713	0.80709
00760_Oc_092		gi|58035732|gb|CX733258.1|CX733258	7.00E-35	147	transmembrane protein 66	C:GO:0016021	DUF1183	1.10953
00806_Oc_5_043		gi|77503943|gb|DV215357.1|DV215357	6.00E-10	64.9	cg050 protein		DUF2373	
00820_Oc_5_058		gi|37628497|gb|CF732167.1|CF732167	4.00E-09	51.5	nsfl1 cofactor p47			
00824_Oc_5_060		gi|22681745|gb|BU167761.1|BU167761	0.002	41.4	ligand-independent activating molecule for estrogen receptor	F:GO:0004872		
00913_Oc_5_049		gi|32174379|gb|CD672648.1|CD672648	7.00E-23	106	mitochondrial ribosomal protein s7		Ribosomal_S7	
01019_Oc_5_054		gi|46813061|gb|CN500437.1|CN500437	1.00E-25	115	diazepam binding inhibitor	F:GO:0008289	ACBP	1.18378
01047_Oc_5_084		gi|19116539|gb|BM799716.1|BM799716	0.001	46.1				
01239_Oc_5_084		gi|46465385|gb|CN459659.1|CN459659	0.001	41.4				
01265_Oc_5_017		gi|62847240|gb|DN877285.1|DN877285	3.00E-08	57.4	novel proteinfamily with sequence similaritymember a		DUF676	
01271_Oc_5_020	**Positive selection**	gi|62046375|gb|DN691721.1|DN691721	5.61E-45	180	nadp transhydrogenase		PNTB	2.8457

01284_Oc_5_042		BY795228	5.00E-06	44.6	mt-ezrin radixin moesin		ERM	
01304_Oc_5_060	Positive selection	gi|40322928|gb|CK356996.1|CK356996	1.00E-27	122	methylosome protein 50			2.65794
01319_Oc_5_068		gi|14062519|gb|BG751866.1|BG751866	2.00E-06	52.4	novel proteinvertebrate diacylglycerolbeta 90kda			
01349_Oc_5_003		gi|62884021|gb|DN899258.1|DN899258	0.0003	44.1	low-density lipoprotein receptor-related protein	P:GO:0007160,F:GO:0005509,C:GO:0045178,P:GO:0040010	Ldl_recept_b	
01372_Oc_5_030		gi|32180675|gb|CD678944.1|CD678944	2.00E-23	75.8	accessory gland protein			
01421_Oc_5_071		gi|54448708|gb|CV560260.1|CV560260	0.002	42.8				
01489_Oc_5_049		gi|5033517|gb|AI716264.1|AI716264	3.00E-06	49.2	dipeptidylpeptidase 7			
01490_Oc_5_057		gi|12040989|gb|BF725078.1|BF725078	2.00E-12	72.4	non-muscle myosin ii heavy chain	F:GO:0005524,F:GO:0003779,F:GO:0000166,F:GO:0003774,C:GO:0016459		
01494_Oc_5_059		gi|41338320|gb|CK617434.1|CK617434	2.00E-24	111	kelch domain-containing protein 8a		Kelch_1	0.90463
01510_Oc_5_075		gi|19029697|gb|BM716439.1|BM716439	1.00E-10	48.3	x-prolyl aminopeptidase (aminopeptidase p) isoform cra_a	F:GO:0008233		
01551_Oc_5_008	Embryo specific expression	gi|119789144|gb|EH281703.1|EH281703	6.00E-08	57.9	transmembrane emp24 domain-containing protein 5 precursor	C:GO:0016021	EMP24_GP25L	
01657_Oc_5_021		gi|37633733|gb|CF737397.1|CF737397	3.00E-26	118	mgc68950 protein	P:GO:0040023	CAP_GLY	0.69648

01718_Oc_5_091	Embryo specific expression	gi|83525044|gb|DB294194.1|DB294194	0	193	riken cdna 1700022n24	F:GO:0008270,F:GO:0005515,C:GO:0016021	Pfam-B_14559	0.20541
01737_Oc_5_005		gi|22696303|gb|BU182319.1|BU182319	0	228	chaperonin subunit 3	P:GO:0006457,C:GO:0005737,F:GO:0051082,F:GO:0005524	Cpn60_TCP1	0.9165
01791_Oc_5_056	GO annotation	gi|32463972|gb|CD805146.1|CD805146	8.00E-24	58.3	high mobility group b3b protein	P:GO:0006355,C:GO:0000785,F:GO:0003677,C:GO:0005634	HMG_box	
01826_Oc_5_009		gi|18965694|gb|BM661719.1|BM661719	2.00E-09	62.5	upregulated during skeletal muscle growth 5	C:GO:0005739,C:GO:0016021,C:GO:0016020,C:GO:0005743	Pfam-B_8509	
01855_Oc_5_024	Positive selection	gi|62888698|gb|DN903935.1|DN903935	0	200	cytochrome c proximal	F:GO:0045155,F:GO:0005506,C:GO:0005759,F:GO:0020037,P:GO:0006118,P:GO:0006810,C:GO:0005746	Cytochrom_C	10.1348
01868_Oc_5_046		BY796180	2.00E-07	50.6	---NA---			
01873_Oc_5_049		gi|19018745|gb|BM705487.1|BM705487	9.00E-35	90.9	signal recognition particle 14kda (homologous alu rna binding protein)	P:GO:0009987,F:GO:0005488,C:GO:0048500	SRP14	1.77853
01897_Oc_5_069		gi|19397619|gb|BM938467.1|BM938467	7.00E-29	127	riboflavin kinase	F:GO:0008531,P:GO:0009231,F:GO:0005524,F:GO:0000287,F:GO:0008270	Flavokinase	
01915_Oc_5_086		gi|62881909|gb|DN897146.1|DN897146	9.00E-12	47.8	---NA---			
02082_Oc_5_073		gi|46825597|gb|CN512973.1|CN512973	3.00E-26	115	proteasome 26s non-atpase subunit 9	C:GO:0043234,C:GO:0005829,P:GO:0008150,F:GO:0005515	PDZ	0.2745
02158_Oc_5_047	GO annotation	gi|24725639|gb|CA392686.1|CA392686	1.00E-05	47.3	zinc finger with krab and scan domains 4		zf-C2H2	

02222_Oc_5_015		gi|62880837|gb|DN896074.1|DN896074	3.00E-24	66.6	af498232_1peroxiredoxin v protein	F:GO:0016491	Redoxin	1.10022
02252_Oc_5_046		gi|10202777|gb|BE781579.1|BE781579	2.00E-05	46	---NA---			
02461_Oc_5_055	**Positive selection**	gi|41342286|gb|CK621400.1|CK621400	4.00E-15	60.2	transmembrane protein 33		UPF0121	3.5469
02464_Oc_5_064		gi|85195332|gb|DW603494.1|DW603494	2.00E-32	139	sulfide:quinone oxidoreductase			1.03437
02511_Oc_5_008		gi|14976737|gb|BI300457.1|BI300457	0.008	44.1				
02601_Oc_5_005	**Embryo specific expression**	gi|18266931|gb|BM413635.1|BM413635	0.006	25.8				
02672_Oc_5_080		gi|85198934|gb|DW604770.1|DW604770	0.0002	48.1				
02680_Oc_5_092		gi|19001706|gb|BM688448.1|BM688448	3.00E-12	71.6	marco_mesauame: full=macrophage receptor marcoame: full=macrophage receptor with collagenous structure	C:GO:0016020,F:GO:0004872	SRCR	
02683_Oc_5_086		gi|24727425|gb|CA393620.1|CA393620	6.00E-25	104	sec6 protein		Sec6	0.95688
02731_Oc_5_038	**Positive selection**	gi|41338923|gb|CK618037.1|CK618037	0	198	briggsae cbr-ncx-3 protein		Arf	2.9608
02792_Oc_5_012		gi|20365123|gb|BQ189572.1|BQ189572	6.00E-21	101			Na_Ca_ex	0.7284
02894_Oc_5_015		gi|40321164|gb|CK355232.1|CK355232	8.00E-34	95.5	chaperonin containingsubunit 8	P:GO:0044267,F:GO:0005488	Cpn60_TCP1	0.60428

02945_Oc_5_065	**Embryo specific expression**	gi|19116584|gb|BM799761.1|BM799761	0	233	rab5 protein		Ras	1.82924
02990_Oc_5_015		gi|75850467|gb|DT880727.1|DT880727	9.00E-19	64.3	---NA---	F:GO:0003779		0.0757
03014_Oc_5_043		BY795503	0.006	38.2				
03026_Oc_5_057		gi|57592584|gb|CX565555.1|CX565555	0	284	syndecan bindingisoform cra_a	C:GO:0005895,F:GO:0016491,F:GO:0005137,C:GO:0042470,F:GO:0047485,P:GO:0008152,F:GO:0046982,P:GO:0007265,F:GO:0042043	PDZ	0.2029
03032_Oc_5_060		gi|15711610|gb|BI734597.1|BI734597	4.00E-12	71.2	bromodomain and phd finger-containing protein 1 isoform 1	C:GO:0005622,P:GO:0006829,F:GO:0008270,F:GO:0005515		
03145_Oc_5_069		BY799086	0.001	40.1				
03192_Oc_5_028		gi|13063784|gb|BG298784.1|BG298784	1.40E-45	182	carnitine o-octanoyltransferase	C:GO:0005777,F:GO:0008458,P:GO:0006810,P:GO:0006631		1.94932
03241_Oc_5_069	**Positive selection**	gi|85208728|gb|DW608617.1|DW608617	0	343	dihydrolipoamide dehydrogenase	P:GO:0045454,F:GO:0050660,F:GO:0004148	Pyr_redox_dim, Pyr_redox_2	2.30203
03247_Oc_5_072		gi|41341343|gb|CK620457.1|CK620457	0.007	41.4	paf1 rna polymerase ii complex component	F:GO:0005515		
03249_Oc_5_081		gi|37634296|gb|CF737959.1|CF737959	3.00E-38	159	dishevelled associated activator of morphogenesis 1 like	P:GO:0016043,F:GO:0005515		
03250_Oc_5_089		gi|83528083|gb|DB294582.1|DB294582	0.008	44.1				
03253_Oc_5_083		gi|62046044|gb|DN691552.1|DN691552	4.00E-40	165	sorting nexin 4			

03428_Oc_5_074	gi|85206200|gb|DW607704.1|DW607704	0	248	phosphate carriermitochondrial precursor		Mito_carr	0.27196
03440_Oc_5_080	gi|18987504|gb|BM677608.1|BM677608	0.002	46.1				
03467_Oc_5_006	GS004812	0.008	32.2				
03515_Oc_5_054	gi|41342643|gb|CK621757.1|CK621757	0	134	heat shock protein 90kda betamember 1		HSP90	0.11
03518_Oc_5_063	gi|46823305|gb|CN510681.1|CN510681	1.00E-26	120	nop10 ribonucleoprotein homolog	C:GO:0005634	Nop10p	0.8304
03533_Oc_5_071	gi|11529949|gb|BF460792.1|BF460792	1.00E-28	127	rna-binding protein nova-		KH_1	0.65403
03597_Oc_5_039	gi|76234086|gb|CJ133758.1|CJ133758	2.00E-35	96.4	ribosomal protein l34	C:GO:0005840,F:GO:0003735,P:GO:0006412	Ribosomal_L34e	1.64326
03698_Oc_5_057	gi|62843102|gb|DN873161.1|DN873161	0	190	transaldolase 1	C:GO:0005737,F:GO:0005515,F:GO:0004801,P:GO:0006098	Transaldolase	0.16238
03711_Oc_5_056	gi|40320392|gb|CK354460.1|CK354460	0.007	44.1				
03771_Oc_5_022	gi|57592316|gb|CX565287.1|CX565287	4.00E-07	51.8	heat shock protein 90kda betamember 1		HSP90	
03840_Oc_5_096	gi|32273706|gb|CD722858.1|CD722858	2.00E-12	49.6	fatty acid amide hydrolase	F:GO:0016884,C:GO:0016021,F:GO:0016787,C:GO:0005615,C:GO:0016020,C:GO:0005783	Amidase	
04021_Oc_5_083	gi|18986794|gb|BM676898.1|BM676898	0.004	36.3			Ribosomal_S4Pg	
04055_Oc_5_020	gi|19018438|gb|BM705180.1|BM705180	4.00E-09	62	mgc82791 protein	C:GO:0005737,P:GO:0030100,F:GO:0005515	BAR	

04073_Oc_5_037		gi|22346578|gb|BQ931547.1|BQ931547	2.00E-11	66.5	protein disulfide isomerase			
04200_Oc_5_076		BY800402	0.005	32.7				
04207_Oc_5_072		BY795432	0.003	27.6				
04320_Oc_5_096		gi|58060652|gb|CX733816.1|CX733816	0	187	elongation factor tu			
04396_Oc_5_078	Embryo specific expression	gi|62871980|gb|DN887217.1|DN887217	0	213	adenylate kinase	F:GO:0005524,F:GO:0004017,P:GO:0006139	ADK	0.76786
04423_Oc_5_004		gi|75846801|gb|DT877061.1|DT877061	0.005	44.1				
04466_Oc_5_057	Embryo specific expression	gi|62037377|gb|DN687251.1|DN687251	0	216	thymidine kinaseisoform cra_b	C:GO:0005743,F:GO:0004797,P:GO:0006139,F:GO:0000166	Pfam-B_4425, dNK	1.93953
04550_OC_5_043		gi|22707146|gb|BU193170.1|BU193170	0.004	38.6				
04609_OC_5_001		GS008140	0.002	32.2			Pfam-B_587, Pfam-B_14082, Pfam-B_8988, Pfam-B_18484, Pfam-B_18104, Pfam-B_11111, Pfam-B_14589	
04613_OC_5_003		gi|75846808|gb|DT877068.1|DT877068	9.00E-18	71.6	wd repeat domain 74			
04619_OC_5_006		gi|22667936|gb|BU154404.1|BU154404	0.004	44.1				
04636_OC_5_030		gi|62853575|gb|DN883620.1|DN883620	8.00E-32	137	mitochondrial 28s ribosomal protein s28			1.25635

04654_OC_5_047	gi|16046496|gb|BI872821.1|BI872821	0.0002	35.9	---NA---		Pfam-B_11882	0.16963
04658_OC_5_057	gi|62832853|gb|DN863204.1|DN863204	0.0005	48.1			Pfam-B_2397	
04670_OC_5_063	gi|19039801|gb|BM720506.1|BM720506	3.00E-07	56.1	---NA---		CortBP2, Pfam-B_14732, Peptidase_M17	
04681_OC_5_069	gi|40322049|gb|CK356117.1|CK356117	0	200	tho complex subunit 7 homolog			0.8632
04754_Oc_5_057	gi|15710462|gb|BI733449.1|BI733449	0	209	kinh_lolpeame: full=kinesin heavy chain	P:GO:0007018,C:GO:0005875,F:GO:0005524,F:GO:0003777,C:GO:0005874	Pfam-B_11578	
04808_Oc_5_012	GS014047	0.01	32.2				
04810_Oc_5_013	gi|15009600|gb|BI326003.1|BI326003	0	153	mkiaa0109 protein	P:GO:0006461,F:GO:0008289,F:GO:0005515,F:GO:0008565,P:GO:0016192,P:GO:0006886,C:GO:0030141,C:GO:0030131,C:GO:0005739,C:GO:0030132	Clat_adaptor_s	0.27841
04856_Oc_5_060	BY798495	0.009	38.2				
04920_Oc_5_028	gi|75833046|gb|DT863306.1|DT863306	0.003	44.1				
04999_Oc_5_004	gi|8984832|gb|BB284383.1|BB284383	1.00E-11	54.2	rieske (fe-s) domain containing		Rieske	
05143_Oc_5_052	gi|18523576|gb|BM474534.1|BM474534	3.00E-12	72.5			Plexin_cytopl	

05231_Oc_5_040		gi|15586593|gb|BI671209.1|BI671209	0.0007	44.6	aminopeptidase puromycin sensitive	P:GO:0006508,F:GO:0004179,F:GO:0008237,F:GO:0016787,F:GO:0008233,F:GO:0008270		
05276_Oc_5_094		gi|19004616|gb|BM691358.1|BM691358	9.00E-26	117	nadh dehydrogenase 1 alpha subcomplex 2 variant 1		Pfam-B_10394, L51_S25_CI-B8	1.24034
05440_Oc_5_064		gi|49675657|gb|CO429363.1|CO429363	0	238	vacuolar atp synthase subunit d 1	C:GO:0016469,F:GO:0046961	vATP-synt_AC39	0.5621
05443_Oc_5_066		gi|54443551|gb|CV557712.1|CV557712	6.00E-35	147	baculoviral iap repeat-containing 6			
05555_Oc_5_082	**Embryo specific expression**	gi|85194722|gb|DW603282.1|DW603282	8.00E-33	140	gtp-binding protein 8	F:GO:0005525,C:GO:0005622		1.0748
05713_Oc_5_049		gi|57592048|gb|CX565019.1|CX565019	2.00E-16	85.8	fk506 binding proteinpartial	F:GO:0005515	FKBP_C	
05819_Oc_5_054		gi|46462071|gb|CN456345.1|CN456345	1.00E-28	127	ring finger protein 113a			0.0448
05962_Oc_5_013		gi|75851959|gb|DT882219.1|DT882219	1.00E-24	112	barrier-to-autointegration factor	C:GO:0005634	BAF	
06098_Oc_5_057		gi|46816556|gb|CN503932.1|CN503932	2.00E-10	67.9			eIF3_subunit	
06176_Oc_5_032		gi|24816591|gb|CA452171.1|CA452171	1.00E-14	80.3	zinc finger protein 271 (zinc finger protein 7) (zinc finger protein znfphex133) (epstein-barr virus-induced zinc finger protein) (znf-eb) (ct-zfp48) (zinc finger protein dp) (znf-dp)			

06182_Oc_5_043	**Embryo specific expression**	gi|18529315|gb|BM480273.1|BM480273	8.00E-09	57.4	lim domain-containing			
06249_Oc_5_005		gi|46824386|gb|CN511762.1|CN511762	3.00E-39	160	tata box binding protein-associated		TFIID-18kDa	0.31415
06263_Oc_5_020		gi|62839671|gb|DN869730.1|DN869730	0.004	40			Pfam-B_980	
06339_Oc_5_002		gi|15684799|gb|BI709104.1|BI709104	5.00E-16	84.9	cm037_xenlaame: full=upf0582 protein c13orf37 homolog		DUF3743	
06431_Oc_5_088		gi|62842908|gb|DN872967.1|DN872967	1.00E-07	57.4	galectin-4 (galactoside-soluble 4)	F:GO:0005529		
06480_Oc_5_048		gi|19011060|gb|BM697802.1|BM697802	3.00E-18	92.7	myosin iiia	C:GO:0030175,F:GO:0060002,F:GO:0005516,F:GO:0043531,F:GO:0030898,C:GO:0031941,P:GO:0007605,P:GO:0046777		
06506_Oc_5_077		gi|85205891|gb|DW607568.1|DW607568	0	222	calcium-transporting atpase 2c1			
06537_Oc_5_005		gi|41348034|gb|CK627148.1|CK627148	9.00E-22	104	nucleobindin 2b	F:GO:0005509		0.1616
06540_Oc_5_014		gi|37598621|gb|CF724453.1|CF724453	1.00E-29	130	cell division cycle 40 homolog	P:GO:0051301		0.2629
06561_Oc_5_033		GS008194	0.004	32.2			CAF1A, Pfam-B_8504, Pfam-B_15738, Nop14, Pfam-B_11288, Pfam-B_14006, Pfam-B_7566	

06590_Oc_5_063	gi|10199396|gb|BE778198.1|BE778198	0.005	29.9				
06592_Oc_5_064	gi|10204941|gb|BE783743.1|BE783743	0	138	karyopherin alpha 2 (rag cohortimportin alpha 1)	P:GO:0006886,C:GO:0005634	IBB, Arm	
06646_Oc_5_027	BY798958	3.00E-41	163	eukaryotic initiation factor 5c		W2	0.55447
06647_Oc_5_020	gi|16491534|gb|BB657708.1|BB657708	1.00E-28	126	small nuclear rna activatingpolypeptide 1	F:GO:0003674,P:GO:0006350,P:GO:0008150,C:GO:0005634,F:GO:0003677	SNAPc_SNAP43	0.47385
06682_Oc_5_061	gi|54479963|gb|CV574907.1|CV574907	1.00E-40	165	dead (asp-glu-ala-asp) box polypeptide 18	F:GO:0005524,F:GO:0008026		
06730_Oc_5_013	gi|85205936|gb|DW607590.1|DW607590	7.00E-32	117	manba_boviname: full=beta-mannosidaseame: full=lysosomal beta a mannosidaseame: full=mannanase short=mannase flags: precursor	P:GO:0008152	Pfam-B_67	
06784_Oc_5_064	GS004826	0.009	32.2			ATP-synt_E	
06847_Oc_5_024	gi|32465408|gb|CD806582.1|CD806582	3.00E-27	122	ferroxidaseprecursor - rat		Cu-oxidase_3	1.2635
06955_Oc_5_038	gi|83528618|gb|DB294616.1|DB294616	1.00E-22	106	bcl-2 inhibitor of transcription	F:GO:0004045,C:GO:0005739	PTH2, Pfam-B_7718	
06969_Oc_5_053	gi|62872333|gb|DN887570.1|DN887570	2.00E-35	87.7	mgc81115 protein			
07033_Oc_5_021	gi|23655182|gb|BU730863.1|BU730863	1.00E-22	71.2	isoform cra_a			1.34866
07075_Oc_5_066	gi|62828429|gb|DN858780.1|DN858780	0	276	heat shock protein 70	F:GO:0005524,P:GO:0006950	Pfam-B_2482, HSP70	

07131_Oc_5_022		GS013851	4.00E-05	40.1				
07343_Oc_5_040		BY797798	0.007	38.2			PLAC8	
07345_Oc_5_049		gi|62842025|gb|DN872084.1|DN872084	2.00E-10	36.8	gelsolin-like protein	F:GO:0003779	Gelsolin	0.59383
07379_Oc_5_082		gi|119789412|gb|EH281971.1|EH281971	0.0001	32.2	glutathione s-transferase		GST_N	
07421_Oc_5_023		gi|62839565|gb|DN869624.1|DN869624	8.00E-23	97.3	mitochondrial fission 1 protein	F:GO:0005488	TPR_1	0.86984
07462_Oc_5_075		gi|32180796|gb|CD679065.1|CD679065	2.00E-29	129	pre-mrna-splicing factor syf2 (ccndbp1-interactor)			0.2483
07467_Oc_5_070	**Positive selection**	gi|22802866|gb|BU500987.1|BU500987	0	291	mgc80278 protein			5.49096
07471_Oc_5_072		gi|23679207|gb|BU740075.1|BU740075	0.008	44.1				
07483_Oc_5_086		gi|37638276|gb|CF741937.1|CF741937	1.00E-07	57.4	ubiquitin specific peptidase 30			
07502_Oc_5_015		gi|32273237|gb|CD722389.1|CD722389	2.00E-22	105	aldehyde dehydrogenase 3member d1	P:GO:0006081,F:GO:0016491,F:GO:0004030,C:GO:0005575	Aldedh	0.7517
07566_Oc_5_079		gi|62040485|gb|DN688816.1|DN688816	0	118	acyl-synthetase bubblegum family member 2	F:GO:0016874,P:GO:0006629		0.33408
07568_Oc_5_080		gi|54471416|gb|CV570665.1|CV570665	0.008	44.1				
07602_Oc_5_025		gi|9127816|gb|BE257342.1|BE257342	5.00E-36	115	phosducin-like 3	C:GO:0005737,F:GO:0005515	Phosducin	0.6116
07691_Oc_5_006		gi|62841082|gb|DN871141.1|DN871141	0	221	atp synthase gamma		ATP-synt	0.64806

07693_Oc_5_007		gi|85200581|gb|DW605304.1|DW605304	2.00E-08	60.2	thiolester containing protein ii isoform c			
07712_Oc_5_032	GO annotation	gi|13270222|gb|BG373685.1|BG373685	2.00E-29	129	hla-b associated transcript 1	F:GO:0003676,F:GO:0005524,F:GO:0008026		1.01186
07748_Oc_5_074		gi|85205535|gb|DW607393.1|DW607393	3.00E-19	94.5	afg3(atpase family gene 3)-like 1			0.67195
07796_Oc_5_026		gi|58035650|gb|CX733176.1|CX733176	8.00E-11	67.5	coactivator-associated arginine methyltransferase 1	P:GO:0006355,P:GO:0030518,F:GO:0003713,F:GO:0008276,P:GO:0016571		
07841_Oc_5_065	Positive selection	gi|19353921|gb|BM903952.1|BM903952	0	163	tumor suppressor candidate 3			2.00297
07846_Oc_5_075		gi|45305571|gb|CK875940.1|CK875940	6.00E-05	47.8	mitochondrial transcription factorisoform d	F:GO:0003712,C:GO:0005759,P:GO:0006390	Coiled-coil_56	
07897_Oc_5_021		gi|75842858|gb|DT873118.1|DT873118	4.00E-10	65.2	amphiphysin bar domain from drosophila		BAR	
07917_Oc_5_039		GS014086	8.00E-05	38.2				
07921_Oc_5_049		gi|62872616|gb|DN887853.1|DN887853	0.01	35				
07944_Oc_5_076		gi|21763104|gb|BQ638645.1|BQ638645	0	232	heat shock protein 90		HSP90	
07952_Oc_5_080		gi|85201773|gb|DW605722.1|DW605722	3.00E-35	105	brix domain containing 1		ERO1	1.09096
07976_Oc_5_012		gi|10199520|gb|BE778322.1|BE778322	1.00E-19	96.4	endoplasmic oxidoreductin-1			0.3883
08025_Oc_5_053		gi|62049453|gb|DN693253.1|DN693253	2.00E-16	86.3	cg14782-pa isoform 2			

08072_Oc_5_012		gi|22709767|gb|BU195783.1|BU195783	0	316	clathrin coat assembly protein		Clat_adaptor_s	0.03167
08078_Oc_5_015		gi|85572315|gb|DW712123.1|DW712123	0	242	af266220_1proteasome subunit beta type 1	C:GO:0005839,F:GO:0005515,F:GO:0004298,P:GO:0006511,C:GO:0005634	Proteasome	0.5332
08081_Oc_5_017		gi|62882572|gb|DN897809.1|DN897809	2.00E-34	145	na+ k+ atpase alpha subunit	F:GO:0015077,F:GO:0019870,F:GO:0005267,F:GO:0030955,C:GO:0005579,P:GO:0006813,P:GO:0008152,F:GO:0015662,F:GO:0000166	Cation_ATPase_C	0.32584
08120_Oc_5_060		gi|62041039|gb|DN689091.1|DN689091	2.00E-18	92.7	elegans proteinpartially confirmed by transcript evidence	P:GO:0007242,F:GO:0005515	C1_1	
08204_Oc_5_046		BY798237	0.009	26.3				
08268_Oc_5_014		gi|58061282|gb|CX734446.1|CX734446	1.00E-11	70.3	---NA---			0.80885
08322_Oc_5_073		gi|10202867|gb|BE781741.1|BE781741	3.00E-24	112	af393635_1visual arrestin	P:GO:0007600,P:GO:0007165	Arrestin_N	0.34364
08352_Oc_5_096		gi|62827577|gb|DN857928.1|DN857928	3.00E-06	52				
08363_Oc_5_006		GS020748	0.007	32.2				
08462_Oc_5_015		gi|85203731|gb|DW606507.1|DW606507	0.0001	47.3	gelsolin-like protein	F:GO:0003779	Gelsolin	1.40875
08618_Oc_5_077		gi|5034328|gb|AI717072.1|AI717072	0	187	proteaseome (prosome macropain) 28 subunit 3			0.22146
08656_Oc_5_016	**Embryo specific expression**	gi|46565708|gb|CN484204.1|CN484204	0	219	icarus cg9031-pa		Pfam-B_6730	0.14439

08705_Oc_5_065		gi|32177391|gb|CD675660.1|CD675660	2.00E-08	59.7	novel protein with a dna polymerasesubunit 4 domain	P:GO:0006260,C:GO:0005634	DNA_pol_delta_4	
08730_Oc_5_093		gi|29527710|gb|CB584259.1|CB584259	0	114	heat shock protein 5	C:GO:0005788,F:GO:0005515,C:GO:0042470,F:GO:0005524,P:GO:0006983,F:GO:0043022	Pfam-B_2482, HSP70	
08731_Oc_5_086		gi|19353895|gb|BM903943.1|BM903943	0	215	glutamyl-prolyl-trna synthetase	C:GO:0005737,F:GO:0004827,P:GO:0006433,F:GO:0004818,P:GO:0006424,F:GO:0005524	HGTP_anticodon	
08745_Oc_5_005		gi|32173164|gb|CD671424.1|CD671424	3.00E-11	68.9	williams-beuren syndrome chromosome region 16 homolog		RCC1	
08767_Oc_5_024		gi|13054197|gb|BG294000.1|BG294000	0.01	40.5	cell adhesion molecule	F:GO:0005488	fn3	
08785_Oc_5_049		gi|14063071|gb|BG752418.1|BG752418	0.0001	50.1				
08786_Oc_5_057		gi|22274904|gb|BQ882896.1|BQ882896	0	118	malic enzyme		Malic_M	0.65495
08812_Oc_5_078	**Embryo specific expression**	gi|13298391|gb|BG373919.1|BG373919	0.006	44.1				
08845_Oc_5_007		gi|85196168|gb|DW603790.1|DW603790	1.00E-16	86.3	nmda receptor glutamate-binding chain isoform 2		UPF0005	
08871_Oc_5_036	**Embryo specific expression**	BY800203	0.009	38.2	sperm associated antigenpartial	F:GO:0005525,P:GO:0007338,F:GO:0005488,F:GO:0016787,F:GO:0000166		0.92387

08912_Oc_5_080		gi|19016174|gb|BM702916.1|BM702916	8.00E-14	76.7	calcyclin binding protein	C:GO:0005737,P:GO:0006512,C:GO:0005634	SGS	
08917_Oc_5_083		BY800072	0.0004	40.1				
08953_Oc_5_021		gi|46817532|gb|CN504908.1|CN504908	2.00E-40	146	coiled-coil domain-containing protein 58	P:GO:0008150,C:GO:0005575	Cid2	0.70103
08954_Oc_5_029		gi|19028683|gb|BM715425.1|BM715425	0.0004	45.1				
08991_Oc_5_056		gi|75846603|gb|DT876863.1|DT876863	1.00E-39	163	glutathione s-transferase theta 1			0.61475
09022_Oc_5_095		gi|41345608|gb|CK624722.1|CK624722	9.00E-08	57	transmembrane protein 161b		Pfam-B_13263, Tmemb_161AB	
09100_Oc_5_078		gi|9123773|gb|BE253612.1|BE253612	0	123	sec13-like 1		WD40	
09121_Oc_5_001		gi|62840342|gb|DN870401.1|DN870401	1.40E-45	183	exosome complex exonuclease			0.427
09132_Oc_5_014		gi|57591990|gb|CX564961.1|CX564961	1.00E-15	83.5	alveolar soft part sarcoma chromosomecandidate 1			
09156_Oc_5_042		BY797489	0.01	36.2				
09165_Oc_5_039		gi|32172937|gb|CD671206.1|CD671206	0	226	inositol-3-phosphate synthase a	P:GO:0006021,F:GO:0005488,P:GO:0008654,F:GO:0004512	NAD_binding_5	0.16312
09168_Oc_5_048	**Embryo specific expression**	gi|50134996|gb|CO469880.1|CO469880	0	305	na+ k+ atpase alpha subunit	F:GO:0015077,F:GO:0005391,F:GO:0030955,F:GO:0005524,F:GO:0000287,F:GO:0031402,P:GO:0006813,C:GO:0016021,P:GO:0008152,P:GO:0006814	Cation_ATPase_C	0.42477

09189_Oc_5_067		gi|19390803|gb|BM931630.1|BM931630	0.008	44.1				
09225_Oc_5_005		gi|50138203|gb|CO471352.1|CO471352	0	200	translation initiation factor 1a			0.65996
09243_Oc_5_022		gi|62852808|gb|DN882853.1|DN882853	0.002	46.1			Pfam-B_6449	
09302_Oc_5_091		gi|46823183|gb|CN510559.1|CN510559	1.00E-27	121	ribosomal protein l	C:GO:0005840,F:GO:0003735,P:GO:0006412	Ribosomal_L2_C	
09357_Oc_5_039	**Embryo specific expression**	gi|126524124|gb|EL647843.1|EL647843	0	194	serine threonine tyrosine-interacting protein	P:GO:0006470,F:GO:0008138		
09365_Oc_5_051		gi|54440614|gb|CV556259.1|CV556259	0.002	46.1				
09370_Oc_5_061		gi|75844397|gb|DT874657.1|DT874657	0	249	peroxisomal trans-2-enoyl-reductase	F:GO:0005488,F:GO:0016491,P:GO:0008152,F:GO:0003824		0.25225
09412_Oc_5_010	**Embryo specific expression**	gi|85197393|gb|DW604221.1|DW604221	1.00E-07	57.4	kv channel-interacting protein			
09429_Oc_5_019		gi|11672321|gb|BF562591.1|BF562591	0.008	44.1				
09491_Oc_5_082		gi|85203729|gb|DW606506.1|DW606506	8.00E-11	68	nucleoporin 50	C:GO:0016020,P:GO:0046907		
09492_Oc_5_090		gi|119778776|gb|EH278374.1|EH278374	0.01	41				
5primeCluster0002.Contig1		BY795134	4.00E-09	38.2	cg6180-pa isoform 2		PBP	
5primeCluster0002.Contig3		gi|13406406|gb|BG474129.1|BG474129	2.00E-19	96.7	cg6180-pa isoform 2		PBP	0.72476

5primeCluster0008.Contig2		gi|22353925|gb|BQ938447.1|BQ938447	0	105	cytochrome c oxidase subunit ii	F:GO:0046872,C:GO:0005743,C:GO:0044425	COX2_TM, COX2	0.61197
5primeCluster0009.Contig1		gi|62046021|gb|DN691540.1|DN691540	0	208	crom_octdoame: full=omega-crystallin	F:GO:0005212,F:GO:0016491,P:GO:0008152	Aldedh	1.32732
5primeCluster0021.Contig1		BY794962	6.00E-06	46.4	atp synthetase subunit 6	C:GO:0016020	ATP-synt_A	
5primeCluster0031.Contig1		gi|14261392|gb|BG884300.1|BG884300	5.00E-25	107	ribosomal protein lp1	C:GO:0005840,F:GO:0003735,P:GO:0006414	Ribosomal_60s	1.31863
5primeCluster0036.Contig1		BY794962	0.0002	41.4	atp synthetase subunit 6	C:GO:0044464	ATP-synt_A	
5primeCluster0045.Contig3	**Positive selection**	gi|62873711|gb|DN888948.1|DN888948	0	166	---NA---		TMF_DNA_bd, ATG16, Tropomyosin, ERM, Pfam-B_16520, Tropomyosin, Myosin_tail_1, IncA	4.01906
5primeCluster0046.Contig1		gi|54470231|gb|CV570080.1|CV570080	4.00E-13	46	saposin isoform 1		Pfam-B_328, SapB_1, SapB_2	
5primeCluster0048.Contig1		gi|75839196|gb|DT869456.1|DT869456	1.00E-13	47.8	histone h1	C:GO:0005694	Linker_histone	0.15023
5primeCluster0050.Contig2		gi|41341803|gb|CK620917.1|CK620917	0.0008	35.4	calmodulin 4 (calcium-binding protein dd112)	F:GO:0005509,C:GO:0005626,C:GO:0005625	efhand	
5primeCluster0052.Contig1		GS020536	0.003	34.2				

5primeCluster0052.Contig3		GS020536	0.008	34.2	gastrotropin(fatty acid-binding protein 6) (ileal lipid-binding protein) (porcine ileal peptide)		Pfam-B_587, Pfam-B_14296 , Pfam-B_18747, Pfam-B_6927, Pfam-B_3553, Pfam-B_7642, Pfam-B_8096, Pfam-B_14082	
5primeCluster0059.Contig1		BY795013	0	230	ribosomal protein s15a	C:GO:0005840,F:GO:0003735,P:GO:0006412	Ribosomal_S8	0.47553
5primeCluster0059.Contig2		BY795013	0	254	ribosomal protein rps15a	C:GO:0005840,F:GO:0003735,P:GO:0006412	Ribosomal_S8	0.26852
5primeCluster0061.Contig1		gi|75839434|gb|DT869694.1|DT869694	7.00E-41	166	ribosomal protein s26	C:GO:0030529	Ribosomal_S26e	0.60336
5primeCluster0061.Contig2		gi|119779873|gb|EH278644.1|EH278644	1.00E-39	163	rs26_octvuame: full=40s ribosomal protein s26	C:GO:0030529	Ribosomal_S26e	0.06295
5primeCluster0062.Contig1		gi|24544553|gb|CA326455.1|CA326455	0	410	ribosomal protein s5	P:GO:0042254,F:GO:0003735,N:GO:0022627,P:GO:0006412	Ribosomal_S7	0.04041
5primeCluster0064.Contig1		gi|119777845|gb|EH278206.1|EH278206	0	210	ribosomal protein l10	C:GO:0005840,F:GO:0003735,P:GO:0006412	Ribosomal_L16	0.0922
5primeCluster0067.Contig1		BY795427	6.00E-14	67.5	aecombinant actophorin	C:GO:0005737,F:GO:0003779,C:GO:0005622	Pfam-B_8155, Cofilin_ADF	1.42294
5primeCluster0071.Contig1		gi|62828989|gb|DN859340.1|DN859340	2.00E-37	124	ribosomal protein s30	P:GO:0044267,C:GO:0005622	ubiquitin, Ribosomal_S30	0.45407
5primeCluster0071.Contig2		BY795000	3.00E-38	117	ribosomal protein s30	P:GO:0044267,C:GO:0005622	ubiquitin, Ribosomal_S30	0.18231
5primeCluster0073.Contig1	**Positive selection**	gi|37637396|gb|CF741058.1|CF741058	2.00E-41	168	60s ribosomal protein l23	C:GO:0005840,F:GO:0003735,P:GO:0006412	Ribosomal_L14	7.68894
5primeCluster0074.Contig1		gi|50140708|gb|CO472492.1|CO472492	2.00E-11	69.8	mitochondrial atp synthase f chain		WRW	1.11975

5primeCluster0075.Contig1		gi|119779534|gb|EH278551.1|EH278551	6.00E-24	113	transport protein sec61 subunit gamma	P:GO:0006886,C:GO:0016020	SecE	
5primeCluster0075.Contig2		gi|85183876|gb|DW600023.1|DW600023	4.00E-27	121	transport protein sec61 gamma subunit	P:GO:0065002,C:GO:0005789,F:GO:0015450,P:GO:0006605,C:GO:0016021	SecE	0.21347
5primeCluster0076.Contig1		gi|62854210|gb|DN884255.1|DN884255	2.00E-23	108	40s ribosomal protein s29	C:GO:0005840,F:GO:0003735,F:GO:0008270,P:GO:000641	Ribosomal_S14	
5primeCluster0076.Contig2		gi|50140539|gb|CO472426.1|CO472426	2.00E-25	115	ribosomal protein s29	C:GO:0005840,F:GO:0003735,F:GO:0008270,P:GO:000641	Ribosomal_S14	1.94215
5primeCluster0077.Contig1		gi|10589311|gb|BE950645.1|BE950645	9.00E-29	117	ornithine decarboxylase antizymeisoform cra_a		ODC_AZ	0.36185
5primeCluster0077.Contig2		gi|10589311|gb|BE950645.1|BE950645	7.00E-28	113	ornithine decarboxylase antizyme	F:GO:0008073	ODC_AZ	1.534
5primeCluster0087.Contig1		gi|62031452|gb|DN684263.1|DN684263	0	141	cathepsin l		Inhibitor_I29, Peptidase_C1	
5primeCluster0088.Contig1		gi|40318508|gb|CK352621.1|CK352621	2.00E-39	162	translocon-associated protein subunit delta precursor	C:GO:0016021,C:GO:0005783	TRAP-delta, SEF14_adhesin	0.19836
5primeCluster0088.Contig2		gi|40318508|gb|CK352621.1|CK352621	0	207	translocon-associated protein subunit delta precursor	F:GO:0004872,C:GO:0016021,P:GO:0008150	TRAP-delta, SEF14_adhesin	0.34097
5primeCluster0091.Contig1	**Positive selection**	gi|62888533|gb|DN903770.1|DN903770	0	251	fatty aldehyde dehydrogenase		Aldedh	2.41432
5primeCluster0094.Contig1		gi|24559278|gb|CA341182.1|CA341182	0	210	ribosomal protein rpl26	F:GO:0003735,C:GO:0015934,P:GO:0006412	KOW	0.06808
5primeCluster0094.Contig2		gi|124701339|gb|EH999942.1|EH999942	0	221	ribosomal protein rpl26	F:GO:0003735,C:GO:0015934,P:GO:0006412	KOW	0.5299

5primeCluster0097.Contig1	gi|45305112|gb|CK875481.1|CK875481	0	187	60s ribosomal protein l35	P:GO:0009987,C:GO:0030529	Ribosomal_L29	1.3573
5primeCluster0097.Contig2	gi|91746892|gb|EB383547.1|EB383547	0	184	ribosomal protein l35		Ribosomal_L29	0.70752
5primeCluster0098.Contig1	gi|24559069|gb|CA340973.1|CA340973	0	222	60s ribosomal protein l30	C:GO:0005840,F:GO:0003735,P:GO:0006412	Ribosomal_L7Ae	
5primeCluster0103.Contig1	gi|46823889|gb|CN511265.1|CN511265	0	349	arp2 3 complex 20 kd subunit	C:GO:0005856,P:GO:0030041	ARPC4	0.62763
5primeCluster0104.Contig1	gi|23654355|gb|BU730451.1|BU730451	0	294	n-acetyltransferase 13	P:GO:0001756,C:GO:0005737,P:GO:0008152,F:GO:0008080	Acetyltransf_1	
5primeCluster0107.Contig1	gi|78181987|gb|DV552360.1|DV552360	9.81E-45	179	60s ribosomal protein l14		Pfam-B_12433, Ribosomal_L14e	0.2301
5primeCluster0107.Contig2	gi|78181987|gb|DV552360.1|DV552360	2.80E-45	182	ribosomal proteinpartial		Pfam-B_12433, Ribosomal_L14e	1.08392
5primeCluster0112.Contig1	gi|62889671|gb|DN904908.1|DN904908	0	308	ribosomal protein s16	P:GO:0000022,F:GO:0003735,C:GO:0005811,N:GO:0022627,P:GO:0006412	Ribosomal_S9	0.15204
5primeCluster0113.Contig1	gi|46818458|gb|CN505834.1|CN505834	8.00E-24	79	translationally-controlled tumor protein	P:GO:0006412,F:GO:0005509,C:GO:0005737,C:GO:0005874	TCTP	0.9258
5primeCluster0114.Contig1	gi|85190796|gb|DW602403.1|DW602403	0	203	ribosomal protein s24e	C:GO:0005840,F:GO:0003735,P:GO:0006412,F:GO:0000166	Ribosomal_S24e	1.31471
5primeCluster0114.Contig2	BY795029	2.94E-44	168	40s ribosomal protein s24	C:GO:0005840,F:GO:0003735,P:GO:0006412,F:GO:0000166	Ribosomal_S24e	1.54532
5primeCluster0120.Contig1	BY798636	4.00E-26	112	gaba-a receptor associated protein	F:GO:0004872	MAP1_LC3	0.95724

5primeCluster0128.Contig1		BY795095	7.00E-42	133	60s ribosomal protein l23a variant 1	C:GO:0005840,F:GO:0000166	Ribosomal_L23eN, Ribosomal_L23	1.48442
5primeCluster0129.Contig1		gi|19026532|gb|BM713274.1|BM713274	0	325	dolichyl-phosphate mannosyltransferase polypeptidecatalytic subunit	C:GO:0005789,F:GO:0004582,P:GO:0035269,P:GO:0006506,F:GO:0005515,F:GO:0004169	Glycos_transf_2	0.46204
5primeCluster0132.Contig2		gi|85182524|gb|DW599548.1|DW599548	0	365	heat shock cognate 70	F:GO:0005524,P:GO:0006950	HSP70	
5primeCluster0136.Contig2		gi|62878283|gb|DN893520.1|DN893520	0.003	42.3	linker histone h1 and h5 family protein	C:GO:0000786,P:GO:0006334,F:GO:0003677	Linker_histone, Pfam-B_9326, Pfam-B_14006Pfam-B_10197	
5primeCluster0136.Contig3		gi|57592021|gb|CX564992.1|CX564992	0.0002	46.9	linker histone h1 and h5 family protein	C:GO:0043229	Pfam-B_10197, Linker_histone, S10_plectin	
5primeCluster0137.Contig2	Embryo specific expression	gi|75844950|gb|DT875210.1|DT875210	0	135	ribosomal protein s10	C:GO:0005840		1.51048
5primeCluster0138.Contig1		gi|58035516|gb|CX733042.1|CX733042	7.00E-18	62	mitochondrial nadh:ubiquinone oxidoreductase		GRIM-19	0.78623
5primeCluster0143.Contig1		gi|126525667|gb|EL649386.1|EL649386	2.00E-42	172	ribosomal protein l11	C:GO:0030529	Ribosomal_L5_C	
5primeCluster0145.Contig1	Embryo specific expression, Positive selection	gi|39889198|gb|CK300140.1|CK300140	6.00E-41	168	glutathione s-transferase mu		GST_C	2.87141
5primeCluster0147.Contig1		gi|85171835|gb|DW596067.1|DW596067	0	249	peptidase (mitochondrial processing) beta isoform 2		Peptidase_M16_C	0.75579

5primeCluster0150.Contig1		gi|11534174|gb|BF464991.1|BF464991	1.00E-16	62.9	mgc88931 protein	C:GO:0000785,F:GO:0003677,C:GO:0005634,P:GO:0006355	HMG_box	
5primeCluster0151.Contig1		gi|22673837|gb|BU159927.1|BU159927	2.00E-31	136	cg10233-isoform a		MORN	0.25777
5primeCluster0153.Contig1		BY795011	0	148	ribosomal protein l21	C:GO:0005622	Ribosomal_L21e, Pfam-B_3820	1.84
5primeCluster0161.Contig1		gi|46819336|gb|CN506712.1|CN506712	0	155	atp synthase oligomycin sensitivity conferral protein		OSCP	1.31305
5primeCluster0163.Contig1		gi|13104757|gb|BG307230.1|BG307230	8.00E-28	76.2	60s ribosomal protein l22 (development-specific protein 217)	F:GO:0003735,P:GO:0006412,C:GO:0005840,C:GO:0005622	Ribosomal_L22e	0.83977
5primeCluster0165.Contig1		gi|77502748|gb|DV214656.1|DV214656	0	196	ribosomal protein s17	C:GO:0005840,F:GO:0003735,P:GO:0006412	Ribosomal_S17e	1.28725
5primeCluster0169.Contig1		gi|126526025|gb|EL649744.1|EL649744	1.00E-18	92.7	---NA---		MMgT	0.0725
5primeCluster0169.Contig2		gi|126526025|gb|EL649744.1|EL649744	1.00E-17	90	---NA---		MMgT, DUF962	
5primeCluster0173.Contig1	**Positive selection**	BY795137	7.00E-28	118	ribosomal protein s25	C:GO:0005840	Ribosomal_S25	3.18242
5primeCluster0174.Contig1		gi|41343480|gb|CK622594.1|CK622594	8.00E-16	83.5	cytochrome csubunit vic	C:GO:0005615,C:GO:0005751,C:GO:0016021,F:GO:0004129	COX6C	
5primeCluster0178.Contig1		gi|45305160|gb|CK875529.1|CK875529	9.00E-39	159	40s ribosomal protein s21		Ribosomal_S21e	1.61541
5primeCluster0178.Contig2		gi|58035040|gb|CX732566.1|CX732566	1.00E-38	159	ribosomal protein s21	F:GO:0003735,P:GO:0006412,C:GO:0030529	Ribosomal_S21e	1.95144
5primeCluster0184.Contig1		GS008194	0.005	32.2				
5primeCluster0188.Contig1	**Positive selection**	gi|75840163|gb|DT870423.1|DT870423	0	189	es1 protein		DJ-1_PfpI	2.2695

5primeCluster0191.Contig1		gi|49672561|gb|CO426267.1|CO426267	6.00E-13	74.8	heat shock protein gp96	P:GO:0006457,C:GO:0005788,C:GO:0042470,P:GO:0006950,F:GO:0005509,F:GO:0005524,F:GO:0051082	HSP90	
5primeCluster0192.Contig1		gi|40318338|gb|CK352451.1|CK352451	3.00E-06	34.5	zinc finger protein 346 variant 3		zf-C2H2_jaz	
5primeCluster0193.Contig1		gi|49672103|gb|CO425809.1|CO425809	3.00E-38	159	syntenin-1 (syndecan-binding protein 1) (melanoma differentiation-associated protein 9) (mda-9) (scaffold protein pbp1) (pro-tgf-alpha cytoplasmic domain-interacting protein 18)	F:GO:0005137,P:GO:0008152,C:GO:0005895,F:GO:0016491,P:GO:0007265	PDZ	0.78017
5primeCluster0197.Contig1		gi|32465408|gb|CD806582.1|CD806582	1.00E-31	81.4	---NA---		Cu-oxidase_3	
5primeCluster0202.Contig1	**GO annotation, Positive selection**	gi|13056621|gb|BG295212.1|BG295212	5.00E-41	168	fgfr1 oncogene partner 2	C:GO:0005737	DUF837	2.241
5primeCluster0204.Contig1		gi|37628162|gb|CF731832.1|CF731832	0	154	sucla2 protein	C:GO:0005739	ATP-grasp_2	0.35182
5primeCluster0205.Contig1	**Positive selection**	gi|62853918|gb|DN883963.1|DN883963	0	318	thioredoxin peroxidase 2	F:GO:0004601	AhpC-TSA, 1-cysPrx_C	7.62969
5primeCluster0208.Contig1		gi|62037240|gb|DN687181.1|DN687181	2.00E-32	59.8	---NA---		Transferrin	1.0693
5primeCluster0209.Contig1		GS020551	0.002	32.2				
5primeCluster0214.Contig2	**GO annotation**	gi|12042337|gb|BF726426.1|BF726426	0	202	annexin a7	F:GO:0005488,C:GO:0044424	Annexin	0.34138

5primeCluster0215.Contig1		gi|8487330|gb|BE096399.1|BE096399	5.00E-37	155	g alpha q subunit	P:GO:0007186,F:GO:0004871,P:GO:0006471,F:GO:0005525	G-alpha	0.42835
5primeCluster0219.Contig1		gi|18523312|gb|BM474270.1|BM474270	0.0002	46.5	fusion (involved in tin malignant liposarcoma) isoform a isoform 2			
5primeCluster0223.Contig1		gi|45305796|gb|CK876165.1|CK876165	3.00E-29	103	transport protein sec61 subunit beta		Sec61_beta	
5primeCluster0224.Contig1		gi|11536060|gb|BF466877.1|BF466877	6.00E-06	51.5	nuclear fragile x mental retardation protein interacting protein 1	F:GO:0003676,F:GO:0008270,C:GO:0005622	Pfam-B_6194, NUFIP1	
5primeCluster0227.Contig1		gi|76146130|gb|CJ065007.1|CJ065007	1.00E-37	156	ceruloplasmin precursorisoform 3		Cu-oxidase_2	0.5422
5primeCluster0235.Contig1		gi|37636035|gb|CF739697.1|CF739697	0	195	60s ribosomal protein l27a		Ribosomal_L18e	0.38194
5primeCluster0247.Contig1		gi|62842068|gb|DN872127.1|DN872127	7.00E-29	76.2	nadh dehydrogenase			
5primeCluster0251.Contig1		gi|32175032|gb|CD673301.1|CD673301	4.00E-10	46	isoform a		HSP20	0.5291
5primeCluster0259.Contig1	**Embryo specific expression**	gi|62883263|gb|DN898500.1|DN898500	2.00E-40	112	60s ribosomal protein rpl35a		Ribosomal_L35Ae	0.71661
5primeCluster0259.Contig2		gi|62883263|gb|DN898500.1|DN898500	1.40E-45	111	ribosomal protein rpl35a		Ribosomal_L35Ae	1.30327
5primeCluster0260.Contig1		gi|19004898|gb|BM691640.1|BM691640	2.00E-15	59.3	zgc:92867 protein	F:GO:0046872,C:GO:0005737,F:GO:0000287,F:GO:0016787,C:GO:0005634,F:GO:0004649	ADP_ribosyl_GH	
5primeCluster0265.Contig1		gi|18991779|gb|BM681883.1|BM681883	5.00E-10	64.8	cmc1 protein		Cmc1	

5primeCluster0266.Contig1		gi|50138916|gb|CO471675.1|CO471675	2.00E-13	76.2	heterogeneous nuclear ribonucleoprotein m	F:GO:0000166,P:GO:0008150,C:GO:0005575	RRM_1	
5primeCluster0273.Contig1	**Positive selection**	BY796454	1.00E-20	67	ribosomal protein rpl36a		Ribosomal_L44	2.43467
5primeCluster0275.Contig1		gi|62885542|gb|DN900779.1|DN900779	8.00E-07	53.3	sjchgc01974 protein			
5primeCluster0281.Contig1		BY799369	0.006	38.2				
5primeCluster0281.Contig2		BY800453	5.00E-06	48.1				
5primeCluster0289.Contig1		gi|19019796|gb|BM706538.1|BM706538	3.00E-36	124	arpc1a protein			
5primeCluster0291.Contig1		gi|62880016|gb|DN895253.1|DN895253	6.00E-25	76.1	beta chain spectrin		Pfam-B_1882, PH	0.17895
5primeCluster0295.Contig1		BY794950	0	132	60s ribosomal protein l13a	F:GO:0003735,C:GO:0015934,P:GO:0006412	Ribosomal_L13	0.29973
5primeCluster0295.Contig2		gi|13404527|gb|BG472153.1|BG472153	0	302	60s ribosomal protein l13a	F:GO:0003735,C:GO:0015934,P:GO:0006412	Ribosomal_L13	0.70766
5primeCluster0302.Contig1		gi|85183430|gb|DW599865.1|DW599865	0	197	diacylglycerol kinase beta (diglyceride kinase beta) (dgk-beta) (dag kinase beta) (90 kda diacylglycerol kinase)		DAGK_cat, DAGK_acc	0.97363
5primeCluster0308.Contig1		gi|46461359|gb|CN455633.1|CN455633	5.00E-34	144	mitochondrial ribosomal protein l18	F:GO:0003735,P:GO:0006412,C:GO:0005840,C:GO:0005622	Ribosomal_L18p	0.80462
5primeCluster0308.Contig2		gi|46461359|gb|CN455633.1|CN455633	1.00E-27	123	mitochondrial ribosomal protein l18	C:GO:0005615,C:GO:0044424	Ribosomal_L18p	0.67993

5primeCluster0311.Contig1	**Positive selection**	gi|14061563|gb|BG750910.1|BG750910	0	249	pp2a inhibitor	P:GO:0006334,C:GO:0005634	NAP, Phage_Mu_Gam	2.02108
5primeCluster0314.Contig1		gi|9121881|gb|BE251750.1|BE251750	0	200	beta like 1	C:GO:0005575	DUF1716	0.5801
5primeCluster0315.Contig1		gi|13299867|gb|BG375395.1|BG375395	2.00E-19	68.4	f-box proteinisoform cra_a	F:GO:0004386,F:GO:0005524,P:GO:0006281,F:GO:0000166,P:GO:0006512,C:GO:0005634,F:GO:0003677,F:GO:0004003	UvrD-helicase	
5primeCluster0321.Contig1	**Embryo specific expression**	gi|77506145|gb|DV216390.1|DV216390	1.00E-24	83.5	twins cg6235-isoform e isoform 1			1.228
5primeCluster0322.Contig1		gi|10202505|gb|BE781307.1|BE781307	2.00E-16	86.3	---NA---		Pfam-B_1551	
5primeCluster0324.Contig1		gi|124699704|gb|EH998307.1|EH998307	0	238	translation initiation factorhelicase		DEAD	
5primeCluster0328.Contig1	**Positive selection**	BY798303	0	139	cornichon homolog	C:GO:0043231,C:GO:0044444,P:GO:0007165,C:GO:0044425,P:GO:0006955	Cornichon	3.02565
5primeCluster0334.Contig1		gi|85165653|gb|DW593375.1|DW593375	0	188	fk506 binding protein 4	P:GO:0006457		0.12873
5primeCluster0335.Contig1		gi|46820693|gb|CN508069.1|CN508069	0	200	ribosome biogenesis regulatory protein homolog	F:GO:0003674,P:GO:0042254	RRS1	1.38976
5primeCluster0339.Contig1		gi|54471816|gb|CV570857.1|CV570857	1.00E-16	68.9	phosphoinositide-3-beta polypeptide	P:GO:0009987,P:GO:0065008,P:GO:0032501,F:GO:0035004	PI3Ka, PI3K_C2	
5primeCluster0341.Contig1		gi|62888766|gb|DN904003.1|DN904003	5.00E-26	98.7	methionine adenosyltransferasebeta		RmlD_sub_bind	0.4952
5primeCluster0344.Contig1		gi|37635813|gb|CF739476.1|CF739476	2.00E-13	76.2	cytochrome c oxidase polypeptide vb	C:GO:0005740,F:GO:0004129,P:GO:0006118	COX5B	0.95183

5primeCluster0344.Contig2	**Positive selection**	gi|58179118|gb|CX768765.1|CX768765	4.00E-13	75.3	cytochrome c oxidase polypeptide vb	C:GO:0005740,F:GO:0004129,P:GO:0006118	COX5B	2.75122
5primeCluster0347.Contig2	**Positive selection**	gi|62847435|gb|DN877480.1|DN877480	1.40E-45	182	glyceraldehyde-3-phosphate dehydrogenase	C:GO:0005737,F:GO:0051287,P:GO:0006096,F:GO:0004365	Gp_dh_C	2.9412
5primeCluster0350.Contig1		gi|49677623|gb|CO431329.1|CO431329	0.001	44.1	collagen alpha 1chain		Endostatin	
5primeCluster0355.Contig1	**Positive selection**	gi|14060102|gb|BG749449.1|BG749449	2.00E-37	155	kinesin light chain	F:GO:0003777,C:GO:0005874,F:GO:0005488,C:GO:0005871	Pfam-B_18958, Rab5-bind	3.34686
5primeCluster0357.Contig1		gi|77506418|gb|DV216527.1|DV216527	0	188	eukaryotic translation initiation factor 5a-1	P:GO:0006413,F:GO:0003743	eIF-5a, KOW	1.68893
5primeCluster0359.Contig1		BY797846	0.004	29.5				
5primeCluster0360.Contig1		gi|62827783|gb|DN858134.1|DN858134	0	188	---NA---		ELO	
5primeCluster0364.Contig1		gi|76146130|gb|CJ065007.1|CJ065007	5.00E-39	151	hephaestin isoform 1	P:GO:0030218,P:GO:0000041	Cu-oxidase_2	1.31773
5primeCluster0369.Contig1		gi|68325747|gb|DR423731.1|DR423731	0	279	ribosomal protein s11		Ribosomal_S17	
5primeCluster0371.Contig1		gi|46562702|gb|CN481198.1|CN481198	0.0004	45.5	---NA---		Thyroglobulin_1	0.86027
5primeCluster0373.Contig1		gi|57595569|gb|CX568540.1|CX568540	2.00E-16	76.2	regulator of g-protein signaling 4	F:GO:0004871	RGS	
5primeCluster0377.Contig1		gi|46854478|gb|CN526704.1|CN526704	9.00E-20	87.2	---NA---		EGF_CA	0.52845
5primeCluster0380.Contig1		gi|62883968|gb|DN899205.1|DN899205	0.006	41.8	ring finger-containing		zf-C3HC4, Pfam-B_14559	

5primeCluster0381.Contig1		gi|41338264|gb|CK617378.1|CK617378	0	193	shwachman-bodian-diamond syndrome homolog	F:GO:0003674,P:GO:0008150,C:GO:0005575	SBDS, SBDS_C	
5primeCluster0384.Contig1		gi|62041029|gb|DN689085.1|DN689085	1.00E-12	73.5	eukaryotic translation initiation factorsubunit 2partial		Peroxin-13_N	
5primeCluster0388.Contig1		gi|40321108|gb|CK355176.1|CK355176	0	128	mgc81086 protein	P:GO:0046439,F:GO:0046872,F:GO:0017172,P:GO:0006879,P:GO:0006826	CDO_I	1.26823
5primeCluster0390.Contig1		gi|54441716|gb|CV556808.1|CV556808	0	201	kinh_lolpeame: full=kinesin heavy chain	C:GO:0005737,P:GO:0007018,C:GO:0043227,P:GO:0007028,C:GO:0043005,F:GO:0005515,P:GO:0006839,C:GO:0035253,F:GO:0005524,C:GO:0005871,F:GO:0003777,C:GO:0005874	Pfam-B_7610	0.88919
5primeCluster0393.Contig1		gi|46819113|gb|CN506489.1|CN506489	0	326	mgc80364 protein	C:GO:0005839,F:GO:0004298,P:GO:0006511,C:GO:0005634	Proteasome	0.55173
5primeCluster0395.Contig1	**Positive selection**	gi|62841825|gb|DN871884.1|DN871884	0	127	heterogeneous nuclear ribonucleoprotein l	F:GO:0003676,F:GO:0000166,C:GO:0030529	RRM_1	2.48957
5primeCluster0398.Contig1		gi|62842819|gb|DN872878.1|DN872878	4.00E-36	151	mgc89819 protein	F:GO:0003674,C:GO:0005575	UMP1	0.32508
5primeCluster0403.Contig1		gi|85172771|gb|DW596524.1|DW596524	1.00E-07	57.4	loc563523 protein		Fibrinogen_C	
5primeCluster0405.Contig1		gi|62030807|gb|DN683939.1|DN683939	3.00E-16	63.4	hydroxysteroid dehydrogenase		3Beta_HSD	

5primeCluster0408.Contig1		gi|76050321|gb|CJ053284.1|CJ053284	3.00E-08	59.3	arystal structure of dimethylarginine dimethylaminohydrolase i in complexigh ph	F:GO:0016403,P:GO:0006809,F:GO:0008270,P:GO:0017014	Amidinotransf	
5primeCluster0412.Contig1		gi|85205026|gb|DW607143.1|DW607143	3.00E-11	68.9	adp-ribosylation factor 4		Arf	
5primeCluster0415.Contig1	**Embryo specific expression**	gi|62875839|gb|DN891076.1|DN891076	0	253	nadph-specific isocitrate dehydrogenase		Iso_dh	0.1571
5primeCluster0418.Contig1	**Embryo specific expression**	gi|54471554|gb|CV570733.1|CV570733	5.61E-45	181	sarco endoplasmic reticulum calcium transporting atpase	P:GO:0006816,F:GO:0005524,C:GO:0016021,P:GO:0008152,F:GO:0005388	Cation_ATPase_C	0.12589
5primeCluster0421.Contig1		gi|24722358|gb|CA390919.1|CA390919	0	200	hephaestin-like 1			0.35915
5primeCluster0433.Contig1		gi|75845182|gb|DT875442.1|DT875442	1.00E-29	130	transcription elongation factor b polypeptide 1			0.07255
5primeCluster0438.Contig1		gi|41346236|gb|CK625350.1|CK625350	2.00E-31	136	enhancer of yellow 2 homolog		EnY2	0.11278
5primeCluster0442.Contig1		gi|22700947|gb|BU186963.1|BU186963	6.00E-14	59.3	sema-1a cg18405-pa		Sema	
5primeCluster0444.Contig1		gi|62840545|gb|DN870604.1|DN870604	0.002	46.1				
5primeCluster0445.Contig1		gi|12040839|gb|BF724928.1|BF724928	2.00E-16	86.3	pre-b cell enhancing factor precursor		NAPRTase	0.6405
5primeCluster0456.Contig1		gi|37638284|gb|CF741945.1|CF741945	0	162	---NA---		Pfam-B_11765, Pfam-B_14559	1.9314
5primeCluster0461.Contig1		gi|22679525|gb|BU165573.1|BU165573	1.00E-33	143	fk506-binding protein 4	F:GO:0005488	Pfam-B_2967, Pfam-B_6046, Pfam-B_3120, TPR_1, TPR_2	0.21562

5primeCluster0467.Contig1		gi|13135773|gb|BG329335.1|BG329335	3.00E-22	82.2	cs042_humaname: full=upf0608 protein c19orf42 flags: precursor			
5primeCluster0472.Contig1		gi|3396334|gb|AI070083.1|AI070083	0	210	ribosomal protein l27	C:GO:0030529	Ribosomal_L27e, KOW	
5primeCluster0472.Contig2	**Embryo specific expression**	gi|62854559|gb|DN884604.1|DN884604	0	231	ribosomal protein l27	C:GO:0030529	Ribosomal_L27e, KOW	
5primeCluster0485.Contig1		gi|50140585|gb|CO472446.1|CO472446	2.00E-19	90.9	heat shock protein 90	P:GO:0006457,F:GO:0051082,C:GO:0005737,F:GO:0005524,P:GO:0006950	HSP90	0.87799
5primeCluster0495.Contig1		gi|20360391|gb|BQ184840.1|BQ184840	0.007	44.1				
5primeCluster0496.Contig1		gi|50138538|gb|CO471508.1|CO471508	0	185	brain protein 44-like protein		UPF0041	0.3574
5primeCluster0501.Contig1		gi|62854198|gb|DN884243.1|DN884243	4.00E-18	91.8	ubiquinol-cytochrome c reductase complex		UCR_UQCRX_QCR9	
5primeCluster0507.Contig1	**Embryo specific expression**	gi|22812700|gb|BU506467.1|BU506467	2.00E-09	62.9	family with sequence similaritymember a	F:GO:0003674,P:GO:0008150		
5primeCluster0510.Contig1		gi|62879604|gb|DN894841.1|DN894841	1.00E-15	83.5	ns1 binding protein		Kelch_1, Pfam-B_6141	
5primeCluster0514.Contig1		gi|62851579|gb|DN881624.1|DN881624	1.00E-36	153	ubiquinol-cytochrome c reductase binding protein	C:GO:0016020,C:GO:0005739	UCR_14kD	0.31422
5primeCluster0515.Contig1		gi|24731375|gb|CA395682.1|CA395682	9.00E-36	93.6	ubiquitin-fold modifier 1		Ufm1	
5primeCluster0515.Contig2		gi|24731375|gb|CA395682.1|CA395682	3.00E-40	165	ubiquitin-fold modifier 1		Ufm1	

5primeCluster0516.Contig2		BY798344	3.00E-37	149	small nuclear ribonucleoprotein polypeptide g	C:GO:0005732,F:GO:0031202,C:GO:0005681,F:GO:0005515,C:GO:0030532,P:GO:0000245	LSM	0.08485
5primeCluster0521.Contig1		gi|9593874|gb|BB538374.1|BB538374	0.0004	48.1				
5primeCluster0522.Contig1	Embryo specific expression	gi|49673830|gb|CO427536.1|CO427536	4.00E-38	134	phosphoglycerate mutase family protein		PGAM	1.62139
5primeCluster0523.Contig1		gi|20359663|gb|BQ184112.1|BQ184112	0.006	44.1				
5primeCluster0531.Contig1		gi|91743724|gb|EB380379.1|EB380379	5.00E-23	107	abhydrolase domain containingisoform cra_c			
5primeCluster0550.Contig1		gi|49672810|gb|CO426516.1|CO426516	2.00E-28	65.9	fimbrin plastin			1.39176
5primeCluster0553.Contig1		gi|85176586|gb|DW597899.1|DW597899	1.00E-20	87.7	map2k6 protein	F:GO:0016301		
5primeCluster0558.Contig1		gi|62039933|gb|DN688542.1|DN688542	2.00E-40	165	eno_lolpeame: full=enolaseame: full=2-phosphoglycerate dehydrataseame: full=2-phospho-d-glycerate hydro-lyase	C:GO:0000015,F:GO:0004634,F:GO:0000287,P:GO:0006096		1.1131
5primeCluster0561.Contig1		gi|13103565|gb|BG306038.1|BG306038	3.00E-14	78.5	proteasome subunit alpha type-3			
5primeCluster0592.Contig1		gi|19021562|gb|BM708304.1|BM708304	0.006	25.8				

5primeCluster0596.Contig1	gi|62049228|gb|DN693138.1|DN693138	0.0001	46.4	calcium binding atopy-related autoantigen 1 isoform 1			
5primeCluster0598.Contig1	gi|13268617|gb|BG372080.1|BG372080	4.00E-23	98.7	heat shock protein 70	F:GO:0005524,P:GO:0006950,F:GO:0000166		1.36164
5primeCluster0602.Contig1	gi|14060917|gb|BG750264.1|BG750264	3.00E-16	84.5	cysteine-rich with egf-like domains 1	F:GO:0005509,C:GO:0005783,C:GO:0005576		
5primeCluster0609.Contig1	gi|119789801|gb|EH282359.1|EH282359	1.00E-10	65.7	cytochrome csubunit	F:GO:0004129,P:GO:0006118		
5primeCluster0610.Contig1	GS013952	0.008	26.7				
5primeCluster0621.Contig1	BY795030	7.00E-19	87.2	ribosomal protein rpl39	C:GO:0005840,F:GO:0003735,P:GO:0006412		0.09322
5primeCluster0621.Contig2	BY795030	2.00E-21	95.9	ribosomal protein rpl39			1.63097
5primeCluster0627.Contig1	BY800472	0.003	38.2				
5primeCluster0630.Contig1	GS004626	0.003	32.2				
Ika-1224-No22_B03_002	GS020645	0.005	34.2				
Ika-1224-No22_B05_002	gi|20363398|gb|BQ187847.1|BQ187847	1.00E-05	47.6	fyn binding protein (fyb-120 130)	P:GO:0006468,P:GO:0007243,P:GO:0045576,F:GO:0005102,P:GO:0006607,C:GO:0005634,P:GO:0006955,C:GO:001562	SH3_2	
Ika-1224-No22_C12_003	gi|15709914|gb|BI732901.1|BI732901	0.008	44.1				
Ika-1224-No22_D12_004	BY797664	0.006	38.2				
Ika-1224-No22_F04_002	BY797751	0.002	38.2				

Ika-1224-No22_H01_004		GS004743	0.008	27.6				
ika-1224-No9_A09_001	**Embryo specific expression**	gi|37600690|gb|CF726522.1|CF726522	0	186	dynein light intermediate chain	F:GO:0003777,F:GO:0042623,P:GO:0007052,F:GO:0032555,P:GO:0046605,C:GO:0005868,P:GO:0007018,F:GO:0005515,P:GO:0048813	DLIC, Pfam-B_8060	1.0469
ika-1224-No9_B03_002		gi|14061917|gb|BG751264.1|BG751264	0.008	35				
ika-1224-No9_D03_004		gi|24559278|gb|CA341182.1|CA341182	0	219	ribosomal protein l26	P:GO:0000022,P:GO:0006412,P:GO:0040010,F:GO:0003723,P:GO:0009792,C:GO:0005739,F:GO:0003735,P:GO:0000003,P:GO:0042254,P:GO:0002119,F:GO:0005515,N:GO:0022625	KOW	
ika-1224-No9_D09_004		gi|18980734|gb|BM670843.1|BM670843	0.004	44.1				
ika-1224-No9_D12_004		BY799310	0.01	38.2				
ika-1224-No9_E02_001		BY797751	0.005	35.4				
ika-1224-No9_G11_003		gi|37599375|gb|CF725207.1|CF725207	1.00E-05	50.6	ccr4-not transcriptionsubunit 3		Not3	
ika1119-No8_A02_001		gi|9617433|gb|BB546005.1|BB546005	0.002	46.1				

ika1119-No8_A06_001	gi|13298901|gb|BG374429.1|BG374429	0.004	44.1
ika1119-No8_B08_002	BY798528	0.0006	42.1
ika1119-No8_D11_004	BY796060	0.008	36.2

ika1119-No8_E03_001	gi|54439388|gb|CV555651.1|CV555651	1.00E-38	160	myosin heavy chain	P:GO:0048547,P:GO:0030048,C:GO:0043005,P:GO:0055013,P:GO:0030010,P:GO:0035295,P:GO:0007507,P:GO:0051321,C:GO:0030017,P:GO:0030239,P:GO:0016044,P:GO:0022402,P:GO:0016477,P:GO:0044237,P:GO:0050793,P:GO:0000226,P:GO:0019538,P:GO:0000904,F:GO:0032559,C:GO:0044459,P:GO:0031175,P:GO:0021591,P:GO:0051656,F:GO:0042623,P:GO:0006936,P:GO:0000910,P:GO:0048729,P:GO:0000003,P:GO:0050794,P:GO:0002376,C:GO:0005859,P:GO:0065008,P:GO:0007155,P:GO:0030855,F:GO:0003774,C:GO:0044448,P:GO:0002164,P:GO:0002165,P:GO:0007391,P:GO:0006950,P:GO:	Myosin_tail_1	1.18944

ika1119-No8_F05_002		BY795248	0.008	33.6				
ika1119-No8_F07_002		GS019919	0.001	36.2				
ika1119_A04_001		GS004815	0.007	32.2				
Ika1124-No28_C02_003		BY800302	0.001	40.1				
ika1124-No2_B09_002	**Positive selection**	gi|85169667|gb|DW595248.1|DW595248	0	361	protein	P:GO:0002119,F:GO:0005488,C:GO:0046540,C:GO:0005681,P:GO:0000003,P:GO:0009792,C:GO:0016021,P:GO:0040007,C:GO:0005829,P:GO:0000398	PRP38	4.30403
ika1124-No30_G07_003		BY795637	0.001	40.1				
ika1125-No7_A04_001		BY798345	0.009	38.2				
ika1224-No10_A05_001		gi|9125360|gb|BE254927.1|BE254927	0.009	42.1				
ika1224-No10_A09_001		GS014141	0.001	36.2				
ika1224-No10_B09_002		BY800108	0.007	34.8				
ika1224-No10_E05_001	**Embryo specific expression**	gi|16450468|gb|BB600534.2|BB600534	5.00E-39	81.7	cxxc finger 6	F:GO:0046872,F:GO:0003674,P:GO:0008150,F:GO:0008270,F:GO:0003677,C:GO:0005634		1.25013
ika1224-No11_A04_001		gi|62826716|gb|DN857067.1|DN857067	0.003	42.3	---NA---			

ika1224-No11_C10_003	gi|18995135|gb|BM685239.1|BM685239	0.008	44.1				
ika1224-No11_D12_004	GS013792	0.005	34.2				
ika1224-No11_F04_002	BY795080	0.01	38.2				
Ika1224-No12_D09_004	gi|40318733|gb|CK352846.1|CK352846	3.00E-25	87.6	transcription initiation factor tfiid	C:GO:0000124,C:GO:0046695,P:GO:0006367,C:GO:0005730,P:GO:0045944,F:GO:0016251,F:GO:0046983,F:GO:0003700,F:GO:0004402,P:GO:0016573,C:GO:0005669	TFIID_90kDa	0.76572
Ika1224-No12_E07_001	BY798380	0.01	36.2				
Ika1224-No12_E08_001	BY796056	0.002	38.2				
ika1224-No13_D05_004	gi|16062248|gb|BB709079.1|BB709079	0.008	44.1				
ika1224-No13_D07_004	BY800118	0.0006	42.1				
ika1224-No13_E04_001	BY797696	0.01	38.2				
ika1224-No13_F06_002	BY795820	0.009	38.2				
ika1224-No13_H04_004	gi|19044026|gb|BM723028.1|BM723028	1.00E-06	49.6	set domain and mariner transposase fusion			
Ika1224-No14_C05_003	gi|39890957|gb|CK301009.1|CK301009	0.0004	43.7	---NA---			

Ika1224-No14_E08_001		GS014043	0.004	34.2			
Ika1224-No14_F06_002		gi|42746304|gb|CK780626.1|CK780626	2.00E-12	73.5	ubiquitin specific peptidase 3	P:GO:0009987,F:GO:0008234	UCH, zf-UBP
Ika1224-No14_G09_003	Embryo specific expression	gi|46465385|gb|CN459659.1|CN459659	0.009	44.1	hypothetical loc100131203		
Ika1224-No14_G10_003		gi|15714351|gb|BI737338.1|BI737338	0.0001	47.3			
Ika1224-No15_A02_001		GS020766	0.004	34.2			
Ika1224-No15_B12_002		gi|3399133|gb|AI072939.1|AI072939	0.009	44.1			
Ika1224-No15_C08_003		gi|24731064|gb|CA395520.1|CA395520	0.003	44.1			
Ika1224-No15_E05_001		BY797989	0.009	36.2			PB1

Ika1224-No15_G05_003		gi|62826682|gb|DN857033.1|DN857033	2.00E-10	66.1	sequestosome 1	F:GO:0019901,P:GO:0016197,P:GO:0006511,C:GO:0005770,C:GO:0005634,P:GO:0030154,P:GO:0043122,P:GO:0006915,F:GO:0046872,F:GO:0042169,F:GO:0030971,P:GO:0051291,P:GO:0008150,P:GO:0045944,P:GO:0007242,P:GO:0006955,F:GO:0005080,C:GO:0005768,F:GO:0043130,C:GO:0005737,P:GO:0006950,P:GO:0008104,C:GO:0005829,F:GO:0008270,F:GO:0005515,C:GO:0005575		
Ika1224-No16_D01_004		GS020666	0.005	34.2				
Ika1224-No16_D10_004		BY800091	0.003	40.1				
Ika1224-No16_F06_002		gi|46816516|gb|CN503892.1|CN503892	0.004	44.1				
Ika1224-No16_G02_003	Embryo specific expression	gi|22667692|gb|BU154160.1|BU154160	0	208	protein	F:GO:0005097,C:GO:0005625,F:GO:0017137,P:GO:0043087	Pfam-B_598	0.9222
Ika1224-No16_G07_003		gi|19024339|gb|BM711081.1|BM711081	1.00E-09	53.3	flj00022 protein	F:GO:0003674,C:GO:0005737,C:GO:0005575	Pfam-B_9625, DUF2464	0.47633

Ika1224-No17_F03_002		BY794969	0.005	36.3
Ika1224-No17_H04_004		GS004729	0.001	36.2
Ika1224-No18_B11_002		gi|75833579|gb|DT863839.1|DT863839	0.002	46.1
Ika1224-No18_F06_002	**Embryo specific expression**	gi|23672012|gb|BU737013.1|BU737013	0.0001	44.1

ika1224-No19_E06_001	gi|75851466|gb|DT881726.1|DT881726	5.00E-43	174	member ras oncogene family	P:GO:0030100,P:GO:0022416,C:GO:0030140,C:GO:0009536,C:GO:0005802,P:GO:0008103,P:GO:0016360,C:GO:0033116,P:GO:0006913,P:GO:0007269,F:GO:0008565,P:GO:0007349,C:GO:0005624,P:GO:0042052,P:GO:0006355,C:GO:0005579,P:GO:0040011,C:GO:0042470,F:GO:0005525,C:GO:0005764,P:GO:0007317,P:GO:0006887,C:GO:0042175,C:GO:0005829,C:GO:0005770,P:GO:0006928,C:GO:0005741,P:GO:0046887,P:GO:0007264,C:GO:0005813,F:GO:0003677,C:GO:0005795,P:GO:0050807,C:GO:0005769,P:GO:0006888,P:GO:0019730,C:GO:0048471,C:GO:0005811,P:GO:0006895,F:GO:0003924,P:GO:	Ras	1.17698

ika1224-No19_E09_001	gi|62876140|gb|DN891377.1|DN891377	8.00E-05	44.1			zf-C2H2	
ika1224-No19_F11_002	GS020725	0.005	34.2				
ika1224-No19_G03_003	gi|9129088|gb|BE258596.1|BE258596	0.008	42.1				
ika1224-No19_G04_003	gi|62878866|gb|DN894103.1|DN894103	0.008	44.1				
ika1224-No19_G05_003	gi|18989049|gb|BM679153.1|BM679153	6.00E-34	144	50s ribosomal protein l22	C:GO:0005762,F:GO:0003735,P:GO:0009792,P:GO:0006412	Ribosomal_L22	0.20137

Ika1224-No20_A07_001	gi|22369065|gb|BQ953587.1|BQ953587	3.00E-33	141	adp-ribosylation factor	P:GO:0016358,F:GO:0005154,C:GO:0005802,F:GO:0005057,P:GO:0009792,P:GO:0008360,P:GO:0006891,P:GO:0007155,P:GO:0006893,P:GO:0030866,P:GO:0040010,P:GO:0007269,F:GO:0008565,C:GO:0005624,F:GO:0003956,P:GO:0040011,C:GO:0005935,P:GO:0007030,F:GO:0005525,P:GO:0044408,P:GO:0031529,P:GO:0007173,F:GO:0016004,P:GO:0018996,P:GO:0006886,C:GO:0005829,P:GO:0006471,P:GO:0007520,P:GO:0006928,C:GO:0001726,C:GO:0005934,P:GO:0016197,P:GO:0006915,P:GO:0002119,P:GO:0006888,P:GO:0048261,P:GO:0000723,C:GO:0005768,P:GO:0035020,P:GO:0007276,P:GO:	Arf

Ika1224-No20_B05_002	gi|62846056|gb|DN876115.1|DN876115	0.0006	48.1					
Ika1224-No20_C02_003	gi|19046236|gb|BM724905.1|BM724905	5.00E-40	147	cdc37	C:GO:0005829,P:GO:0000910,F:GO:0051879,F:GO:0051087,P:GO:0000003,P:GO:0007169,P:GO:0006605,F:GO:0030296,F:GO:0000166,F:GO:0051082,P:GO:0007275,P:GO:0009653,P:GO:0000079	CDC37_M	0.20548	
Ika1224-No20_C07_003	GS004636	0.008	32.2					
Ika1224-No20_H11_004	gi|40679977|gb|CK431159.1|CK431159	6.00E-08	41.9	hypoxia associated factor	P:GO:0006397,C:GO:0005634,P:GO:0008380,C:GO:0005737,F:GO:0003674,P:GO:0008629,C:GO:0005575,C:GO:0005681,C:GO:0005829,P:GO:0007050,P:GO:0045585	SART-1		
Ika1224-No21_B06_002	GS004683	0.005	34.2					
Ika1224-No21_B07_002	gi|16401319|gb|BB281433.2|BB281433	0.009	44.1					
Ika1224-No21_B09_002	BY799853	6.00E-06	42.8	myosin iiia	F:GO:0004672,F:GO:0000166			
Ika1224-No21_D10_004	GS013988	0.01	32.2					

Ika1224-No21_E12_001		gi|18979080|gb|BM669183.1|BM669183	0.008	44.1				
Ika1224-No21_F06_002		gi|62881247|gb|DN896484.1|DN896484	1.00E-30	133	multicopper oxidase	F:GO:0005507,P:GO:0006825,P:GO:0007584,F:GO:0004322,C:GO:0005615,P:GO:0030218,P:GO:0030005,F:GO:0005515,P:GO:0007568,C:GO:0046658,P:GO:0007596	Cu-oxidase_2	1.1544
Ika1224-No21_H04_004	**Embryo specific expression**	gi|57590282|gb|CX563253.1|CX563253	0.009	44.1				
Ika1224-No23_B07_002		GS004703	0.002	34.2				
Ika1224-No23_C08_003		gi|62830113|gb|DN860464.1|DN860464	0.004	40				
Ika1224-No23_D01_004	**Embryo specific expression**	GS004674	0.003	29.9				
Ika1224-No23_G04_003	**Embryo specific expression**	gi|77506621|gb|DV216629.1|DV216629	0	187	spermidine synthase	C:GO:0005737,P:GO:0006597,P:GO:0008295,P:GO:0009926,F:GO:0004766,P:GO:0015940,P:GO:0010087,F:GO:0016768,C:GO:0005634,N:GO:0010487	Spermine_synth	
Ika1224-No24_A03_001		BY799341	0.007	36.2				

Ika1224-No24_A06_001	Embryo specific expression	gi|16780503|gb|BM051236.1|BM051236	3.00E-16	75.3	o-methyltransferase	P:GO:0044238,F:GO:0008171,P:GO:0009058,F:GO:0008757,C:GO:0044464,P:GO:0044237	Methyltransf_3
Ika1224-No24_B08_002		GS020505	0.004	34.2			
Ika1224-No24_C12_003		gi|32175750|gb|CD674019.1|CD674019	2.00E-19	71.6	abc transporter	P:GO:0033344,C:GO:0000139,C:GO:0043190,F:GO:0015485,F:GO:0005548,F:GO:0008565,P:GO:0009720,P:GO:0010033,P:GO:0045542,C:GO:0005615,F:GO:0043531,P:GO:0043691,P:GO:0015031,C:GO:0005789,F:GO:0005543,P:GO:0006911,P:GO:0055091,F:GO:0046982,N:GO:0034041,C:GO:0005768,P:GO:0045449,P:GO:0032367,P:GO:0033700,P:GO:0042987,F:GO:0042803,F:GO:0019534,C:GO:0005887,F:GO:0005524,F:GO:0017127,C:GO:0009898,P:GO:0042632	DUF258, Pfam-B_5069
Ika1224-No24_D04_004		BY796668	0.004	38.2			

Ika1224-No24_D06_004	gi|18528421|gb|BM479379.1|BM479379	0.0005	48.1			
Ika1224-No24_D08_004	gi|62832186|gb|DN862537.1|DN862537	0	237	actin-related protein 2 3 complex subunit 4	C:GO:0005885,P:GO:0040010,F:GO:0030674,P:GO:0000147,P:GO:0009792,P:GO:0007015,P:GO:0008360,F:GO:0051015,P:GO:0030041,P:GO:0006911,C:GO:0042995,P:GO:0030031,F:GO:0005200,P:GO:0045010,C:GO:0005737	ARPC4
Ika1224-No24_E05_001	GS004645	0.004	34.2			
Ika1224-No24_F05_002	GS013936	0.005	34.2			
Ika1224-No24_F07_002	GS013783	0.001	36.2			
Ika1224-No24_F11_002	BY798838	0.009	38.2			
Ika1224-No24_G01_003	gi|21174067|gb|BQ434991.1|BQ434991	8.00E-11	67.5	heterogeneous nuclear ribonucleoprotein u-like 1	P:GO:0006915,F:GO:0003677,F:GO:0003723,C:GO:0005634,P:GO:0006355,F:GO:0000166	ASP
Ika1224-No24_H08_004	GS014020	0.001	34.2			

Ika1224-No25_D03_004	gi|22701704|gb|BU187720.1|BU187720	0	184	kinesin heavy chain	C:GO:0043005,F:GO:0008574,P:GO:0040010,P:GO:0007269,C:GO:0035253,C:GO:0005624,P:GO:0040011,P:GO:0007317,C:GO:0005874,P:GO:0048311,P:GO:0007303,P:GO:0047496,F:GO:0008017,P:GO:0019227,P:GO:0002009,P:GO:0008045,P:GO:0008088,P:GO:0047497,P:GO:0007310,P:GO:0010171,C:GO:0048471,C:GO:0005871,F:GO:0005524,P:GO:0006839,P:GO:0001754,C:GO:0005739	ADIP, Microtub_assoc, H00K, Pfam-B_7610, Cenp-F_leu_zip	1.30177
Ika1224-No25_D05_004	BY797834	0.01	38.2				
Ika1224-No25_G02_003	gi|85207344|gb|DW608201.1|DW608201	8.00E-06	54				
Ika1224-No25_G04_003	gi|13105897|gb|BG308370.1|BG308370	8.00E-05	50.1				
Ika1224-No25_G08_003	GS020701	0.004	34.2				

Ika1224-No26_B09_002	gi|19389351|gb|BM930178.1|BM930178	0.0005	48.1	exosome component 10	C:GO:0000176,P:GO:0006396,C:GO:0005634,F:GO:0016787,P:GO:0006364,F:GO:0003676,F:GO:0003723,F:GO:0004527,C:GO:0005730,P:GO:0006139,C:GO:0005622,P:GO:0000184,F:GO:0004518,C:GO:0000178,F:GO:0008408
Ika1224-No26_D12_004	gi|23675980|gb|BU738643.1|BU738643	0.002	46.1		
Ika1224-No26_E05_001	gi|19393251|gb|BM934099.1|BM934099	6.00E-05	47.8		
Ika1224-No26_G11_003	gi|62873057|gb|DN888294.1|DN888294	0.008	44.1		
Ika1224-No26_G12_003	gi|62856864|gb|DN886909.1|DN886909	0.009	44.1		
Ika1224-No27_A02_001	BY800039	0.003	40.1		

Ika1224-No27_B03_002	gi|22811032|gb|BU504799.1|BU504799	9.00E-15	80.8	insulin-degrading enzyme	F:GO:0031626,P:GO:0007548,P:GO:0051291,F:GO:0008270,C:GO:0005625,F:GO:0004871,C:GO:0031597,P:GO:0043171,F:GO:0043559,C:GO:0005615,P:GO:0051603,C:GO:0009986,P:GO:0045861,P:GO:0042447,F:GO:0005524,P:GO:0050435,F:GO:0016887,P:GO:0007165,F:GO:0004231,P:GO:0051289,F:GO:0042803,C:GO:0005782,C:GO:0005634	Peptidase_M16
Ika1224-No27_B08_002	gi|19396614|gb|BM937462.1|BM937462	1.00E-07	55.1			
Ika1224-No27_C07_003	GS004891	0.003	34.2			
Ika1224-No27_D11_004	gi|14977218|gb|BI300938.1|BI300938	4.00E-06	51.9	---NA---		
Ika1224-No27_G04_003	GS020571	0.001	36.2			

Ika1224-No27_G06_003		gi|62021563|gb|DN679604.1|DN679604	0	234	vacuolar atp synthase subunit h	F:GO:0046961,P:GO:0009792,F:GO:0005515,F:GO:0008553,P:GO:0040007,F:GO:0046933,P:GO:0015991,P:GO:0040011,P:GO:0040002,P:GO:0007035,P:GO:0018996,P:GO:0008340,F:GO:0001671,P:GO:0015986,P:GO:0006897,P:GO:0002119,C:GO:0000221,C:GO:0000300,F:GO:0005524	V-ATPase_H_N, V-ATPase_H_C
Ika1224-No27_H05_004		gi|46820176|gb|CN507552.1|CN507552	0.007	44.1			
Ika1224-No27_H10_004		GS008123	0.001	34.2			
ika1224-No4_E08_001	**Embryo specific expression**	GS008153	0.004	31.3			
ika1224-No4_G10_003		gi|62837041|gb|DN867100.1|DN867100	0.003	42.8			
ika1224_C06_003		gi|32180633|gb|CD678902.1|CD678902	0	306			

ikaD2-2_B11_002	**Positive selection**	GS020581	0.003	31.3	thermosome	F:GO:0008565,P:GO:0009792,C:GO:0005856,P:GO:0006457,P:GO:0007010,P:GO:0040007,C:GO:0005832,P:GO:0040035,P:GO:0007283,F:GO:0005524,P:GO:0002119,P:GO:0044408,P:GO:0040011,F:GO:0051082,P:GO:0051131,P:GO:0008340	Cpn60_TCP1	3.0724
ikaD2-6_B10_002		GS020581	0.003	31.3				
ika_c11		BY799535	0.01	35.4				
ika_c24		gi|58031468|gb|CX729011.1|CX729011	0.008	44.1				
ika_c29		BY799800	0.0007	42.1				
ika_c36		BY797277	2.00E-11	64.8				
ika_c37		gi|15706568|gb|BI729555.1|BI729555	3.00E-05	52	cg11148-isoform a isoform 1		Pfam-B_18091	
ika_c61		GS013929	0.004	34.2				
ika_c67		gi|83161863|gb|DB384234.1|DB384234	1.00E-05	49.2	tnf receptor-associated protein 1			

ika_c70	**Embryo specific expression**	gi|62040790|gb|DN688965.1|DN688965	0	131	protein	P:GO:0030100,C:GO:0030122,P:GO:0000003,C:GO:0009536,C:GO:0005802,P:GO:0009792,P:GO:0006726,P:GO:0008286,P:GO:0040007,P:GO:0006898,P:GO:0006461,P:GO:0007269,C:GO:0030123,F:GO:0008565,P:GO:0016183,P:GO:0040011,P:GO:0006886,F:GO:0030276,P:GO:0048268,P:GO:0048072,P:GO:0002119,P:GO:0006896,C:GO:0008021,P:GO:0007040,C:GO:0030121,P:GO:0010171,C:GO:0005739	Clat_adaptor_s	0.1915

ika_c8	Embryo specific expression, Positive selection	gi|22695458|gb|BU181474.1|BU181474	0	252	high density lipoprotein binding protein	F:GO:0005515,P:GO:0007275,P:GO:0051276,P:GO:0048518,P:GO:0006323,C:GO:0044427,P:GO:0044237,P:GO:0040011,P:GO:0051179,P:GO:0048856,P:GO:0006629,F:GO:0003677,C:GO:0012505,C:GO:0044444,C:GO:0005634	KH_1, Pfam-B_12719	2.39913
OctEye_0033F_034		gi|13059618|gb|BG296702.1|BG296702	0.007	44.1				
OctEye_0148F_059		BY795189	9.00E-07	45.1	ribosomal protein l34		Ribosomal_L34e	
OctEye_0206F_016		gi|62842158|gb|DN872217.1|DN872217	3.00E-07	54.2	briggsae cbr-aat-1 protein	C:GO:0005886		
OctEye_0307F_019		gi|62889367|gb|DN904604.1|DN904604	0.005	44.1				
OctEye_0317F_024		GS013987	0.001	36.2				
OctEye_0487F_005		gi|62878532|gb|DN893769.1|DN893769	0.0001	50.1				
OctEye_0591F_017		gi|62023994|gb|DN677464.1|DN677464	0.005	41.8				
OctEye_0659F_083		gi|75838361|gb|DT868621.1|DT868621	0.004	38.6				
OctEye_0745F_070		gi|75835824|gb|DT866084.1|DT866084	1.00E-07	57.1	isoform a			

OctEye_0789F_020	gi|32173308|gb|CD671568.1|CD671568	1.00E-07	53.3	coiled-coil domain-containing protein 94		DUF572	
OctEye_0826F_062	BY798607	0.0006	38.6			DUF2154	
OctEye_0912F_057	gi|77501382|gb|DV213912.1|DV213912	1.00E-25	116	---NA---		NDUF_B7	1.72221
OctEye_1096F_045	GS020542	0.008	30.2				
OctEye_1119F_065	GS020677	0.009	32.2				
OctEye_1134F_080	gi|58032450|gb|CX729993.1|CX729993	0.007	44.1				
OctEye_1164F_015	BY796496	0.009	38.2				
OctEye_1182F_032	gi|62844591|gb|DN874650.1|DN874650	0.002	46.1				
OctEye_1263F_017	gi|62828798|gb|DN859149.1|DN859149	4.00E-28	124	cytochrome c oxidase subunit via polypeptide 1			
OctEye_1302F_060	gi|62038659|gb|DN687898.1|DN687898	0.002	41.4	zgc:55406 protein	F:GO:0004568,F:GO:0043169,F:GO:0008061,P:GO:0005975,C:GO:0005576,F:GO:0003824,F:GO:0004553,P:GO:0006032,P:GO:0006030		
OctEye_1343F_097	gi|62038852|gb|DN687996.1|DN687996	3.00E-06	46.9	collagen type vi alpha 4			
OctEye_1353F_006	gi|62828989|gb|DN859340.1|DN859340	5.00E-08	54.7	---NA---			

OctEye_1499F_055	gi|85188936|gb|DW601763.1|DW601763	0	191	mitochondrial trifunctionalbeta subunit	F:GO:0003988,P:GO:0008152	0.5225
OctEye_1534F_096	gi|58031885|gb|CX729428.1|CX729428	1.00E-06	51.9	mgc89819 protein		
OctEye_1560F_029	gi|32178782|gb|CD677051.1|CD677051	7.00E-23	107	atph+mitochondrial f1o subunit	F:GO:0015078	1.57288
OctEye_1579F_039	BY798633	0.004	38.2			
OctEye_1586F_058	gi|18973569|gb|BM666120.1|BM666120	0.01	37.3			
OctEye_1592F_061	BY796454	0.002	34.1			
OctEye_1748F_027	gi|40319367|gb|CK353480.1|CK353480	8.97E-44	177	homolog precursor		0.1216
OctEye_1812F_091	GS014021	0.0006	30.9			
OctEye_1825F_002	gi|24559221|gb|CA341125.1|CA341125	1.00E-09	65.9	arge subunit family member (rpl-)		
OctEye_1832F_013	gi|62031964|gb|DN684517.1|DN684517	0.007	42.1			
OctEye_2031F_017	GS008102	0.0002	38.2			
OctEye_2085F_068	BY794980	5.00E-16	75.8	ribosomal protein s17		
OctEye_2126F_016	gi|6516364|gb|AW210424.1|AW210424	0.008	30.4			
OctEye_2221F_008	gi|24734171|gb|CA397140.1|CA397140	0.0004	48.1			

OctEye_2262F_060		gi|119785924|gb|EH279915.1|EH279915	4.00E-14	78.5	selenoprotein1	F:GO:0008430,C:GO:0005739,C:GO:0005886,F:GO:0003954	0.91171
OctEye_2384F_089		GS020757	0.008	30.2			
OctEye_2498F_010		BY799605	0.004	36.2			
OctEye_2597F_004		gi|58178287|gb|CX767934.1|CX767934	0	254	atp synthase	C:GO:0045261,F:GO:0046933,C:GO:0005743,P:GO:0015986,F:GO:0046872,F:GO:0005524,F:GO:0005515,F:GO:0046961	
OctEye_2648F_061		GS019927	0.009	32.2			
OctEye_2674F_090	**Positive selection**	gi|58035934|gb|CX733460.1|CX733460	4.00E-34	144	coatomer proteinsubunit alpha	P:GO:0006461,F:GO:0005515,F:GO:0008565,P:GO:0016192,P:GO:0006886,C:GO:0030126,F:GO:0005198	6.37134
OctEye_2694F_012		GS020566	0.003	32.2			
OctEye_2707F_019		GS020537	0.009	32.2			
OctEye_2728F_045		gi|119790103|gb|EH282661.1|EH282661	0.0001	43.7	antioxidant enzyme	F:GO:0046872,P:GO:0030001	
OctEye_2790F_012		gi|46816584|gb|CN503960.1|CN503960	2.00E-11	69.9	actin 5c		
OctEye_2879F_097		gi|37634583|gb|CF738246.1|CF738246	3.00E-34	145	u2small nuclear rna auxiliary factor 1	F:GO:0003723,C:GO:0005634,F:GO:0000166,F:GO:0008270	0.31488
OctEye_2993F_019		gi|58060940|gb|CX734104.1|CX734104	2.00E-19	96.6	---NA---		

OctEye_3104F_032		gi|5209870|gb|AI763935.1|AI763935	7.00E-42	170	nadh dehydrogenase	C:GO:0016020,C:GO:0005739		0.95984
OctEye_3179F_006		gi|75834465|gb|DT864725.1|DT864725	1.00E-21	57.4	translationally controlled tumor protein	F:GO:0005262,F:GO:0005509,P:GO:0006816,F:GO:0019855		
OctEye_3268F_012		gi|119779499|gb|EH278533.1|EH278533	0.0002	42.8				
OctEye_3507F_052		gi|58033286|gb|CX730812.1|CX730812	0.01	26.7				
OctEye_3723F_072		gi|126522970|gb|EL646689.1|EL646689	8.00E-33	139	af486842_160s ribosomal protein l37	F:GO:0046872,F:GO:0003723,C:GO:0030529		0.92411
OctEye_3881F_039		gi|46825733|gb|CN513109.1|CN513109	0	221	delta-aminolevulinic acid dehydratase	F:GO:0046872,F:GO:0004655,P:GO:0006779		
OctEye_3919F_082	**Positive selection**	gi|32464719|gb|CD805893.1|CD805893	0	308	centaurin gamma			2.48762
OctEye_3974F_045		gi|50135821|gb|CO470232.1|CO470232	6.00E-40	163	glyceraldehyde-3-phosphate dehydrogenase-2	F:GO:0051287,P:GO:0006096,F:GO:0004365		1.2506
OctEye_4022F_093	**Embryo specific expression**	BY796351	0.009	26.7				
OctEye_4025F_087		GS020566	0.004	34.2				
OctEye_4048F_026		BY795001	5.00E-05	42.1				
OctEye_4133F_005		gi|40319296|gb|CK353409.1|CK353409	0.002	28.6				
OctEye_4228F_012		gi|19017266|gb|BM704008.1|BM704008	8.00E-23	105	mgc84219 protein	C:GO:0005737,F:GO:0003779,C:GO:0030054,C:GO:0045202,F:GO:0004864	RPEL	0.82442

OctEye_4486F_077		gi|62882487|gb|DN897724.1|DN897724	9.00E-11	53.8	cystatin b	F:GO:0004866,C:GO:0005622	Cystatin	
OctEye_4502F_093	**Embryo specific expression**	gi|126522032|gb|EL645883.1|EL645883	6.00E-06	52				
OctEye_4528F_026		BY795027	0.009	38.2				
OctEye_4530F_027		gi|5210219|gb|AI764284.1|AI764284	0.002	46.1			Coatomer_E	
OctEye_4536F_030		gi|41347505|gb|CK626619.1|CK626619	3.00E-15	80.3	epsilon subunit of coatomer protein complex isoform a	P:GO:0016192,F:GO:0005488,C:GO:0031410,P:GO:0016043,C:GO:0044425,C:GO:0031090,C:GO:0012505,C:GO:0044431		0.1668
OctEye_4576F_074	**GO annotation**	gi|40549930|gb|CK396398.1|CK396398	0	182	lim and sh3 domain protein		Nebulin, LIM	1.56255
OctEye_4608F_102		gi|85192029|gb|DW602650.1|DW602650	0.005	42.1				
OctEye_4725F_021		gi|46822989|gb|CN510365.1|CN510365	3.99E-43	175	u424_dropsame: full=upf0424 protein ga19395	F:GO:0003674,P:GO:0008150,C:GO:0005575	PITH	0.46073
OctEye_4771F_068		gi|37631811|gb|CF735475.1|CF735475	2.00E-24	109	hyperplastic discs cg9484-pa isoform 1		HECT	
OctEye_4817F_018		GS014020	0.004	32.2				
OctEye_4872F_077		gi|18501048|gb|BM452008.1|BM452008	1.00E-14	79.8				
OctEye_4900F_011		gi|46566151|gb|CN484647.1|CN484647	4.00E-12	73.8				

OctEye_4909F_008	gi|62873918|gb|DN889155.1|DN889155	0	178	---NA---	F:GO:0042578,P:GO:0005975		1.43801
OctFet_0406F_028	gi|85169445|gb|DW595187.1|DW595187	3.00E-05	46.9	cat eye syndrome candidate region 5 protein	P:GO:0008152,F:GO:0016787		
OctFet_0464F_089	gi|46815207|gb|CN502583.1|CN502583	6.00E-12	71.2	mitochondrial atp synthase f chain		WRW	1.26487
OctFet_0712F_045	gi|37635937|gb|CF739599.1|CF739599	3.00E-40	126	cytochrome c oxidase subunit va	F:GO:0004129,P:GO:0006118		0.88096
OctRet_0020F_027	BY797441	1.00E-27	76.7	cg14235 cg14235-pa	F:GO:0004129	Pfam-B_10366, COX6B	1.41544
OctRet_0045F_040	GS020503	0.008	32.2				
OctRet_0075F_071	BY799424	0.0004	38.6				
OctRet_0094F_096	BY799942	3.00E-08	54				
OctRet_0150F_060	BY800189	9.00E-07	35				
OctRet_0176F_089	gi|18524913|gb|BM475871.1|BM475871	0.008	41.4				
OctRet_0203F_017	gi|57590691|gb|CX563662.1|CX563662	2.00E-13	73	adaptor-related protein complexbeta 1 subunit isoform 2		Adaptin_N	
OctRet_0788F_027	gi|18522948|gb|BM473906.1|BM473906	2.00E-11	69.9				
OctRet_0840F_077	gi|22684611|gb|BU170627.1|BU170627	0.0006	46.1				
OctRet_0859F_087	GS004651	0.0002	38.2				

OctRet_0879F_017		GS020645	0.008	26.7			
OctRet_0892F_031		gi|54449888|gb|CV560841.1|CV560841	4.00E-14	79	alpha 2 type i collagen	C:GO:0005615,P:GO:0007169,F:GO:0005198	
OctRet_0896F_041		gi|41341368|gb|CK620482.1|CK620482	0.004	40.9			
OctRet_0914F_058		gi|19046508|gb|BM725177.1|BM725177	2.00E-07	55.6	---NA---		VHP
OctRet_0921F_054		gi|24721811|gb|CA390626.1|CA390626	0.008	44.1	two-component response regulator	P:GO:0000160,P:GO:0006355,F:GO:0000156,P:GO:0006350,F:GO:0003677	
OctRet_1082F_032		BY796825	0.009	35.4	integral membrane protein	C:GO:0016021	
OctRet_1112F_063	**Embryo specific expression**	BY797271	0.01	38.2			
OctRet_1170F_026		GS020760	0.008	28.1			
OctRet_1234F_090		BY798709	5.00E-16	81.8	---NA---		
OctRet_1240F_093		gi|15711152|gb|BI734139.1|BI734139	4.00E-10	42.3	nadh dehydrogenase subunit 2	C:GO:0005746,C:GO:0016020,C:GO:0005743,F:GO:0008137,C:GO:0005739,P:GO:0006810,P:GO:0006118,P:GO:0006120,F:GO:0016491,P:GO:0042773	
OctRet_1255F_005		gi|22673683|gb|BU159773.1|BU159773	9.00E-06	52			
OctRet_1272F_029		BY798753	0.009	38.2			

OctRet_1283F_035		GS004800	0.0006	34.2				
OctRet_1295F_049		gi|18986831|gb|BM676935.1|BM676935	0.0003	46				
OctRet_1300F_059		gi|3512121|gb|AI112172.1|AI112172	1.00E-06	53.3	glutathione s-transferase	F:GO:0005212		
OctRet_1353F_008	**Embryo specific expression**	BY797166	0.003	38.2				
OctRet_1370F_032		GS013955	0.004	34.2				
OctRet_1398F_062		gi|20363398|gb|BQ187847.1|BQ187847	0.0003	45.4	fyn-binding protein			
OctRet_1427F_085		gi|119789076|gb|EH281635.1|EH281635	5.00E-43	174	zinc finger protein 593	F:GO:0005488		0.29657
OctRet_1452F_025		BY794943	0.008	31.8	major facilitator superfamily mfs_1	P:GO:0006810,C:GO:0016021,F:GO:0005215		
OctRet_1465F_024		gi|62873846|gb|DN889083.1|DN889083	0	314	gtp-binding nuclear protein ran			0.70779
OctRet_1493F_054		gi|18501600|gb|BM452560.1|BM452560	2.00E-06	56				
OctRet_1499F_065		gi|19025292|gb|BM712034.1|BM712034	1.00E-41	169	developmental embryonic b cg16792-pa			1.1496
OctRet_1960F_047		gi|15682370|gb|BI706675.1|BI706675	9.00E-26	73	nadh dehydrogenase subunit 1	C:GO:0016020,F:GO:0016491		
SquidNo3_D02_004		gi|13297760|gb|BG404312.1|BG404312	0.006	44.1				
DB910987		gi|75836302|gb|DT866562.1|DT866562	1.00E-05	50.6	---NA---		RRM_1	
DB911028		GS013811	0.001	36.2				
DB911039		GS013856	0.005	34.2				

DB911042		BY797854	0.0007	42.1				
DB911058		gi|85202102|gb|DW605836.1|DW605836	1.00E-06	53.8	farnesyl diphosphate synthase	P:GO:0008299	polyprenyl_synt	
DB911092		BY799768	0.001	38.2				
DB911095		GS020506	0.003	32.2				
DB911100		gi|18988393|gb|BM678497.1|BM678497	0.0005	37.3				
DB911114		GS020551	0.0008	33.6			Pfam-B_4326	
DB911116		BY795311	2.00E-05	44.1	collagen triple helix repeat protein		Pfam-B_7579	
DB911121		gi|37631583|gb|CF735247.1|CF735247	0	233	ubiquitin-protein ligase		HECT	
DB911129		gi|19118735|gb|BM801912.1|BM801912	5.00E-26	111	eif4g1 protein	F:GO:0003743,P:GO:0006446,P:GO:0016070,F:GO:0005515	MA3	1.03727
DB911135		GS013972	0.005	34.2				
DB911138		gi|10204034|gb|BE782836.1|BE782836	0.008	30.4				
DB911148		GS020598	0.004	34.2				
DB911154		gi|37599777|gb|CF725609.1|CF725609	2.00E-22	73	beclin 1	C:GO:0005737	APG6	0.18435
DB911160		gi|16087554|gb|BI880283.1|BI880283	6.00E-23	108	novel proteinbtaf1 rna polymeraseb-tfiid transcription factor-170kda		SNF2_N	
DB911164		gi|10664305|gb|BE988186.1|BE988186	0.009	44.1				
DB911166		gi|45306394|gb|CK876763.1|CK876763	0.0006	46.1				
DB911193		gi|2221038|gb|AA490163.1|AA490163	4.00E-06	39.6	---NA---			
DB911208		GS004835	0.004	34.2				

DB911215		gi|9120961|gb|BE250824.1|BE250824	1.00E-19	96.8	rnps1 protein		RRM_1	1.54995
DB911218		GS020693	0.001	32.7				
DB911219		gi|49680852|gb|CO434558.1|CO434558	7.00E-20	84	protocadherin gamma-b1 precursor (pcdh-gamma-b1)		Cadherin,Cadherin	
DB911220		gi|49677571|gb|CO431277.1|CO431277	4.00E-24	88.1	protocadherin 2 alpha b 6	F:GO:0005262,F:GO:0005509,P:GO:0006816,F:GO:0004872,C:GO:0016020,F:GO:0019855	Cadherin,Cadherin	
DB911222		gi|13303397|gb|BG378925.1|BG378925	6.00E-22	104	acidic coiled-coil containing protein 3		CSD	1.82423
DB911228		GS020461	0.002	24.9				
DB911231		GS020499	0.001	36.2				
DB911242	Embryo specific	gi|9592398|gb|BB536898.1|BB536898	0.006	44.1				
DB911252		gi|37632734|gb|CF736398.1|CF736398	0.0001	43.7				
DB911287		BY795259	5.00E-05	43.2	prothoracicostatic peptide precursor	F:GO:0005179,P:GO:0007218,F:GO:0003674,C:GO:0005615,P:GO:0002168,C:GO:0005576		
DB911294		GS013930	0.007	30.4				
DB911295		gi|10589030|gb|BE950364.1|BE950364	0.007	44.1				
DB911297		gi|37638356|gb|CF742017.1|CF742017	1.00E-24	84.5	lethalk05713 cg8256-isoform c			
DB911307		BY800436	0.003	40.1				
DB911311		gi|29801445|gb|CB734270.1|CB734270	0.009	40.1				

DB911319		gi|46814677|gb|CN502053.1|CN502053	9.00E-40	116	regulator of g protein signaling		RGS	0.64515
DB911322		BY795816	0.0008	42.1				
DB911332		gi|62883314|gb|DN898551.1|DN898551	2.00E-25	75.3	n-ethylmaleimide sensitive fusion protein attachment protein alpha	C:GO:0005783,C:GO:0005794,P:GO:0006886,F:GO:0005488		
DB911351		gi|54445890|gb|CV558860.1|CV558860	2.00E-32	128	loc100037132 protein		HEAT,Pfam-B_7274	
DB911354		gi|62829115|gb|DN859466.1|DN859466	4.00E-06	48.7	sjchgc04589 protein		Alpha_adaptin_C	
DB911365		GS020607	0.004	34.2				
DB911367		GS020638	0.001	36.2				
DB911370	Embryo specific	BY799356	0.007	38.2				
DB911375		gi|41338165|gb|CK617279.1|CK617279	2.00E-41	169	isoform cra_a	C:GO:0016021	DUF872	0.1498
DB911409		GS004694	0.005	34.2	fvriamide neuropeptide precursor			
DB911412		gi|20361740|gb|BQ186189.1|BQ186189	0.002	46.1				
DB911425		gi|54446990|gb|CV559416.1|CV559416	0	325	cell division cycleisoform cra_c		Pfam-B_614,TPR_2,TPR_1	
DB911430		BY796875	0.0008	38.6	---NA---			
DB911432		gi|58031823|gb|CX729366.1|CX729366	1.00E-13	74.4	synapsin s-syn-long	P:GO:0007269,C:GO:0008021,F:GO:0005524,F:GO:0003824	Synapsin	1.10337
DB911433		gi|75851599|gb|DT881859.1|DT881859	3.00E-26	118	ghitm-prov protein	C:GO:0016021		0.14023
DB911436		BY795783	0.01	38.2				

DB911442		gi|19050388|gb|BM729055.1|BM729055	5.00E-10	58.3	n-acetylglucosamine-1-phosphodiester alpha-n-			
DB911447		gi|37601933|gb|CF727765.1|CF727765	3.00E-32	93.2	protein dpy-19 homolog 1 (dpy-19-like protein 1)		Dpy19	
DB911490		gi|22697937|gb|BU183953.1|BU183953	3.00E-41	169	26s proteasome non-atpase regulatory subunit 13	P:GO:0007127,C:GO:0005838,F:GO:0004175		0.20873
DB911493		BY795421	0.008	38.2				
DB911510		gi|23668550|gb|BU735783.1|BU735783	0.002	46.1			Pfam-B_13263,Pfam-B_4326, Pfam-B_13263,Pfam-B_4326	
DB911512		GS020659	0.005	30.9				
DB911532		gi|40549684|gb|CK396152.1|CK396152	0.0006	48.1				
DB911549		gi|46816411|gb|CN503787.1|CN503787	0	244	aspartate aminotransferase	F:GO:0030170,F:GO:0004069,P:GO:0006520,P:GO:0009058	Aminotran_1_2	0.86328
DB911573		BY799426	0.0006	42.1				
DB911577		gi|10653523|gb|BE982898.1|BE982898	6.00E-39	116	ring finger protein 166	F:GO:0046872,F:GO:0008270,F:GO:0005515,C:GO:0005622	zf-C3HC4, Pfam-B_10993, Di19,Pfam-B_49, Pfam-B_14559	0.2121
DB911585		gi|54449824|gb|CV560809.1|CV560809	0	372	actin related protein 2 3 complex subunit 2		P34-Arc,PmbA_TldD	0.08341

DB911601		gi|62046427|gb|DN691747.1|DN691747	1.00E-08	61.1	kelch domain containing 10	F:GO:0003674,P:GO:0008150	Kelch_2, Kelch_1, Pfam-B_15464, Kelch_1	
DB911607	Embryo specific	gi|37635569|gb|CF739232.1|CF739232	2.00E-06	53.3	---NA---			
DB911620		gi|54444728|gb|CV558300.1|CV558300	3.00E-15	68.9	fatty acid synthase	F:GO:0004312	Thioesterase, AroM	
DB911623		gi|62847148|gb|DN877193.1|DN877193	0	220	g protein a subunit o class	P:GO:0007186,F:GO:0004871,F:GO:0005525	G-alpha, G-alpha	
DB911626		gi|23675414|gb|BU738380.1|BU738380	2.00E-27	123	protoheme ix		UbiA	
DB911632		gi|37638356|gb|CF742017.1|CF742017	2.00E-09	59.2	elegans proteinconfirmed by transcript evidence			
DB911636		gi|85193203|gb|DW602890.1|DW602890	4.00E-17	88.1	proline dehydrogenase1	P:GO:0009064		
DB911657		gi|9129209|gb|BE258716.1|BE258716	6.00E-33	130	ribosomal protein s24	C:GO:0005840,P:GO:0006412,F:GO:0000166	Ribosomal_S24e	2.10946
DB911685		BY796756	5.61E-45	163	peanut cg8705-isoform a	C:GO:0031105,P:GO:0007049,F:GO:0005515,F:GO:0005525	Septin,Pfam-B_6895	0.2253
DB911733		gi|49675470|gb|CO429176.1|CO429176	3.00E-21	58.8	---NA---		DEAD	
DB911734		gi|57592667|gb|CX565638.1|CX565638	5.00E-31	134	alsin isoform 1		VPS9	
DB911735		GS019915	0.001	36.2				
DB911736		gi|45305310|gb|CK875679.1|CK875679	3.00E-09	58.3	ubiquinol-cytochrome c reductase core protein ii		Peptidase_M16_C	

DB911746		gi|54449981|gb|CV560887.1|CV560887	9.00E-30	121	parkinson disease 7 domain containing 1	C:GO:0005576		1.00135
DB911755		gi|49670748|gb|CO424537.1|CO424537	0	132	ank2 cg7462-isoform b			2.2658
DB911758		gi|19024436|gb|BM711178.1|BM711178	6.00E-12	54.2	acid phosphatase-like 2 isoform 7		Acid_phosphat_A	
DB911789		gi|23690556|gb|BU743218.1|BU743218	0.0005	44.1				
DB911792		gi|62881333|gb|DN896570.1|DN896570	9.00E-32	134	plasma alpha-l-fucosidase	P:GO:0005975,F:GO:0043169,F:GO:0004560	Alpha_L_fucos	1.50302
DB911794		gi|15712379|gb|BI735366.1|BI735366	1.00E-11	55.1	organic anion transporter		OATP	
DB911802		gi|19121051|gb|BM804228.1|BM804228	0.003	41.8				
DB911803		GS020635	0.001	32.7				
DB911817		gi|18519436|gb|BM470394.1|BM470394	0	125	chromosome 12 open reading frame 11 isoform 3		DUF2151, DUF2151	
DB911823		gi|49671586|gb|CO425292.1|CO425292	1.00E-42	172	novel protein (zgc:100937)	C:GO:0005576,F:GO:0016787	Pfam-B_10797	
DB911825		gi|32273159|gb|CD722311.1|CD722311	0	187	isoform cra_a	C:GO:0030176,C:GO:0005794,P:GO:0046856,F:GO:0016791,C:GO:0005579	Syja_N, Syja_N	0.5172
DB911830		gi|22673755|gb|BU159845.1|BU159845	0	222	novel proteinvertebrate asparaginyl-trna synthetase		tRNA_anti	0.28049
DB911835		GS020564	0.004	31.3				
DB911845		GS008151	0.004	30.9				

DB911846		gi|62848950|gb|DN878995.1|DN878995	0.0001	50.1				
DB911849		gi|62848099|gb|DN878144.1|DN878144	4.06E-44	177	glutamine synthetase	F:GO:0004356,P:GO:0006542	Gln-synt_C	1.68752
DB911867		gi|57595696|gb|CX568667.1|CX568667	2.00E-38	107	unc-13 cg2999-pc		Membr_traf_MHD	
DB911869		gi|6515841|gb|AW209901.1|AW209901	5.00E-31	134	phosphoglycerate kinase	P:GO:0006096,F:GO:0004618	PGK	0.88533
DB911882		GS014134	0.005	34.2	isoform d		PNP_UDP_1	1.0885
DB911896		gi|18500742|gb|BM451702.1|BM451702	1.00E-34	147	t-complex 11		Baculo_p24, Tcp11	0.89087
DB911898		gi|85169082|gb|DW595077.1|DW595077	2.00E-32	70.8	nervous system adducin	F:GO:0046872	Aldolase_II	0.6202
DB911907		GS013851	0.004	34.2				
DB911910		BY797251	0.002	31.3				
DB911944		gi|62851116|gb|DN881161.1|DN881161	0.004	42.1				
DB911946		gi|21761521|gb|BQ637062.1|BQ637062	1.00E-07	58	splicing factor u2af35			
DB911981		gi|58035234|gb|CX732760.1|CX732760	4.00E-07	55.1	cacna1d protein	C:GO:0043234,C:GO:0009925,C:GO:0016021,P:GO:0050910,F:GO:0005245,P:GO:0050885,P:GO:0030001	Ion_trans	4.0402
DB911987		GS020461	0.005	30.9				
DB912007		gi|85209205|gb|DW608782.1|DW608782	3.00E-43	152	dynamin 1-like	C:GO:0005579,C:GO:0005737,F:GO:0003924,F:GO:0005525	Dynamin_M, NUC202,DUF1308	

<table>
<tr><td>DB912010</td><td></td><td>BY795488</td><td>0.003</td><td>36.2</td><td></td><td></td><td></td><td></td></tr>
<tr><td>DB912015</td><td></td><td>gi|85198668|gb|DW604674.1|DW604674</td><td>0</td><td>279</td><td>nadh dehydrogenasefe-s protein 1 isoform 1</td><td></td><td>Molybdopterin</td><td>0.063</td></tr>
<tr><td>DB912017</td><td></td><td>gi|62040333|gb|DN688742.1|DN688742</td><td>0</td><td>209</td><td>eukaryotic initiation factor 4a</td><td>C:GO:0005737,F:GO:0008026,P:GO:0043581,F:GO:0003723,F:GO:0005524,F:GO:0003743,P:GO:0006412</td><td>DEAD</td><td>1.7499</td></tr>
<tr><td>DB912030</td><td></td><td>gi|62882476|gb|DN897713.1|DN897713</td><td>0.003</td><td>42.8</td><td>ring finger and fyve-like domain containing 1</td><td></td><td>FYVE</td><td></td></tr>
<tr><td>DB912036</td><td></td><td>gi|75850091|gb|DT880351.1|DT880351</td><td>0</td><td>164</td><td>zinc finger protein 403 isoform 2</td><td></td><td>Pfam-B_14984</td><td>1.11</td></tr>
<tr><td>DB912038</td><td></td><td>gi|62850341|gb|DN880386.1|DN880386</td><td>0.003</td><td>38.2</td><td></td><td></td><td></td><td></td></tr>
<tr><td>DB912040</td><td></td><td>gi|18500266|gb|BM451226.1|BM451226</td><td>3.00E-34</td><td>119</td><td>membrane-bound transcription factorsite 2</td><td>P:GO:0006629,F:GO:0008233,P:GO:0001501</td><td>Peptidase_M50</td><td></td></tr>
<tr><td>DB912055</td><td></td><td>gi|15682687|gb|BI706992.1|BI706992</td><td>5.00E-20</td><td>98.2</td><td>rpa43_danreame: full=dna-directed rna polymerase i subunit rpa43ame: full=twist neighbor protein</td><td>P:GO:0006355,P:GO:0006353,F:GO:0003899,P:GO:0031564,C:GO:0005634</td><td>RNA_pol_Rpb7_N, Pfam-B_7275</td><td>0.8213</td></tr>
<tr><td>DB912058</td><td></td><td>gi|46465840|gb|CN460114.1|CN460114</td><td>7.00E-07</td><td>51</td><td>cationic amino acid transporter</td><td></td><td></td><td></td></tr>
<tr><td>DB912064</td><td></td><td>gi|85200105|gb|DW605137.1|DW605137</td><td>0.009</td><td>44.1</td><td>carboxypeptidase a2</td><td>F:GO:0046872,F:GO:0004182,P:GO:0006508,F:GO:0004180,F:GO:0008237,F:GO:0008233,F:GO:0008270,C:GO:0005576</td><td>Pfam-B_5672, Propep_M14</td><td></td></tr>
</table>

DB912071		gi|77506737|gb|DV216687.1|DV216687	5.00E-15	78.4	voltage-dependent anion channel 2	P:GO:0006820,F:GO:0008308,C:GO:0016021,C:GO:0005741	Porin_3	
DB912078		GS020765	0.0005	34.1				
DB912083		BY795194	3.00E-24	103	septin 5	C:GO:0031105,P:GO:0007049,F:GO:0005515,F:GO:0005525	Septin	
DB912108		gi|46867400|gb|CN539244.1|CN539244	4.00E-37	107	cg31064-isoform a			
DB912109	**Positive selection**	gi|62890243|gb|DN905480.1|DN905480	0	253	adp-ribosylation factorisoform cra_a	F:GO:0005525,C:GO:0005794,F:GO:0005515,F:GO:0008565,P:GO:0016192,P:GO:0006886,P:GO:0007264	Arf	6.51203
DB912128		gi|18983922|gb|BM674024.1|BM674024	0.008	44.1				
DB912132		gi|18987621|gb|BM677725.1|BM677725	0.0007	40.5	ralbp1 associated eps domain containing 1	F:GO:0005509		1.43775
DB912165		gi|11661619|gb|BF551895.1|BF551895	6.00E-42	171	spermatogenesis associated 5 isoform 1		AAA	
DB912188		gi|46466837|gb|CN461111.1|CN461111	0	184	taf1 rna polymerasetata box binding protein-associated factor	F:GO:0003677,F:GO:0016301,C:GO:0005669,P:GO:0007049,F:GO:0005524,P:GO:0006352,P:GO:0006355	Pfam-B_17638	1.2438
DB912210		GS008140	0.003	34.2				
DB912235		GS020592	0.004	34.2				
DB912260		gi|32465039|gb|CD806213.1|CD806213	6.00E-06	37.7				

DB912261		gi|85195632|gb|DW603601.1|DW603601	0.0005	41.8				
DB912271		GS004800	0.005	34.2				
DB912272	**Embryo specific**	GS014137	0.003	31.3				
DB912276		gi|54444960|gb|CV558410.1|CV558410	0	304	tfg beta signaling pathway factor		MH2	0.92965
DB912284		gi|18511337|gb|BM462297.1|BM462297	2.00E-39	162	peptidase (mitochondrial processing) alpha	F:GO:0004222,P:GO:0006508,F:GO:0008270	Peptidase_M16_C, Peptidase_M16	1.05023
DB912290		gi|62829138|gb|DN859489.1|DN859489	0	322	hippocampus abundant gene transcript 1	P:GO:0015904,C:GO:0016021,P:GO:0046677,F:GO:0015520	MFS_1	
DB912291		BY799598	0.003	36.2				
DB912292		BY798825	0.002	40.1				
DB912296		gi|13102735|gb|BG305208.1|BG305208	0	194	histone h4		Histone	
DB912302		gi|14063722|gb|BG753069.1|BG753069	0	156	novel proteinvertebrate imp (inosine monophosphate) dehydrogenase 1	F:GO:0003938,P:GO:0006177	IMPDH, IMPDH	
DB912310		gi|8486815|gb|BE095884.1|BE095884	0.002	42.8			Pfam-B_4326, Pfam-B_4326, Pfam-B_4326	
DB912312		gi|48584931|gb|CO044391.1|CO044391	0.002	46.1				
DB912317		gi|62026626|gb|DN681905.1|DN681905	3.00E-41	166	guanine nucleotide-binding protein rho	C:GO:0005622,C:GO:0005886,F:GO:0005525,P:GO:0007264	Ras	0.45133
DB912318		gi|75837350|gb|DT867610.1|DT867610	0	334	thioredoxin peroxidase		1-cysPrx_C,AhpC-TSA	0.30859

DB912336		gi|22675937|gb|BU162027.1|BU162027	0	284	dihydrolipoamide dehydrogenase	P:GO:0045454,C:GO:0005759,P:GO:0006118,F:GO:0050660,F:GO:0004148	Pyr_redox_2, Pyr_redox	0.73524
DB912343		gi|62886290|gb|DN901527.1|DN901527	0.001	34.1				
DB912355		BY799220	0.01	38.2				
DB912367		gi|49675765|gb|CO429471.1|CO429471	2.00E-26	119	17beta-hydroxysteroid dehydrogenase	F:GO:0042802	MaoC_dehydratas	
DB912384		GS020656	0.007	30.4			Pfam-B_13263, Pfam-B_4326, Pfam-B_4326	
DB912394		gi|62882970|gb|DN898207.1|DN898207	0	327	eukaryotic translation initiation factor 5b (eif-5b) (translation initiation factor if-2)		IF-2	2.34655
DB912397		gi|10201276|gb|BE780078.1|BE780078	0.0007	40.9				
DB912402		gi|9889423|gb|BE618485.1|BE618485	0	161	---NA---		Pfam-B_2320	
DB912418		gi|37598576|gb|CF724408.1|CF724408	1.00E-12	71.2	atp-dependent bile acid permease		ABC_membrane	
DB912433		BY796799	1.00E-05	48.1				
DB912448		gi|18979105|gb|BM669208.1|BM669208	8.00E-29	127	macro domain containing 2		Macro	0.93433
DB912452		BY798426	0.008	38.2				
DB912477		gi|62842873|gb|DN872932.1|DN872932	0	303	traf2 and nck interactingtnik		CNH	
DB912483		GS020551	0.009	29.9				
DB912526		BY796100	0.009	38.2				

DB912527		gi|49679435|gb|CO433141.1|CO433141	6.00E-33	141	kiaa1636 protein		Ank, Ank, Ank, Ank, Ank	0.90513
DB912528		gi|37633763|gb|CF737427.1|CF737427	1.00E-07	57.4	---NA---		Pfam-B_10577	
DB912532	Embryo specific	GS008127	2.00E-05	34.1				
DB912537		gi|10203700|gb|BE782502.1|BE782502	3.00E-23	109	synovial apoptosis inhibitorsynoviolin	F:GO:0016874,P:GO:0006829,C:GO:0005579,C:GO:0005786,P:GO:0009968,F:GO:0008270	Pfam-B_1065, Pfam-B_133,Pfam-B_5058,Pfam-B_11973,Pfam-B_16964,Pfam-B_17208,Pfam-B_10669,Pfam-B_39 ,Pfam-B_14559,zf-C3HC4,Pfam-B_4755	
DB912542		BY795001	0.005	30.9				
DB912546		BY795304	0.002	37.7				
DB912549		gi|19046685|gb|BM725354.1|BM725354	0.002	46.1				
DB912575		gi|57591445|gb|CX564416.1|CX564416	5.00E-11	68.4	sjchgc04791 protein			
DB912582		gi|62045919|gb|DN691488.1|DN691488	2.80E-45	182	subfamilymember 2		DnaJ_C	0.78315
DB912590		gi|19047029|gb|BM725696.1|BM725696	3.00E-10	65.7	atp-bindingsub-family b (mdr tap)member 6	F:GO:0042626,P:GO:0006810,F:GO:0005524,C:GO:0016021,C:GO:0005741		
DB912621		BY799703	0.002	37.7				

DB912631		gi|62887310|gb|DN902547.1|DN902547	0.009	44.1				
DB912649		gi|21762007|gb|BQ637548.1|BQ637548	5.00E-07	54.9	ran gtpase-activating protein	F:GO:0005515	RanGAP1_C, Pfam-B_12569,Nop14, Pfam-B_2333, Pfam-B_5586	
DB912660		GS004800	0.004	34.2				
DB912664		gi|21765658|gb|BQ641486.1|BQ641486	2.00E-39	93.7	annexin a7		Annexin, Annexin	
DB912668		GS004800	0.003	34.2				
DB912676		gi|37637992|gb|CF741653.1|CF741653	0.0003	45.7	thioester-containing protein	F:GO:0004866,F:GO:0005515,C:GO:0005576	A2M_comp	
DB912681		BY795985	0.009	35.9				
DB912686		GS013829	0.004	34.2				
DB912687		BY798965	3.00E-17	71.8	fascin homolog 1-actin-bundling protein	F:GO:0030674	DUF3656, Fascin,Fascin	
DB912700		gi|19028434|gb|BM715176.1|BM715176	2.00E-13	76.2	-like subfamily b member 6	F:GO:0031072		
DB912701		gi|22345651|gb|BQ930620.1|BQ930620	1.00E-16	57.9	classmember 2		Glyco_hydro_38C	0.8353
DB912707		gi|24722033|gb|CA390743.1|CA390743	0	325	bone marrow stromal cell-derived ubiquitin-like protein	P:GO:0006512	ubiquitin	
DB912714		gi|85205120|gb|DW607189.1|DW607189	0	377	nadh dehydrogenaseflavoprotein51kda	P:GO:0006120,F:GO:0051287,F:GO:0008137,F:GO:0005506,F:GO:0010181,P:GO:0006118,P:GO:0006810,F:GO:0051539,C:GO:0005746	Complex1_51K, SLBB	0.09373

DB912738		gi|24543901|gb|CA325803.1|CA325803	6.00E-16	85.4	mitochondrial carrier homologisoform a	C:GO:0005739		0.40836
DB912745		gi|75835694|gb|DT865954.1|DT865954	7.00E-22	54.3	survival motor neuron domain containing 1	F:GO:0003676,F:GO:0003723,P:GO:0006397,P:GO:0000245,C:GO:0005634	Pfam-B_7939, Pfam-B_2444, SMN	0.49777
DB912762		gi|62044561|gb|DN690812.1|DN690812	2.00E-26	113	ubiquinone biosynthesis monooxygenase coq6	P:GO:0006725,F:GO:0016491,C:GO:0005615,P:GO:0006744,P:GO:0008152,F:GO:0016709,C:GO:0005575,F:GO:0050660		1.48683
DB912768		gi|62871874|gb|DN887111.1|DN887111	7.00E-13	74.8	chloride channelpartial		CBS	
DB912772		gi|32466934|gb|CD808108.1|CD808108	1.40E-45	183	kiaa0020 protein			
DB912773		gi|62843107|gb|DN873166.1|DN873166	7.00E-16	84.9	nuclear lamin l1		FYVE, IBR	
DB912776		BY795642	2.00E-06	44.6			bZIP_2	
DB912793		gi|77502459|gb|DV214503.1|DV214503	2.00E-06	52.8	zinc finger		Pfam-B_1393	0.7937
DB912802		gi|58178950|gb|CX768597.1|CX768597	4.00E-18	91.8	pyruvate dehydrogenasecomponentpartial		2-oxoacid_dh	0.29127
DB912820		GS020723	0.002	32.2				
DB912827		gi|37632506|gb|CF736170.1|CF736170	0	307	eukaryotic peptide chain release factor		eRF1_3	
DB912831		gi|62024788|gb|DN680985.1|DN680985	2.00E-18	52.8	lethalk09913 cg3082-pc		DUF829	

DB912832		gi|32175105|gb|CD673374.1|CD673374	0	244	fig4 homolog			0.96493
DB912835		gi|22337291|gb|BQ922260.1|BQ922260	0	106	mannose-6-phosphate isomerase		PMI_typeI	0.73762
DB912837		gi|62839120|gb|DN869179.1|DN869179	0	371	adaptor-related protein complexmu 1a	P:GO:0006461,F:GO:0008289,C:GO:0005579,F:GO:0008565,P:GO:0016192,P:GO:0006886,C:GO:0030131,C:GO:0030132	Adap_comp_sub	0.3036
DB912843		gi|62033477|gb|DN685282.1|DN685282	2.00E-27	122	26s proteasome non-atpase regulatory subunit 3			0.90891
DB912845		gi|19013482|gb|BM700224.1|BM700224	6.00E-24	107	c-terminal srcisoform g	F:GO:0035014,P:GO:0002119,P:GO:0006468,C:GO:0005942,F:GO:0005515,F:GO:0005524,F:GO:0004713,F:GO:0004674	Pkinase_Tyr	
DB912849		gi|4563757|gb|AI579381.1|AI579381	0.009	44.1				
DB912863		gi|22811051|gb|BU504818.1|BU504818	9.00E-21	100	tfp11_boviname: full=tuftelin-interacting protein 11ame: full=septin and tuftelin-interacting protein 1 short=stip-1	F:GO:0003676,C:GO:0005622	Pfam-B_6436	
DB912872		GS014037	0.001	36.2				
DB912880		gi|12354902|gb|BF937582.1|BF937582	3.00E-30	88.9	fasciculation and elongation protein zeta 1 (zygin i) isoform 3		FEZ	

DB912893	**Embryo specific**	gi|62836036|gb|DN866387.1|DN866387	5.00E-15	82	kiaa1738 protein	F:GO:0003674,P:GO:0008150	DUF2404, SOG2	
DB912907		gi|19011979|gb|BM698721.1|BM698721	1.40E-45	112	flj39378 protein isoform 2		RILP	
DB912914		gi|119789254|gb|EH281813.1|EH281813	0.002	39.8				
DB912940		GS020580	0.0008	36.2				
DB912945		gi|54480453|gb|CV575153.1|CV575153	4.00E-05	52				
DB912963		gi|12040252|gb|BF724343.1|BF724343	7.00E-39	134	kuzbanian cg7147-isoform a		Pfam-B_14539	
DB912969		gi|37600899|gb|CF726731.1|CF726731	0.003	43.2	---NA---			
DB912977		gi|15716370|gb|BI739357.1|BI739357	0.009	44.1				
DB912982		gi|85171716|gb|DW596007.1|DW596007	1.00E-25	81.4	odo2_fugruame: full=dihydrolipoyllysine-residue succinyltransferase component of 2-oxoglutarate dehydrogenasemitochondrialame: full=dihydrolipoamide succinyltransferase component of 2-oxoglutarate dehydrogenase complex short=e2ame: full=e2k flags: precursor	C:GO:0045252,F:GO:0004149,P:GO:0006099,F:GO:0031405,C:GO:0005739	2-oxoacid_dh, Biotin_lipoyl	1.4637
DB912985		gi|62842042|gb|DN872101.1|DN872101	0.009	44.1				

DB912988		gi|9388719|gb|BB471530.1|BB471530	0.01	44.1				
DB912994		gi|85193203|gb|DW602890.1|DW602890	0	176	proline dehydrogenase1	F:GO:0004657,P:GO:0006537,P:GO:0006562		
DB913003		gi|46563334|gb|CN481830.1|CN481830	0.002	46.1				
DB913024		GS013784	0.003	31.3				
DB913029		BY797909	0.003	40.1				
DB913033		GS014023	0.001	36.2				
DB913048		gi|62024575|gb|DN680892.1|DN680892	0	141	yip1 domainmember 1 isoform 6		Pfam-B_3433, Yip1	
DB913064		BY799008	0.004	36.2				
DB913066		gi|62828989|gb|DN859340.1|DN859340	3.00E-38	123	ribosomal protein s30	P:GO:0044267,C:GO:0005622	ubiquitin,Pfam-B_13282,Ribosomal_S30	0.17947
DB913089	GO annotation	gi|6515598|gb|AW209658.1|AW209658	0	202	ets-related isoform 4		Ets, Pfam-B_2481	0.7548
DB913126		gi|49672968|gb|CO426674.1|CO426674	8.00E-26	116	adaptin ear-binding clathrin-associated protein		DUF1681	0.3036
DB913128		gi|41339610|gb|CK618724.1|CK618724	0	385	heat shock protein 90	P:GO:0006457,F:GO:0051082,F:GO:0005524,P:GO:0006950	HSP90	0.23848
DB913131		gi|41345269|gb|CK624383.1|CK624383	1.00E-11	70.7	spermatogenesisserine-rich 2		DUF1387	
DB913138	Embryo specific	GS020726	0.005	30.9	---NA---			

DB913145		gi|24723938|gb|CA391768.1|CA391768	8.00E-06	43.7			Pfam-B_4326, Pfam-B_13263,Pfam-B_4326, Pfam-B_4326	
DB913153		GS004679	0.005	34.2				
DB913156		gi|62040139|gb|DN688645.1|DN688645	1.00E-21	88.1	mgc68804 protein		Pfam-B_14351	1.1158
DB913161		gi|10204338|gb|BE783140.1|BE783140	7.00E-15	80.9	son dna-binding protein		Pfam-B_2818	
DB913165		gi|40321032|gb|CK355100.1|CK355100	0	247	proteasome subunit n3		Proteasome	1.8665
DB913168		gi|57592920|gb|CX565891.1|CX565891	0	250	adp-ribosylation factor interacting protein 2b	F:GO:0003674,C:GO:0005575	Arfaptin	
DB913174		gi|9122814|gb|BE252671.1|BE252671	2.00E-28	108	hcls1 binding protein 3		PX	1.9744
DB913226	**Embryo specific expression, Positive selection**	gi|62846546|gb|DN876605.1|DN876605	0	286	g alpha i subunit	P:GO:0007186,F:GO:0004871,F:GO:0005525	G-alpha	3.07597
DB913236		gi|13063968|gb|BG298876.1|BG298876	0.0005	48.1				
DB913237		gi|15709338|gb|BI732325.1|BI732325	0	188	taurine transporter	C:GO:0005887,F:GO:0005328,P:GO:0006836	SNF	1.32602
DB913238		GS020774	0.003	29				
DB913240		GS008202	0.008	30.2				
DB913241		BY799689	0.002	40.1				
DB913244	**Positive selectior**	gi|62832237|gb|DN862588.1|DN862588	2.00E-36	150	25-kda subunit rna polymerase	P:GO:0006350,F:GO:0003899,C:GO:0005634,F:GO:0003677	RNA_pol_Rpb5_N	5.44031
DB913265		gi|18801967|gb|BM558864.1|BM558864	3.00E-11	69.3	hspb (heat shock 27kda) associated protein 1			

DB913273		BY795432	0.001	35.4				
DB913319		gi|58033543|gb|CX731069.1|CX731069	3.00E-41	104	light polypeptide	C:GO:0030132,F:GO:0005509	DUF1092, Clathrin_lg_ch	1.20868
DB913324		gi|37600957|gb|CF726789.1|CF726789	0.006	35.4				
DB913332		gi|46825971|gb|CN513347.1|CN513347	0	227	isoform a	C:GO:0035003,C:GO:0045211,F:GO:0005515	Pfam-B_4599,PDZ	0.02862
DB913333		gi|62888698|gb|DN903935.1|DN903935	0	194	cytochrome c	F:GO:0009055,F:GO:0005506,C:GO:0005759,F:GO:0020037,P:GO:0006118,P:GO:0006810,C:GO:0005746	Cytochrom_C	0.55094
DB913334		gi|85570838|gb|DW711454.1|DW711454	2.00E-08	51.3	cleavage and polyadenylation specificity			
DB913335		gi|18514281|gb|BM465239.1|BM465239	0.003	46.1				
DB913340	**Embryo specific**	gi|22667862|gb|BU154330.1|BU154330	0	184	inositol trisphosphate receptor	F:GO:0005262,P:GO:0006811,C:GO:0016020		0.86123
DB913348		BY800050	0.003	35	---NA---			
DB913369		gi|29772761|gb|CB715613.1|CB715613	0.0007	48.1				
DB913375		BY798681	0.003	40.1				
DB913395		GS014081	0.006	34.2				
DB913418		gi|3396572|gb|AI070321.1|AI070321	0.002	46.1				
DB913420		gi|85179361|gb|DW598441.1|DW598441	0.0003	42.3	prohormone convertase 2	F:GO:0004289,P:GO:0006508,F:GO:0004252,F:GO:0016787,F:GO:0008233		

DB913426		gi|62046021|gb|DN691540.1|DN691540	0	402	retinal dehydrogenase	F:GO:0016491,P:GO:0008152	Aldedh	1.1106
DB913439		BY795176	0	238	von hippel-lindau binding protein 1	P:GO:0006457,C:GO:0016272,C:GO:0005634,F:GO:0005515	Prefoldin	0.89993
DB913440		gi|40549504|gb|CK395972.1|CK395972	5.00E-05	48.7	---NA---		Ldl_recept_a	
DB913441		gi|32176228|gb|CD674497.1|CD674497	0.0002	50.1				
DB913442		gi|62037138|gb|DN687131.1|DN687131	0	256	actin-related protein 2 3		WD40, WD40	
DB913449		gi|9385047|gb|BB467858.1|BB467858	0.009	44.1				
DB913450		gi|62885542|gb|DN900779.1|DN900779	0.007	41.8	---NA---			
DB913487		GS004800	0.004	34.2				
DB913490		GS013979	0.004	25.4				
DB913493		gi|19390371|gb|BM931198.1|BM931198	2.00E-30	133	small subunitprocessomehomolog	F:GO:0003674,P:GO:0008150	Fcf1	
DB913494		gi|18510675|gb|BM461635.1|BM461635	0	131	gamma-glutamyl carboxylase	C:GO:0016020	VKG_Carbox, Phytoreo_P8, Cupin_2	
DB913503		gi|62040707|gb|DN688924.1|DN688924	4.00E-35	149	cg7766-isoform b isoform 1		Pfam-B_1757	

DB913522		gi|62847335|gb|DN877380.1|DN877380	0	314	glycogen phosphorylase	F:GO:0004645,P:GO:0005975,F:GO:0030170,F:GO:0043565,C:GO:0005622,F:GO:0003700,P:GO:0006355	Phosphorylase	0.63607
DB913528		gi|85168270|gb|DW594674.1|DW594674	1.00E-06	37.3	cl066_xenlaame: full=upf0536 protein c12orf66 homolog		DUF2003	
DB913535	Embryo specific	BY798736	0.01	29	serine arginine repetitive matrix 1	P:GO:0006397,F:GO:0003677,C:GO:0016363,P:GO:0008380,F:GO:0031202,F:GO:0003723,C:GO:0005681,P:GO:0000398,F:GO:0005515		
DB913547		gi|75835685|gb|DT865945.1|DT865945	0	223	ring-box protein 2			1.07992
DB913549		gi|58061026|gb|CX734190.1|CX734190	1.40E-45	184	run and fyve domain containing 2	F:GO:0046872,F:GO:0008270,C:GO:0005634	Pfam-B_13880,Pfam-B_13880	1.5647
DB913569		gi|46823126|gb|CN510502.1|CN510502	1.00E-15	62.9	h+v1 subunit f		ATP-synt_F	
DB913590		gi|32180832|gb|CD679101.1|CD679101	0.003	46.1				
DB913606		GS020725	0.004	34.2	---NA---			
DB913609		GS008119	0.005	34.2				
DB913627		gi|22339549|gb|BQ924518.1|BQ924518	3.00E-05	49.6	protocadherin 17	F:GO:0005509,C:GO:0016021,C:GO:0016020,F:GO:0005515	Cadherin_2	
DB913637		gi|13298119|gb|BG404671.1|BG404671	0	236	adaptor-related protein complexalpha 1 subunit		Alpha_adaptin_C, Alpha_adaptinC2	0.60157

DB913650		gi|62046989|gb|DN692025.1|DN692025	3.00E-19	96.4	novel protein (zgc: 92136)	C:GO:0044464		0.7395
DB913674		gi|24729312|gb|CA394606.1|CA394606	0.0007	48.1			RRM_1	
DB913685		gi|62883652|gb|DN898889.1|DN898889	3.00E-20	73.5	rho guanine exchange factor 15		SH3_1	
DB913694		BY795432	1.00E-05	45.5	---NA---			
DB913697		gi|37600573|gb|CF726405.1|CF726405	0	349	frizzled 1		Frizzled, Frizzled	0.88177
DB913711	Positive selectior	gi|22695367|gb|BU181383.1|BU181383	0	257	cop9 constitutive photomorphogenic homolog subunit 3	C:GO:0005737,C:GO:0008180		3.57667
DB913719		gi|77503914|gb|DV215332.1|DV215332	0	463	rna binding motif protein 22	P:GO:0008380,C:GO:0005681,F:GO:0003723,P:GO:0006397,F:GO:0008270	zf-CCCH, Pfam-B_18962	4.4089
DB913720		gi|62038980|gb|DN688060.1|DN688060	0	233	prolyl endopeptidase	P:GO:0006508,F:GO:0004287	Peptidase_S9_N	0.6695
DB913726		gi|18527507|gb|BM478465.1|BM478465	0	245	gdp dissociation inhibitor	P:GO:0007269,P:GO:0043087,C:GO:0008021,P:GO:0016192,F:GO:0005093,P:GO:0015031	GDI	
DB913730		gi|21765959|gb|BQ641787.1|BQ641787	0	183	member ras oncogene family		Ras	0.0971
DB913738		gi|15586332|gb|BI670948.1|BI670948	2.00E-25	116	zgc:153540 protein	P:GO:0006546,F:GO:0004047	GCV_T, GCV_T_C	
DB913744		gi|46465494|gb|CN459768.1|CN459768	0	334	swi snfmatrixactin dependent regulator ofsubfamilymember 1		Pfam-B_9568, SLIDE	1.4354
DB913750		GS020461	0.006	34.2	mucin 2 precursor	F:GO:0005488,C:GO:0009279,F:GO:0004519		

DB913764		gi|54470159|gb|CV570043.1|CV570043	2.00E-25	88.6	jnk sapk-associated		Pfam-B_719	
DB913779		gi|21765084|gb|BQ640625.1|BQ640625	2.00E-19	59.3	map-kinase activating death domain protein denn aex-3			
DB913787	Positive selectior	gi|62843453|gb|DN873512.1|DN873512	0	411	nadh dehydrogenase iron-sulfur proteinmitochondrial	F:GO:0009055,F:GO:0051287	Complex1_49kDa	2.6864
DB913792		gi|57595939|gb|CX568910.1|CX568910	9.00E-34	144	kiaa1738 protein		Pfam-B_4326, Pfam-B_4326, Pfam-B_363	0.85265
DB913797		gi|10661657|gb|BE986879.1|BE986879	3.00E-39	114	homolog dnj-2 precursor		DnaJ,Pfam-B_9221	
DB913798		gi|37635654|gb|CF739317.1|CF739317	0	173	tbc1 domainmember 13	F:GO:0005515	TBC	1.6534
DB913811		gi|19402955|gb|BM943092.1|BM943092	1.00E-06	50.1	---NA---		Peptidase_M13	
DB913827		GS020635	0.003	31.8				
DB913833		gi|62021352|gb|DN679501.1|DN679501	8.00E-09	61.6	novel proteinhuman and mouse amyotrophic lateral sclerosis 2chromosomecandidate 13			
DB913843		BY796572	0.002	40.1				
DB913845		gi|22704007|gb|BU190023.1|BU190023	0	188	u2-associated sr140 protein 1	P:GO:0006396,F:GO:0003676,F:GO:0000166	CTK3, Surp	
DB913848		gi|19118874|gb|BM802051.1|BM802051	0.007	44.1				
DB913861		gi|62046494|gb|DN691780.1|DN691780	2.00E-15	83.1	---NA---		DUF1279	
DB913888		BY797846	0.009	27.6				

DB913900		GS013868	0.009	29.9				
DB913902		GS020551	0.0006	34.5			Pfam-B_13263	
DB913907		gi|54474151|gb|CV572013.1|CV572013	1.00E-25	116	cysteinyl-trna synthetasemitochondrial	F:GO:0016874,F:GO:0046872,C:GO:0005739,F:GO:0000166	tRNA-synt_1e	1.1404
DB913915		gi|58178353|gb|CX768000.1|CX768000	1.00E-18	79.4	dihydrolipoamide branched chain transacylase e2	F:GO:0008415,F:GO:0043754,F:GO:0031405,P:GO:0008152,C:GO:0005739,F:GO:0005515	2-oxoacid_dh	
DB913916		gi|24727839|gb|CA393839.1|CA393839	4.00E-06	48.7	tpr domain containing protein			
DB913919		gi|49672429|gb|CO426135.1|CO426135	0	364	muscle glycogen phosphorylase		Phosphorylase	
DB913929		gi|8487195|gb|BE096264.1|BE096264	5.00E-12	72.1	chromosome 9 open reading frame 30 isoform 2		Pfam-B_489	
DB913930		gi|62039806|gb|DN688475.1|DN688475	0.002	46.1				
DB913934		gi|62872274|gb|DN887511.1|DN887511	0.0007	44.6	---NA---		Pfam-B_4326	
DB913937		gi|24731276|gb|CA395630.1|CA395630	0.002	46.1			Baculo_11_kDa	

DB913946		BY797261	0.004	37.3	immunoglobulin a1 protease	C:GO:0009986,F:GO:0004222,C:GO:0016021,C:GO:0016020,F:GO:0016787,F:GO:0008270,F:GO:0008237,C:GO:0005576,P:GO:0006508,F:GO:0008233,C:GO:0005618,P:GO:0009405,F:GO:0046872		
DB913952		gi|19353745|gb|BM903889.1|BM903889	0	360	phosphoribosyl pyrophosphate synthetase 1b	F:GO:0046872,P:GO:0009156,F:GO:0004749,P:GO:0009116	Pribosyltran	
DB913956	Positive selectior	gi|19030465|gb|BM717207.1|BM717207	0	221	trafficking protein particle complex 4	P:GO:0006888,C:GO:0005783,C:GO:0005801	Sybindin	
DB913957	Positive selectior	gi|57592559|gb|CX565530.1|CX565530	0	357	cytohesin 1	P:GO:0032012,C:GO:0005622	Sec7,Pfam-B_2207	2.74005
DB913964		gi|49674799|gb|CO428505.1|CO428505	0	184	---NA---		DAGK_acc	2.81749
DB913966		gi|29528388|gb|CB584937.1|CB584937	0.009	44.1				
DB913969		gi|54480439|gb|CV575146.1|CV575146	2.00E-07	56.1	williams-beuren syndrome critical region protein			
DB913985		GS008211	0.003	30.9				
DB913991		gi|22812006|gb|BU505773.1|BU505773	6.00E-14	70.2	g-protein signalling modulator 2 (ags3-elegans)	F:GO:0005096,F:GO:0005488,P:GO:0007165	GoLoco,GoLoco,GoLoco,GoLoco	1.20823
DB913996		gi|62839638|gb|DN869697.1|DN869697	0.0002	46.9				

DB914000		gi|62031677|gb|DN684377.1|DN684377	3.00E-25	93.6	folate carrier protein		Mito_carr	0.36387
DB914002		BY796360	0.003	40.1				
DB914006		gi|37602996|gb|CF728828.1|CF728828	0.009	39.1				
DB914027		gi|62880674|gb|DN895911.1|DN895911	0.008	44.1				
DB914035		gi|5033755|gb|AI716502.1|AI716502	5.00E-17	88.6	---NA---		DAGK_cat	
DB914059		gi|11004100|gb|AU142579.1|AU142579	1.00E-05	51	cwc22_debhaame: full=pre-mrna-splicing factor cwc22	C:GO:0005681,F:GO:0005488,C:GO:0005737,P:GO:0008380,P:GO:0016070,P:GO:0006397,F:GO:0005515,C:GO:0005634		
DB914061		gi|24721169|gb|CA390255.1|CA390255	0.003	42.8				
DB914076		gi|13104116|gb|BG306589.1|BG306589	0	326	camp-specific phosphodiesterase 4b		PDEase_I	
DB914098		GS004884	0.001	36.2				
DB914103		BY796077	7.00E-35	142	agap006755-pa isoform 1		U1snRNP70_N,RRM_1	1.51713
DB914109		gi|22681849|gb|BU167865.1|BU167865	0	326	interleukin enhancer binding factor45kda		DZF	
DB914123		gi|57590120|gb|CX563091.1|CX563091	7.00E-38	130	vacuolar protein sorting protein 18	P:GO:0015721,P:GO:0045176,P:GO:0008333,P:GO:0043485,P:GO:0048069,C:GO:0044464,P:GO:0007634	Pep3_Vps18	

DB914124		gi|37598991|gb|CF724823.1|CF724823	7.00E-10	65.2	espin-like protein	F:GO:0005515,C:GO:0015629,P:GO:0007010,C:GO:0042995	Ank,Ank	
DB914127		gi|37600304|gb|CF726136.1|CF726136	5.00E-31	79	heterochromatin proteinbinding protein 3	C:GO:0005694,C:GO:0000786,:GO:0006334,F:GO:0003677,C:GO:0005634	Linker_histone,Linker_histone	
DB914129		gi|126523681|gb|EL647400.1|EL647400	0	314	elongation factor-1 gamma	P:GO:0006414,F:GO:0003746,C:GO:0005853	GST_C,GST_N	1.29333
DB914152		gi|19045875|gb|BM724544.1|BM724544	0.007	44.1				
DB914155		gi|54479595|gb|CV574729.1|CV574729	6.00E-13	74.8	zinc transporter		Zip	
DB914171		gi|10596854|gb|BE954383.1|BE954383	0.006	41.8				
DB914180		BY795432	0.0009	34.1				
DB914187		gi|24559062|gb|CA340966.1|CA340966	0.0002	50.1				
DB914192		gi|62037319|gb|DN687221.1|DN687221	4.00E-10	66.1	dentin sialophosphoprotein			
DB914196		gi|41337670|gb|CK616784.1|CK616784	0	219	coatomer proteinsubunit beta 2 (beta prime)	P:GO:0006461,F:GO:0005515,C:GO:0030117,F:GO:0008565,P:GO:0016192,P:GO:0006886,F:GO:0005198	Coatomer_WDAD	1.30895
DB914210		BY798374	0.0002	35.9				
DB914212	Positive selectio	gi|49677750|gb|CO431456.1|CO431456	1.00E-06	54.7	cg1105 cg1105-pa	F:GO:0003674,P:GO:0008150	Arrestin_N,Arrestin_C	2.46215
DB914229		gi|19399931|gb|BM940779.1|BM940779	0.01	44.1				

DB914236	Positive selectior	gi|75844134|gb|DT874394.1|DT874394	0	340	trafficking protein particle complex 3		TRAPP	3.0934
DB914244		gi|13298246|gb|BG404798.1|BG404798	4.00E-33	142	abc1 family protein		ABC1	0.64775
DB914248		gi|19041815|gb|BM721716.1|BM721716	0.002	44.1	rhotekin 2	P:GO:0007165,C:GO:0005622	PH	
DB914253		gi|18988555|gb|BM678659.1|BM678659	0.0007	46.1				
DB914269		gi|37638272|gb|CF741933.1|CF741933	0	437	dna directed rna polymerase ii polypeptide b	P:GO:0002119,F:GO:0003899,P:GO:0006350,P:GO:0040010,P:GO:0000003,P:GO:0009792,P:GO:0008340	RNA_pol_Rpb2_7,RNA_pol_Rpb2_6	
DB914272		GS020498	0.0003	38.2				
DB914274	Positive selectior	gi|46814587|gb|CN501963.1|CN501963	9.00E-43	174	rho gdp dissociation		Rho_GDI	2.2603
DB914275		BY799992	0.008	35.9				
DB914278		gi|85180004|gb|DW598666.1|DW598666	0	208	fyve finger-containing phosphoinositide kinase (1-phosphatidylinositol-4-phosphate 5-kinase)(phosphatidylinositol-3-phosphate 5-kinase type iii)(nsp-5-kinase) isoform 1		PIP5K	
DB914296		gi|46566690|gb|CN485186.1|CN485186	2.00E-34	112	elongation factor tu (ef-tu)		GTP_EFTU_D3	0.8134
DB914300		GS020635	0.002	31.8				

DB914308		gi|62045584|gb|DN691323.1|DN691323	1.00E-12	74.4	amyloid precursor protein	F:GO:0005488,C:GO:0016021,F:GO:0004867	APP_amyloid, Pfam-B_16930, Pfam-B_1037	0.4179
DB914314		BY798122	7.00E-24	105	mediator complex subunit 11 cg6884-pa		Med11	0.48202
DB914322		BY797251	0.008	29				
DB914347		gi|11003833|gb|AU142312.1|AU142312	2.00E-25	100	required for meiotic nuclear division 5 homolog a		Pfam-B_49, DUF869	1.31703
DB914362		gi|10205227|gb|BE784029.1|BE784029	0	292	af484088_1syntaxin 1a	C:GO:0016021,F:GO:0005484,P:GO:0016192,P:GO:0006886,P:GO:0006836	Syntaxin,SNARE	
DB914365		gi|41342595|gb|CK621709.1|CK621709	3.00E-22	105	c-jun protein	F:GO:0003677,P:GO:0046686,P:GO:0051597	Jun	1.57635
DB914372		gi|62828798|gb|DN859149.1|DN859149	2.00E-30	133	cytochrome c oxidase subunit via polypeptide 1		COX6A	
DB914374		BY795858	1.00E-27	117	ornithine decarboxylase antizyme	F:GO:0008073	ODC_AZ	0.85583
DB914380		gi|40321099|gb|CK355167.1|CK355167	0.002	33.1				
DB914393		gi|19016174|gb|BM702916.1|BM702916	3.00E-15	82.2	calcyclin binding protein	C:GO:0005737,P:GO:0006512,C:GO:0005634	SGS	
DB914415		gi|40319267|gb|CK353380.1|CK353380	0.001	25.4				
DB914418		gi|54440189|gb|CV556048.1|CV556048	2.00E-07	57	src kinase associated phosphoprotein 1	C:GO:0005737,C:GO:0016020,P:GO:0006955,C:GO:0005634	SH3_2	

DB914431		gi|32465039|gb|CD806213.1|CD806213	0.002	43.7				
DB914445		gi|62880016|gb|DN895253.1|DN895253	5.00E-26	75.2	beta chain spectrin		Pfam-B_1882, PH	1.4125
DB914464		gi|24727633|gb|CA393729.1|CA393729	2.00E-17	90.4	lymphoid-restricted membraneisoform cra_a		MRVI1	
DB914494		gi|49674086|gb|CO427792.1|CO427792	2.00E-20	100	mediator of rna polymerase ii transcription	P:GO:0009987	Med24_N	0.5669
DB914496		gi|22810544|gb|BU504311.1|BU504311	0.001	35.4				
DB914502		gi|16086838|gb|BI879567.1|BI879567	0.005	31.3	transposase		Pfam-B_19958	1.2465
DB914521		gi|19044026|gb|BM723028.1|BM723028	9.00E-05	47.8	set domain and mariner transposase fusion	P:GO:0009987,F:GO:0003824		
DB914525		gi|18523633|gb|BM474591.1|BM474591	7.00E-10	64.3	pentatricopeptide repeat protein 1			
DB914526		gi|37643689|gb|CF747346.1|CF747346	0.0007	48.1				
DB914527		gi|29773470|gb|CB716322.1|CB716322	0.01	44.1				
DB914529		gi|58032704|gb|CX730231.1|CX730231	5.00E-21	102	agrin_disomame: full=agrin		Pfam-B_6679, EGF ,Laminin_G_2	
DB914543		gi|10661640|gb|BE986871.1|BE986871	0.0007	48.1				
DB914548		gi|9126593|gb|BE256137.1|BE256137	0.009	35.9				

DB914550		gi|16491993|gb|BB658168.1|BB658168	0.003	46.1				
DB914606		gi|41342768|gb|CK621882.1|CK621882	0	263	calcium calmodulin-dependent protein kinaseii delta	F:GO:0004683,P:GO:0006468,F:GO:0005524,F:GO:0005516	CaMKII_AD	1.79304
DB914609		gi|49680679|gb|CO434385.1|CO434385	0.009	44.1	kiaa0523 protein		WSC	
DB914611		gi|37634173|gb|CF737836.1|CF737836	8.00E-28	59.7	integrin alpha fg-gap repeat containing 1	F:GO:0005515	Pfam-B_10882,FG-GAP,Pfam-B_2701	1.5911
DB914618		gi|85175473|gb|DW597555.1|DW597555	0.006	35.9				
DB914626	Positive selectior	gi|13269951|gb|BG373449.1|BG373449	0	277	rna-binding protein luc7-like 1		LUC7	2.96008
DB914647		GS013972	0.006	34.2				
DB914665		gi|19018339|gb|BM705081.1|BM705081	0.0004	45.9	glutamic acid-rich protein cnbl1500		IQ	
DB914701		GS004835	0.006	34.2				
DB914704		gi|19045114|gb|BM723783.1|BM723783	0	263	voltage and ligand gated potassium channel		cNMP_binding	
DB914711		gi|16045694|gb|BI872019.1|BI872019		0	aspartate aminotransferase	P:GO:0008152,F:GO:0008483	Aminotran_1_2	1.94034
DB914715		gi|15708152|gb|BI731139.1|BI731139	0	371	plasma membrane calcium atpase		Hydrolase_3,Cation_ATPase_C	
DB914741		BY798376	0.009	36.1	---NA---			
DB914754		gi|13298781|gb|BG374309.1|BG374309	0.008	40.9				

DB914755		gi|62022742|gb|DN680198.1|DN680198	0	248	receptor-type tyrosine-protein phosphatase n2		Y_phosphatase	0.0223
DB914756		GS020551	0.006	34.2				
DB914758		gi|14975170|gb|BI298890.1|BI298890	0.01	44.1				
DB914759		BY797937	5.00E-05	46.1				
DB914768		gi|18988103|gb|BM678207.1|BM678207	1.00E-13	77.6	loc779536 protein			
DB914775		gi|83525008|gb|DB294182.1|DB294182	0.002	46.1				
DB914777		gi|75840093|gb|DT870353.1|DT870353	5.00E-34	145	cytochrome c oxidase subunit iv	F:GO:0004129,P:GO:0006118	COX4	1.82462
DB914790		gi|22811777|gb|BU505544.1|BU505544	5.04E-44	110	heterogeneous nuclear ribonucleoprotein l	F:GO:0005488		1.66068
DB914792		gi|49675509|gb|CO429215.1|CO429215	0	347	udp-n-acetyl-alpha-d-galactosamine:polypeptide n-acetylgalactosaminyltransferase 13	F:GO:0004653,F:GO:0046872,F:GO:0043169,P:GO:0006493,C:GO:0016020	Ricin_B_lectin, Ricin_B_lectin	0.24575
DB914798		gi|55687806|gb|CV782866.1|CV782866	9.00E-08	58.3	protocadherin gamma subfamily3 isoform 1 precursor isoform 4		Cadherin,Cadherin	
DB914800		gi|62838255|gb|DN868314.1|DN868314	0.001	33.1	---NA---		Pfam-B_4326, Pfam-B_4326,Pfam-B_4326	
DB914805		gi|49670616|gb|CO424453.1|CO424453	2.00E-16	83.1	dead box polypeptide 42 protein	F:GO:0003723,F:GO:0005524,C:GO:0005634,F:GO:0008026		
DB914807		gi|50135734|gb|CO470191.1|CO470191	6.00E-14	78	tubulin-specific chaperone d			
DB914811		GS020613	0.006	34.2				

DB914825	Embryo specific	GS014020	0.005	34.2				
DB914830		GS020737	9.00E-05	40.1				
DB914835		BY798120	0.004	40.1				
DB914837		gi|37633333|gb|CF736997.1|CF736997	0	290	component of oligomeric golgi complex 6P:GO:0015031,C:GO:0000139,C:GO:0017119		COG6	
DB914853		gi|75833992|gb|DT864252.1|DT864252	5.00E-07	55.1	ubiquinol-cytochrome c reductase subunit-like		QCR10,UCR_6-4kD	
DB914855		gi|19042411|gb|BM722079.1|BM722079	2.00E-10	66.6	zinc finger protein 271 (zinc finger protein 7) (zinc finger protein znfphex133) (epstein-barr virus-induced zinc finger protein) (znf-eb) (ct-zfp48) (zinc finger protein dp) (znf-dp)	F:GO:0003676,F:GO:0008270,C:GO:0005622	zf-C2H2	
DB914862		BY795675	0	213	epsilon subunit of coatomer protein complex isoform 1	P:GO:0016192,F:GO:0005488,C:GO:0030137,P:GO:0016043,C:GO:0044425,C:GO:0031090,C:GO:0012505	Coatomer_E	1.50606
DB914867		gi|46821517|gb|CN508893.1|CN508893	1.00E-14	81.3	tumor protein d52-like 2b		TPD52	
DB914886		gi|37602491|gb|CF728323.1|CF728323	0	229	liquid facets		Pfam-B_15358,ENTH	2.05756
DB914895		gi|22347977|gb|BQ932594.1|BQ932594	1.40E-45	139	cop9 signalosome complex subunit		PCI	0.26647
DB914897		BY798777	0.003	40.1				

DB914898		gi|119785924|gb|EH279915.1|EH279915	2.00E-27	123	selenoprotein w	F:GO:0008430,C:GO:0005737,F:GO:0016491,C:GO:0005886	Rdx	0.93773
DB914905		gi|32173367|gb|CD671636.1|CD671636	0	186	sulfatase-modifying factor 1 precursor		FGE-sulfatase	0.60223
DB914917		GS013972	0.006	34.2				
DB914919	GO annotation	gi|58032883|gb|CX730410.1|CX730410	2.00E-22	102	rhodopsin kinase	P:GO:0006468,F:GO:0004871,F:GO:0005524,P:GO:0007165,F:GO:0004703		
DB914921		gi|49672103|gb|CO425809.1|CO425809	0	331	syndecan bindingisoform cra_a	C:GO:0005895,F:GO:0016491,F:GO:0005137,C:GO:0042470,F:GO:0047485,P:GO:0008152,F:GO:0046982,P:GO:0007265,F:GO:0042043	PDZ, PDZ	1.00847
DB914924		gi|58032634|gb|CX730161.1|CX730161	2.00E-13	76.7	atp-dependent bile acid permease		ABC_membrane	1.0399
DB914927		gi|62827356|gb|DN857707.1|DN857707	0	225	mitochondrial ribosomal	C:GO:0005622		1.58155
DB914952		gi|46464646|gb|CN458920.1|CN458920	5.00E-34	75.3	probable rna-binding protein 19		RRM_1, Pfam-B_1057,RRM_1	1.00403
DB914954	Positive selectior	gi|46819175|gb|CN506551.1|CN506551	0	442	ribosome biogenesis protein nsa2 homolog		Pfam-B_11758,Ribosomal_S8e	3.08143
DB914957		BY797925	0.005	36.8				
DB914959		gi|9124583|gb|BE254157.1|BE254157	4.00E-05	52				
DB914968		gi|50136911|gb|CO470735.1|CO470735	3.00E-26	76.7	transmembrane protein 50a		UPF0220,UPF0220	0.90197

DB914971		gi|10204034|gb|BE782836.1|BE782836	0.0004	36.8				
DB914981		GS004685	0.009	32.2				
DB915001		gi|40678063|gb|CK430204.1|CK430204	6.00E-11	61.6	peptidylglycine alpha-amidating monooxygenase	F:GO:0004504,P:GO:0006518,P:GO:0009987,F:GO:0005507,F:GO:0003824,C:GO:0016020,F:GO:0004497,F:GO:0004867	NHL	0.6148
DB915010		gi|62041170|gb|DN689160.1|DN689160	0	201	atp-dependent transporter		Pfam-B_11162,Pfam-B_11182,ABC_tran	
DB915022	**Embryo specific**	BY797574	0.007	38.2				
DB915040		gi|37630580|gb|CF734247.1|CF734247	0	152	domain containing 1			1.2712
DB915046	**Embryo specific**	gi|50134930|gb|CO469857.1|CO469857	5.00E-05	52				
DB915065		gi|85187158|gb|DW601144.1|DW601144	0.0001	40.9	---NA---			
DB915089		gi|62842970|gb|DN873029.1|DN873029	0.006	33.6	orcokinin precursor	P:GO:0007218,F:GO:0005184,C:GO:0005576		
DB915090		gi|22689959|gb|BU175975.1|BU175975	0.001	40.6				
DB915094		GS020443	0.006	34.2	pdx1_subdoame: full=probable pyridoxine biosynthesis snzerrame: full=pdx1 homologame: full=ethylene response protein	P:GO:0008615,F:GO:0003824	SOR_SNZ	0.38215

DB915096		GS020567	0.006	30.9			
DB915118		gi|32176841|gb|CD675110.1|CD675110	2.00E-10	66.9	subfamilymember 1	F:GO:0051082,P:GO:0006457,P:GO:0045449,F:GO:0031072,F:GO:0003677,C:GO:0005634	Myb_DNA-binding	
DB915122		GS004800	0.001	36.2				
DB915128		gi|75842167|gb|DT872427.1|DT872427	0	204	protein		eIF-1a	0.30832
DB915140		gi|32273244|gb|CD722396.1|CD722396	9.00E-36	151	---NA---		PHD, Bromodomain, PHD	
DB915141		GS013851	0.006	34.2				
DB915148		GS004600	0.006	34.2				
DB915151		BY795161	0.006	36.2				
DB915153		gi|62826040|gb|DN856391.1|DN856391	0	318	myst histone		MOZ_SAS,Pfam-B_7698, MOZ_SAS	
DB915164		gi|32464819|gb|CD805993.1|CD805993	0	386	glycogen brain	F:GO:0030170,F:GO:0004645	Phosphorylase	0.04362
DB915168		gi|68325747|gb|DR423731.1|DR423731	0	274	ribosomal protein s11	C:GO:0005840,F:GO:0003735,P:GO:0006412	Ribosomal_S17	
DB915191		GS008298	0.01	30.4				
DB915213		GS020603	0.006	34.2				
DB915221		BY796397	0.004	40.1	---NA---		Aminotran_5	
DB915228		gi|18500152|gb|BM451112.1|BM451112	2.00E-11	51.5	pap2 superfamily protein	F:GO:0016787,F:GO:0003824,C:GO:0016020	PAP2	
DB915231		BY797899	0.009	36.2				
DB915236		GS020630	0.006	34.2				
DB915244		gi|22678666|gb|BU164714.1|BU164714	0	240	orm1-like 1	C:GO:0005783,C:GO:0044425	ORMDL	
DB915246		BY799230	0.003	40.1				

DB915252		gi|20362619|gb|BQ187068.1|BQ187068	5.00E-14	57.4	core-fucosyltransferase a	F:GO:0016757,C:GO:0016020	Glyco_transf_10	0.69855
DB915290		gi|50138615|gb|CO471538.1|CO471538	0	292	rap guanine nucleotide exchange factor4		RasGEF,Sec7	
DB915313		BY798198	0.003	36.2				
DB915332		gi|46818123|gb|CN505499.1|CN505499	5.00E-28	108	atp synthase lipid-bindingmitochondrial precursor	P:GO:0015986,C:GO:0045263,C:GO:0016021,C:GO:0016020,C:GO:0016469,P:GO:0006811,P:GO:0006810,F:GO:0015078,F:GO:0046933,F:GO:0046961,F:GO:0008289,P:GO:0015992	ATP-synt_C	
DB915334	Positive selectior	gi|18521031|gb|BM471989.1|BM471989	0	474	gtp binding protein 4	F:GO:0005525	NOG1	12.4053
DB915355		gi|46814772|gb|CN502148.1|CN502148	2.00E-07	57	protein		Pfam-B_9880,Pfam-B_18588,Pfam-B_7249	
DB915358		gi|32466998|gb|CD808172.1|CD808172	5.00E-06	44	remodeling and spacing factor 1			
DB915364		GS004835	0.006	34.2				
DB915366		gi|85164424|gb|DW592739.1|DW592739	0	185	non-erythrocytic 1 (alpha-fodrin)	C:GO:0005737,C:GO:0005856,P:GO:0051016,F:GO:0003779,F:GO:0005509,F:GO:0005516	efhand_Ca_insen,efhand	0.76977
DB915381		gi|37630736|gb|CF734403.1|CF734403	3.00E-15	61.6	nicotinic acetylcholine receptor subunit type i	F:GO:0004872,F:GO:0005230,P:GO:0006810,C:GO:0044425,C:GO:0045202	Neur_chan_memb	

DB915390		gi|62878361|gb|DN893598.1|DN893598	2.00E-40	166	gtp binding protein 4	F:GO:0005525,P:GO:0007264	Pfam-B_1475,NOGCT	
DB915398		GS020721	0.006	34.2				
DB915414		gi|85204141|gb|DW606709.1|DW606709	3.00E-27	110	wd repeat domain 59		Pfam-B_1901	1.06237
DB915434	Positive selectior	gi|62879067|gb|DN894304.1|DN894304	0	195	hydroxyacyl-coenzyme a dehydrogenase 3-ketoacyl-coenzyme a thiolase enoyl-coenzyme a hydratase (trifunctional protein) beta subunit	C:GO:0005743,P:GO:0006631,F:GO:0003988	Thiolase_N,Thiolase_N	2.54372
DB915444		gi|5210701|gb|AI764766.1|AI764766	3.00E-21	84.9	---NA---		Pfam-B_4326,Na_Ca_ex	
DB915489		gi|27167682|gb|BY742285.1|BY742285	1.00E-10	68	ceramide kinase-like protein		DAGK_cat	
DB915490		gi|22695415|gb|BU181431.1|BU181431	0.0007	48.1				
DB915494		gi|22667226|gb|BU153704.1|BU153704	0	361	exosome componentisoform cra_a		RNase_PH_C, RNase_PH	0.10495
DB915500		gi|54446259|gb|CV559043.1|CV559043	8.00E-16	84.9	kinesin-73 cg8183-pb		CAP_GLY	
DB915516		gi|83528675|gb|DB294632.1|DB294632	0.009	44.1				
DB915518		BY799694	0.004	40.1				
DB915531		gi|9122948|gb|BE252803.1|BE252803	0	215	trafficking protein particle complex 2	P:GO:0006888,C:GO:0005622	Sedlin_N	

DB915536		gi|18516245|gb|BM467203.1|BM467203	2.00E-14	50.6	n-acetylglucosaminyltransferase v	F:GO:0016740,C:GO:0005575		
DB915540		gi|24726160|gb|CA392957.1|CA392957	1.00E-08	61.1	sodium-iodide related cotransporter			1.33033
DB915561		GS013876	0.001	34.2				
DB915569		GS008202	0.0004	38.2	reverse transcriptase	F:GO:0003723,P:GO:0006278,F:GO:0003964		0.63493
DB915570		gi|46816392|gb|CN503768.1|CN503768	0	176	rna binding motif protein 8a	C:GO:0044424,F:GO:0005488	RRM_1	0.27304
DB915574		gi|32180984|gb|CD679253.1|CD679253	0.009	44.1				
DB915596		gi|50134954|gb|CO469865.1|CO469865	3.00E-26	118	ntf2-related export protein 2		Pfam-B_5119,NTF2	0.78157
DB915607		gi|62033613|gb|DN685351.1|DN685351	0	264	novel proteintransmembrane protein 111 (zgc:86609)	F:GO:0003674,C:GO:0005575	DUF106	0.13216
DB915615		GS020607	0.005	34.2				
DB915617		GS014020	0.005	34.2				
DB915644		GS004966	0.006	34.2				
DB915667		GS020551	0.003	31.8				
DB915668		gi|37629295|gb|CF732962.1|CF732962	4.00E-15	82.6	pb1 protein		BAH	
DB915670		gi|46855352|gb|CN527196.1|CN527196	5.00E-06	51.9	rab11 family interacting protein 2 (class i)		RBD-FIP	
DB915675		gi|54441692|gb|CV556796.1|CV556796	0	286	basic leucine zipper and w2 domains 1	P:GO:0006353,F:GO:0005488,P:GO:0045449,P:GO:0032268,P:GO:0051128	W2	0.70233
DB915689		gi|10666276|gb|BE989171.1|BE989171	0.01	44.1				

DB915691		gi|24723586|gb|CA391579.1|CA391579	2.00E-07	49.6	g protein gamma subunit		G-gamma	0.92647
DB915693		gi|58060845|gb|CX734009.1|CX734009	3.00E-37	155	protein kinasedelta	F:GO:0005488,F:GO:0016301	Pfam-B_9526,C1_1,C1_1	
DB915719		BY796848	0.006	36.2				
DB915750		gi|19001128|gb|BM687870.1|BM687870	5.61E-45	181	chromosome 14 open reading frame 135	C:GO:0016021	Pecanex_C	
DB915757		gi|62871793|gb|DN887030.1|DN887030	2.00E-06	43.7	prothoracicostatic peptide precursor	P:GO:0006355,F:GO:0005179,F:GO:0003674,F:GO:0000166,C:GO:0005615,P:GO:0002168,C:GO:0005576		
DB915761		gi|19018444|gb|BM705186.1|BM705186	8.00E-11	38.6	phospholipase a2	F:GO:0046872,F:GO:0004623,F:GO:0005509,F:GO:0016787,P:GO:0016042,C:GO:0005576,P:GO:0006644	Phospholip_A2_1	0.77686
DB915763		gi|22667808|gb|BU154276.1|BU154276	0	362	glyceraldehyde-3-phosphate dehydrogenase	C:GO:0005737,F:GO:0051287,P:GO:0006096,F:GO:0004365	Gp_dh_C,Gp_dh_N	0.10068
DB915774		gi|75846277|gb|DT876537.1|DT876537	5.00E-40	165	light-inducedbrain protein 44		UPF0041	1.14919
DB915776		gi|62826866|gb|DN857217.1|DN857217	0	445	casein kinasebeta polypeptide isoform 2	P:GO:0030177,P:GO:0007249,F:GO:0005515,C:GO:0005956,F:GO:0004682,F:GO:0008605	CK_II_beta,Pfam-B_5275	1.3143
DB915789		GS013908	0.005	34.2				
DB915807		gi|41349257|gb|CK628371.1|CK628371	0.001	46.1				
DB915812		GS020701	0.002	32.2				

DB915822		BY798436	0.007	36.3				
DB915832		gi|16086844|gb|BI879573.1|BI879573	0.0001	47.8	---NA---			
DB915848		GS014157	0.005	34.2				
DB915849		gi|22679229|gb|BU165277.1|BU165277	3.00E-32	139	translocated promoter region (to activated met oncogene)	F:GO:0003674,C:GO:0005635	Cortex-I_coil	0.90314
DB915858		BY797553	0.0009	39.1				
DB915864		gi|14064158|gb|BG753505.1|BG753505	0.009	31.3				
DB915866		gi|18510625|gb|BM461585.1|BM461585	0	456	histone deacetylase 3		Hist_deacetyl	2.44999
DB915868		gi|22355575|gb|BQ940097.1|BQ940097	5.00E-05	45	pleckstrin homology domainfamily m (with run domain) member 2			
DB915882		gi|85165194|gb|DW593133.1|DW593133	3.00E-09	51.3	phd finger protein 8			
DB915883		gi|15959381|gb|BI846858.1|BI846858	4.00E-07	55.8	map microtubule affinity-regulating kinase 1	P:GO:0009987,F:GO:0016740,C:GO:0044424		1.96025
DB915891		gi|49672802|gb|CO426508.1|CO426508	5.00E-39	161	---NA---		Sdh5	0.84475
DB915905		gi|37599559|gb|CF725391.1|CF725391	0.003	46.1				
DB915914		gi|46824907|gb|CN512283.1|CN512283	0	96.9	phosphatidylinositol transfer protein retinal degeneration b protein		IP_trans	
DB915917		BY797191	0.004	36.2				
DB915922		gi|22803777|gb|BU501356.1|BU501356	8.00E-14	57.4	ras and ef-hand domain containing	P:GO:0009987,P:GO:0006810	PglZ, Ras	0.3492

DB915936		gi|54445268|gb|CV558564.1|CV558564	5.00E-20	47.8	pentatricopeptide repeat domain 3			
DB915948		GS008053	0.006	32.2				
DB915966		gi|49673531|gb|CO427237.1|CO427237	2.00E-28	126	propionyl-coenzyme aalpha polypeptide	F:GO:0005524,F:GO:0016874,P:GO:0009102,P:GO:0008152,F:GO:0003824,F:GO:0009374,C:GO:0005575	Biotin_carb_C	
DB915971		gi|85185754|gb|DW600651.1|DW600651	0	131	mtp18_danreame: full=mitochondrial 18 kda proteiname: full=mtp18	P:GO:0006915,C:GO:0005739,F:GO:0008270,F:GO:0004089,P:GO:0015976	MTP18	
DB915990		gi|75841529|gb|DT871789.1|DT871789	0	212	nuclear transcription factorbeta	C:GO:0005622	CBFD_NFYB_HMF, Pfam-B_5136	0.08562
DB915991		gi|22803689|gb|BU501318.1|BU501318	4.00E-12	72.5	cg12393 cg12393-pa		PH	
DB915994		GS008140	0.006	34.2				
DB916009		gi|85168246|gb|DW594662.1|DW594662	3.00E-22	103	sulfide:quinone oxidoreductase			
DB916010		gi|62839787|gb|DN869846.1|DN869846	0.0002	50.1				
DB916027		GS020504	0.006	34.2				
DB916040		GS014173	0.005	34.2				
DB916042		gi|46820184|gb|CN507560.1|CN507560	0	361	dehydrodolichyl diphosphate synthase	F:GO:0016740,C:GO:0005575	Prenyltransf	0.8832
DB916052		GS020629	0.001	36.2				
DB916059		gi|46815093|gb|CN502469.1|CN502469	0	342	isocitrate dehydrogenase 2 (nadp+)mitochondrial	P:GO:0008152,F:GO:0004450	Iso_dh	1.35172

DB916069		gi|40542491|gb|BU694178.1|BU694178	0.003	46.1				
DB916103		BY798920	0.0002	44.1				
DB916106		BY799441	0.004	40.1				
DB916112		BY795187	0	226	ribosomal protein l27		Ribosomal_L27e	
DB916114		gi|19401994|gb|BM942131.1|BM942131	0.003	46.1			Pfam-B_9429, Pfam-B_2280,Pfam-B_19169	
DB916138		gi|62889609|gb|DN904846.1|DN904846	0.009	40.1				
DB916143		gi|40318253|gb|CK352366.1|CK352366	0.003	46.1				
DB916147		GS004664	0.002	36.2				
DB916154		gi|37635939|gb|CF739601.1|CF739601	0	201	histone h2b-3	C:GO:0000786,F:GO:0003677,P:GO:0006334,C:GO:0005634	Histone	1.67955
DB916156		gi|40319267|gb|CK353380.1|CK353380	0.009	37.3				
DB916160		gi|19399078|gb|BM939926.1|BM939926	0.005	42.3				
DB916166		gi|23650882|gb|BU728721.1|BU728721	0.003	46.1				
DB916182		GS013860	0.006	34.2				
DB916190		gi|62041668|gb|DN689395.1|DN689395	4.00E-29	107	lmbr1 domain-containing			0.9933

DB916201		gi|62832587|gb|DN862938.1|DN862938	4.00E-37	155	ring finger protein 115	F:GO:0046872,F:GO:0008270,F:GO:0005515	Pfam-B_13390,Pfam-B_2600,Pfam-B_14559,Pfam-B_9059	1.1331
DB916209		gi|62853764|gb|DN883809.1|DN883809	0.004	42.8				
DB916211		GS004800	0.002	36.2				
DB916225		GS004616	0.006	34.2				
DB916242		gi|57593145|gb|CX566116.1|CX566116	0	111	meningioma expressed antigen 5	F:GO:0016787,F:GO:0016740	Pfam-B_600,Pfam-B_600	0.37438
DB916251		GS008258	0.006	34.2				
DB916276		gi|62032998|gb|DN685038.1|DN685038	0.004	37.7				
DB916277		gi|54439969|gb|CV555940.1|CV555940	5.00E-06	52.4	---NA---		Pfam-B_4326, Pfam-B_4326	
DB916280		gi|54447646|gb|CV559734.1|CV559734	4.00E-31	112	kelch domain containing 10	F:GO:0003674,P:GO:0008150	Kelch_1, Kelch_2	
DB916283		gi|62872736|gb|DN887973.1|DN887973	0	355	chaperonin containingsubunit 7	P:GO:0006457,F:GO:0051082,F:GO:0005524,F:GO:0042802,C:GO:0005832	Cpn60_TCP1	2.6597
DB916293		gi|24025296|gb|BU840896.1|BU840896	0.004	38.6				
DB916298		BY800055	0.0009	42.1				
DB916304		gi|62883081|gb|DN898318.1|DN898318	1.00E-09	64.3	organic cation transporter family member (oct-1)	F:GO:0015101,P:GO:0015695,C:GO:0016021	Sugar_tr	1.1984
DB916307		gi|24721459|gb|CA390418.1|CA390418	7.00E-05	48.7	neuroendocrine protein 7b2		Secretogranin_V	1.3485

DB916330		gi|13148998|gb|BG342560.1|BG342560	1.00E-06	53.8	cell adhesion molecule		Pfam-B_2000,fn3	1.6787
DB916334		gi|62884905|gb|DN900142.1|DN900142	0.002	30.4				
DB916350		gi|18973569|gb|BM666120.1|BM666120	0.0003	44.6			Pfam-B_4326	
DB916353		gi|15715666|gb|BI738653.1|BI738653	2.00E-42	158	progesterone receptor membrane component 1		Pfam-B_3152,Cyt-b5	
DB916355		gi|32274839|gb|CD723985.1|CD723985	0.003	46.1				
DB916366		gi|16779712|gb|BM050445.1|BM050445	8.00E-08	44.6	multisynthetase complex p38 auxiliary component		HPC2,Pfam-B_18106,GST_C	
DB916367		gi|14960856|gb|BI296435.1|BI296435	0.003	46.1				
DB916376		gi|21120176|gb|BQ424861.1|BQ424861	7.00E-19	66.3	ephrin receptor	F:GO:0016740	fn3, fn3, Pfam-B_2000, WSC	
DB916378		gi|24719633|gb|CA389458.1|CA389458	5.00E-06	52.4	zinc transporter	P:GO:0006829,F:GO:0005385	Zip	
DB916388		gi|15707388|gb|BI730375.1|BI730375	0.006	28.6				
DB916409		gi|77501868|gb|DV214183.1|DV214183	6.00E-12	72.1	mitochondrial phosphate carrier protein cg4994-pa	C:GO:0005743,C:GO:0016021,F:GO:0005488,P:GO:0006810	Mito_carr	
DB916428	Embryo specific	gi|42746200|gb|CK780522.1|CK780522	3.00E-25	69.2	ded protein		Pfam-B_2199	
DB916446		gi|37635569|gb|CF739232.1|CF739232	0.001	44.1				
DB916454		BY799681	8.00E-14	50.1	---NA---		Pkinase	

DB916465		gi|49672835|gb|CO426541.1|CO426541	2.00E-35	148	brefeldin a-inhibited guanine nucleotide-exchange protein 1			1.0219
DB916467		BY799112	0.004	40.1				
DB916469		gi|62023664|gb|DN680663.1|DN680663	4.00E-19	95.9	prp38 pre-mrna processing factor 38domain		PRP38	0.55547
DB916481		gi|15713843|gb|BI736830.1|BI736830	5.00E-42	128	subfamilymember 9		DnaJ,Pfam-B_4588, Pfam-B_19269,Pfam-B_9394	0.39313
DB916512		gi|62827568|gb|DN857919.1|DN857919	7.00E-13	40.9	resistance to inhibitors of cholinesterase 8 homolog a	C:GO:0005737	Ric8	
DB916514		gi|15713384|gb|BI736371.1|BI736371	6.00E-24	87.2	dopamine receptor-1		7tm_1	0.60642
DB916520		gi|41341993|gb|CK621107.1|CK621107	6.00E-22	105	short chain dehydrogenase	F:GO:0005488,F:GO:0016491,P:GO:0008152,F:GO:0003824	adh_short	2.12755
DB916529		BY799434	0.0009	42.1	neighbor of brca1 gene 1			
DB916541		gi|9382869|gb|BB465680.1|BB465680	0.01	44.1				
DB916545		gi|85184210|gb|DW600145.1|DW600145	3.00E-30	98.2	---NA---		CSD	1.86407
DB916546		gi|32464111|gb|CD805285.1|CD805285	0	246	mitogen-activated protein kinase kinase 1	P:GO:0006468,F:GO:0005524,F:GO:0004674,F:GO:0004713	Pkinase	
DB916547		gi|1088214|gb|H92636.1|H92636	0.0006	48.1	---NA---			
DB916552		GS013863	0.0004	38.2				
DB916575		gi|19042248|gb|BM721984.1|BM721984	2.00E-25	104	pleckstrin homology domainfamily m (with run domain) member 2	C:GO:0016020	PH	

DB916580		gi|13287696|gb|BG394248.1|BG394248	7.00E-34	131	cug tripletrna binding protein 1	F:GO:0003723,C:GO:0005737,F:GO:0000166,P:GO:0006397,C:GO:0005634	Pfam-B_9954, RRM_1	
DB916581		gi|13298115|gb|BG404667.1|BG404667	0	197	trap1 protein	C:GO:0005615,C:GO:0005739,F:GO:0000166	HSP90	0.97455
DB916589		BY799992	0.003	40.1				
DB916598		gi|62040511|gb|DN688828.1|DN688828	0.009	44.1				
DB916602		gi|62828226|gb|DN858577.1|DN858577	1.00E-22	97.7	mitochondrial ribosomal protein s31	F:GO:0003677,C:GO:0005634,C:GO:0005739,P:GO:0006355		1.06517
DB916610		gi|15713449|gb|BI736436.1|BI736436	0	199	ryanodine receptorbrain			
DB916621		gi|24025046|gb|BU840646.1|BU840646	0	260	flj10081 protein			
DB916638	**Positive selectior**	gi|62841191|gb|DN871250.1|DN871250	0	443	glucose-6-phosphate isomerase		PGI	2.77942
DB916649	**Embryo specific**	gi|46565340|gb|CN483836.1|CN483836	9.81E-45	180			DUF618	0.46992
DB916691		gi|23679586|gb|BU740243.1|BU740243	0.003	46.1				
DB916696		gi|22368572|gb|BQ953094.1|BQ953094	0	430	septin-1 (diff6 protein homolog) (protein innocent bystander)		Septin	0.64062
DB916698		GS014086	0.005	34.2				
DB916705		gi|16779962|gb|BM050695.1|BM050695	0.0002	46.9	isoform cra_a	P:GO:0007067,F:GO:0005085,C:GO:0005634,F:GO:0003677		

DB916708		gi|22810996|gb|BU504763.1|BU504763	0.003	46.1				
DB916710		gi|22677816|gb|BU163864.1|BU163864	0	424	---NA---		AdoHcyase_NAD, AdoHcyase	0.61617
DB916716		gi|21119271|gb|BQ423947.1|BQ423947	0	444	solute carrier family 25 member 3 isoform b precursor isoform 5	C:GO:0005743,F:GO:0015317,P:GO:0006810,C:GO:0016021	Mito_carr, Mito_carr, Mito_carr	0.3872
DB916728		gi|49677532|gb|CO431238.1|CO431238	0	345	retinoic acid induced 17		zf-MIZ	3.2364
DB916730		gi|45305774|gb|CK876143.1|CK876143	4.00E-08	53.3	transposable element tcb1 transposase	F:GO:0004803,P:GO:0015074,P:GO:0006313,F:GO:0003677,C:GO:0005634	Pfam-B_261,Transposase_5	
DB916731	Positive selectior	gi|85209685|gb|DW609023.1|DW609023	0	594	splicing factorpartial		Mov34 ,PROCT	2.29367
DB916737		gi|85186909|gb|DW601057.1|DW601057	2.00E-13	77.1	cell growth regulator with ring finger domain 1		Pfam-B_14559	
DB916738		gi|46867777|gb|CN539621.1|CN539621	0	477	protein phosphatase 2 (formerly 2a)regulatory subunit a (pr 65)alpha isoform	F:GO:0005515,C:GO:0005829	HEAT,HEAT,HEAT,HEAT,HEAT	1.53838
DB916747		gi|16498433|gb|BB664679.1|BB664679	1.00E-05	51	limbic system-associated membrane protein		I-set,I-set	
DB916749		gi|37600524|gb|CF726356.1|CF726356	0.0006	36.8	group ii plp decarboxylase			
DB916750		gi|18511152|gb|BM462112.1|BM462112	4.00E-16	86.1	neurofilament protein	C:GO:0005882,F:GO:0005198	Filament,Pfam-B_9207, IncA	0.14146
DB916759	Embryo specific	GS013851	0.006	34.2				

DB916787		gi|21761396|gb|BQ636937.1|BQ636937	3.00E-32	138	briggsae cbr-kpc-1 protein	P:GO:0006508,F:GO:0004289	P_proprotein	1.35643
DB916797		GS020737	0.006	24.9			Toxin_9	
DB916803		gi|5034953|gb|AI717649.1|AI717649	0.01	44.1				
DB916806	Embryo specific	gi|18511097|gb|BM462057.1|BM462057	7.01E-45	156	lipaselysosomalcholesterol esterase	F:GO:0003824	Abhydro_lipase,Abhydrolase_1	
DB916826		gi|62840200|gb|DN870259.1|DN870259	1.00E-28	127	aspartyl-trna synthetase 2	F:GO:0016874,C:GO:0005739,F:GO:0000166	tRNA-synt_2,tRNA-synt_2,tRNA_anti	
DB916834		gi|119789939|gb|EH282497.1|EH282497	0.009	44.1				
DB916841		GS020607	0.003	31.8				
DB916855		gi|19038856|gb|BM719944.1|BM719944	0.009	44.1				
DB916864		gi|62834106|gb|DN864457.1|DN864457	5.00E-11	68.9	transmembrane protein 68	F:GO:0008415,P:GO:0008152,C:GO:0016021,C:GO:0016020		
DB916866		gi|46823056|gb|CN510432.1|CN510432	4.00E-27	122	ras-related protein rab-2			1.18766
DB916884		gi|55687774|gb|CV782834.1|CV782834	7.00E-11	68.4	neuroligin 2	F:GO:0004104,F:GO:0016787	COesterase	
DB916892		gi|21762757|gb|BQ638298.1|BQ638298	0.003	46.1				
DB916896		gi|46867496|gb|CN539340.1|CN539340	6.00E-43	174	cg32486 cg32486-pd			1.59183

DB916905		gi|19043030|gb|BM722448.1|BM722448	1.00E-42	159	myosin heavy chain v	C:GO:0016459,F:GO:0005524,F:GO:0003774	Pfam-B_7363	
DB916916		gi|54449541|gb|CV560673.1|CV560673	0	385	pyruvate kinase	F:GO:0004743,F:GO:0030955,F:GO:0000287,P:GO:0006096	PK_C,PK	3.11525
DB916921	Positive selectior	gi|62886183|gb|DN901420.1|DN901420	5.00E-40	163	14-3-3 zeta		2014/03/03	3.81437
DB916930		gi|57592233|gb|CX565204.1|CX565204	0	346	cop9 (constitutive photomorphogenic) subunit 4 (arabidopsis thaliana)	F:GO:0005515,C:GO:0008180	LppC,RPN7	0.88055
DB916931		gi|24718100|gb|CA388673.1|CA388673	0.001	43.7				
DB916935		gi|62879685|gb|DN894922.1|DN894922	0	213	cathepsin b		Propeptide_C1,Peptidase_C1	1.5871
DB916953		gi|49673021|gb|CO426727.1|CO426727	6.00E-07	55.2	sh2-domain-containing inositol 5-phosphatase 2a	C:GO:0044464	Pfam-B_1233	
DB916959		gi|85205875|gb|DW607560.1|DW607560	3.00E-26	77.6	solute carrier family 7 member 9+at		AA_permease	0.71213
DB916961		GS020727	0.005	32.2				
DB916997		gi|85194217|gb|DW603108.1|DW603108	1.00E-11	49.6	---NA---			0.8545
DB917015		gi|10202485|gb|BE781287.1|BE781287	0	308	glycyl-trna synthetase	P:GO:0006426,P:GO:0045055,F:GO:0005524,C:GO:0030141,F:GO:0004820	HGTP_anticodon,Pfam-B_5084,Pfam-B_8963	1.65772
DB917032	Embryo specific	gi|37599680|gb|CF725512.1|CF725512	0	236	cullin-associated and neddylation-dissociated 1	C:GO:0005575		0.5603

DB917065		gi|11974693|gb|BF689285.1|BF689285	2.00E-27	123	poly a bindingcytoplasmic 1 b	F:GO:0005488	PABP	1.63415
DB917078		BY795896	2.00E-25	111	arystal structure of the habc domain of neuronal syntaxin from the squid loligo pealei	C:GO:0016020,F:GO:0005484,P:GO:0016192,P:GO:0006886	Syntaxin	0.92096
DB917115		gi|46463206|gb|CN457480.1|CN457480	0	522	ribonucleotide reductase m1	C:GO:0005971,P:GO:0009263,P:GO:0006260,F:GO:0005515,P:GO:0051259,F:GO:0017076,F:GO:0004748	Ribonuc_red_lgC	
DB917131		gi|62021326|gb|DN679488.1|DN679488	7.00E-32	86.8	acetyl-coenzyme a carboxylase beta		CPSase_L_D2,CPSase_L_chain	0.65265
DB917133		gi|119782557|gb|EH279172.1|EH279172	2.00E-12	53.8	selenoprotein w2a	F:GO:0008430	Rdx	
DB917141		BY795054	2.00E-14	74.8	af454399_1fasciclin-like protein		Fasciclin,Fasciclin	
DB917148		gi|50138719|gb|CO471579.1|CO471579	7.00E-15	81.7	glutaredoxin-mitochondrial precursor	F:GO:0009055,F:GO:0015035,C:GO:0005575	Glutaredoxin	
DB917152		gi|54449072|gb|CV560438.1|CV560438	0.003	46.1				
DB917159		gi|15713204|gb|BI736191.1|BI736191	0.0001	33.6	fras1 related extracellular matrix protein 1	F:GO:0005529,P:GO:0007275,C:GO:0005578,C:GO:0005576,C:GO:0005575,F:GO:0005515,P:GO:0007160,F:GO:0046872,F:GO:0005509,P:GO:0007155,F:GO:0005488	Lectin_C	0.7063

DB917161		gi|18987264|gb|BM677368.1|BM677368	5.00E-05	49.2				
DB917181		gi|15708863|gb|BI731850.1|BI731850	0	436	heat shock protein 90		HSP90	1.35662
DB917183		gi|23657488|gb|BU732018.1|BU732018	0.0001	47.8				
DB917231		gi|62880192|gb|DN895429.1|DN895429	9.00E-15	81.3	nuclear receptor subfamilygroupmember 5	F:GO:0043565,F:GO:0003700,C:GO:0005634,F:GO:0004879,F:GO:0008270,P:GO:0006829,P:GO:0006355,F:GO:0004872,P:GO:0006353,F:GO:0046872,P:GO:0031564,P:GO:0006350,P:GO:0007601,F:GO:0003707	Hormone_recep	
DB917250		gi|15715211|gb|BI738198.1|BI738198	1.00E-06	41	kinesin-like protein unc-104 (protein immaculate connections)			
DB917271		gi|32181141|gb|CD679410.1|CD679410	0.0004	35.9	---NA---			
DB917289		BY799577	0.004	40.1				
DB917384		gi|37604110|gb|CF729942.1|CF729942	7.00E-10	65.1	still life type 1			0.77972
DB917386		BY795966	0.003	40.1				
DB917419		gi|32179217|gb|CD677486.1|CD677486	3.00E-29	117	nudix hydrolase 6			
DB917433		BY796191	3.00E-06	33.1				

DB917443		gi|22681209|gb| BU167225.1| BU167225	0.003	46.1			Pfam-B_16856	
DB917452		gi|24731857|gb| CA395939.1| CA395939	3.00E-07	56.5	---NA---		Pkinase	1.93198
DB917494		gi|22803240|gb| BU501159.1| BU501159	2.00E-32	108	elongation factor ts		EF_TS,EF_TS	0.72332
DB917518		BY795051	0	242	ribosomal protein l15	C:GO:0005840,F:GO:<br>0003735,P:GO:0006412	Ribosomal_L1<br>5e	1.73916
DB917523		BY795432	0.009	31.3				
DB917532		gi|15707842|gb| BI730829.1|BI730829	3.00E-40	163	protein<br>tyrosinereceptorm	F:GO:0016791,F:GO:<br>0004872,P:GO:0006470	Y_phosphatas<br>e	
DB917535		gi|19017737|gb| BM704479.1| BM704479	5.04E-44	143	basket cg5680-partial	F:GO:0004707,P:GO:<br>0006468,F:GO:0005524	Pkinase	1.8378
DB917590		BY796013	0.006	36.8				
DB917593		gi|85208787|gb| DW608631.1| DW608631	0.006	41.4			SH3_1	
DB917622		gi|46462290|gb| CN456564.1| CN456564	0.009	44.1	---NA---			
DB917660		GS004604	0.006	34.2			UIM	
DB917670		GS013969	0.005	34.2				
DB917672	**Embryo specific**	gi|75844134|gb| DT874394.1|DT874394	1.00E-07	53.5	trafficking protein<br>particle complex<br>subunit 3			
DB917693		gi|119789608|gb| EH282167.1| EH282167	3.00E-26	119	---NA---			0.55793
DB917697	**Positive selectio**	gi|62830261|gb| DN860612.1| DN860612	5.04E-44	178	lethals1921 cg2245-<br>pa			2.54494
DB917703		BY799323	0.004	40.1				

DB917713		gi|10592574|gb|BE952277.1|BE952277	5.00E-40	165	atp-bindingsub-family fmember 2	F:GO:0005524,F:GO:0016887	Pfam-B_11162	
DB917724		GS019928	0.002	36.2				
DB917726	Positive selectior	gi|85203147|gb|DW606219.1|DW606219	0	357	homolog 1	P:GO:0008150,C:GO:0005575	FtsJ,Pfam-B_40	5.43052
DB917728		gi|46815915|gb|CN503291.1|CN503291	1.00E-05	44	pleiotrophin(heparin-binding growth-associated molecule) (hb-gam) (heparin-binding growth factor 8) (hbgf-8) (osteoblast-specific factor 1) (osf-1) (heparin-binding neutrophic factor) (heparin-binding brain mitogen)		PTN_MK_C,PTN_MK_C,PTN_MK_C	0.91517
DB917766	Embryo specific	gi|37601458|gb|CF727290.1|CF727290	1.00E-12	48.3	lunapark b isoform 1		7tm_6,Pfam-B_7282,Pfam-B_8566	
DB917773		gi|85183291|gb|DW599817.1|DW599817	0	269	dna topoisomerase type i	F:GO:0003677,P:GO:0006259	Topoisom_I_N	0.786
DB917775		gi|22703862|gb|BU189878.1|BU189878	0.01	44.1				
DB917777		GS013936	9.00E-05	40.1				
DB917780		gi|22810959|gb|BU504726.1|BU504726	6.00E-37	99.6	death-associated protein 6	C:GO:0016605,F:GO:0005515,F:GO:0016564	Daxx	
DB917797		BY795427	1.00E-14	70.7	cofi_schpoame: full=cofiliname: full=actin-depolymerizing factor 1	C:GO:0044424	Cofilin_ADF,Pfam-B_8155	1.5354

DB917800		gi|49677444|gb|CO431150.1|CO431150	0	164	btb poz domain-containing protein 7		BACK	
DB917801		gi|62052542|gb|DN694838.1|DN694838	0.01	41.4			Pfam-B_13263	
DB917810		gi|12042305|gb|BF726394.1|BF726394	0.0007	48.1				
DB917850		GS020606	0.006	34.2				
DB917867		gi|57591534|gb|CX564505.1|CX564505	0	410	btb poz domain-containing protein kctd3			
DB917874		gi|85170412|gb|DW595532.1|DW595532	1.00E-16	87.7	leukotriene a-4 hydrolase	F:GO:0016787		
DB917876		gi|19019757|gb|BM706499.1|BM706499	6.00E-36	121	cp072_xenlaame: full=upf0472 protein c16orf72 homolog			1.40699
DB917901		GS014081	0.006	34.2				
DB917919		gi|49670130|gb|CO424003.1|CO424003	4.00E-23	109	sorting nexin 4	F:GO:0035091,P:GO:0007154,F:GO:0005515,P:GO:0015031	Vps5	
DB917932		gi|15709733|gb|BI732720.1|BI732720	0.0002	46.9	---NA---		Pfam-B_1587	
DB917946		GS020744	0.0003	38.2	---NA---			
DB917957		GS020498	0.005	34.2				
DB917972		GS004859	0.001	36.2				
DB917999		gi|62879485|gb|DN894722.1|DN894722	0	251	thioredoxin domain containing 4 (endoplasmic reticulum)			
DB918001		gi|85178073|gb|DW598188.1|DW598188	0	248	solute carrier familymember e2		TPT,TPT,EamA	0.5436
DB918016		gi|37597892|gb|CF723724.1|CF723724	0.0001	47.8	---NA---			

DB918019		gi|32173232|gb|CD671492.1|CD671492	7.00E-34	144	src family kinase	F:GO:0016301,F:GO:0005488	Pkinase_Tyr,SH2	0.6104
DB918022		BY800189	0.01	29.9				
DB918029		gi|22692856|gb|BU178872.1|BU178872	0	240	heat shock 70 kda protein		HSP70	0.03556
DB918034		gi|20364661|gb|BQ189110.1|BQ189110	0.0008	44.6	rna-binding protein			
DB918076	Embryo specific	GS014107	0.008	32.2				
DB918077		gi|55687958|gb|CV783018.1|CV783018	0	223	electrogenic nbc-like protein	C:GO:0016020,F:GO:0015380,P:GO:0006810	HCO3_cotransp,Pfam-B_1083	0.53722
DB918082		gi|23655294|gb|BU730919.1|BU730919	4.00E-09	50.1	thymidine phosphorylase		PYNP_C	
DB918083		gi|32180506|gb|CD678775.1|CD678775	0.003	46.1				
DB918107		gi|54450569|gb|CV561175.1|CV561175	0	258	atp gtp-binding protein	F:GO:0005525,P:GO:0006397,F:GO:0005524,C:GO:0005634	MobB,Clp1,Pfam-B_896	0.99895
DB918132		gi|10664305|gb|BE988186.1|BE988186	0.009	42.1				
DB918141		gi|62886101|gb|DN901338.1|DN901338	0.0008	44.1				
DB918147		gi|24720222|gb|CA389757.1|CA389757	0.003	46.1				
DB918152		gi|55688758|gb|CV783818.1|CV783818	0	114	neprilysin 4 cg4058-isoform a		Peptidase_M13	0.32307
DB918153		BY796415	0	188			TRAP_alpha	

DB918158		gi|58031981|gb|CX729524.1|CX729524	9.00E-10	64.9	novel proteinmurine pdz domain containing 3		PDZ	
DB918181		gi|62875748|gb|DN890985.1|DN890985	3.00E-38	120	mgc82665 protein	P:GO:0006506,F:GO:0017176,C:GO:0016021	Gpi1	
DB918182		BY800356	0.002	38.2				
DB918185		gi|58061096|gb|CX734260.1|CX734260	0	159	l-isoaspartyl protein carboxyllike	C:GO:0005737,F:GO:0008168	PCMT	0.87122
DB918190		gi|62879490|gb|DN894727.1|DN894727	1.00E-12	74.4	titin isoform n2-a		fn3 ,fn3,Pfam-B_5281,Pfam-B_2000	0.52188
DB918209		gi|55688982|gb|CV784042.1|CV784042	0.0008	45.1				
DB918210		GS014040	0.006	34.2				
DB918213		gi|16427361|gb|BB467415.2|BB467415	0.0002	50.1				
DB918219		gi|62044604|gb|DN690834.1|DN690834	0.01	44.1				
DB918241		GS004743	0.005	34.2				
DB918248		BY798038	1.00E-38	154	zinc finger protein 271 (zinc finger protein 7) (zinc finger protein znfphex133) (epstein-barr virus-induced zinc finger protein) (znf-eb) (ct-zfp48) (zinc finger protein dp) (znf-dp)			0.99741
DB918257		gi|37636248|gb|CF739910.1|CF739910	1.00E-19	48.3	hippocampus abundant transcript 1			

DB918271		gi|40319267|gb|CK353380.1|CK353380	0.0005	27.6				
DB918277		BY796836	0.006	35.9			fn3	
DB918284		gi|50139187|gb|CO471789.1|CO471789	0	258	transcription elongation regulator 1		FF,FF,FF	
DB918309		gi|45305225|gb|CK875594.1|CK875594	0.005	44.1	formate dehydrogenase alpha chain			
DB918338		GS020767	0.006	34.2				
DB918339		gi|62849576|gb|DN879621.1|DN879621	5.04E-44	178	glutamine synthetase	F:GO:0004356,P:GO:0006542	Gln-synt_C	1.71448
DB918345		gi|50136337|gb|CO470468.1|CO470468	1.00E-21	103	ribosomal protein l31	F:GO:0003735,P:GO:0006412,C:GO:0005840,C:GO:0005622	Ribosomal_L31e	1.42388
DB918350		GS020769	0.001	36.2				
DB918351		gi|46813993|gb|CN501369.1|CN501369	0.002	43.7				
DB918355		BY799695	0.003	37.7				
DB918369		gi|85203913|gb|DW606597.1|DW606597	0	394	dolichyl-diphosphooligosaccharide-protein glycosyltransferase	C:GO:0005789,F:GO:0005515,C:GO:0016021,F:GO:0004579,P:GO:0018279	DDOST_48kD	0.10024
DB918380	**Embryo specific**	gi|49670267|gb|CO424140.1|CO424140	0	296	tubulin tyrosine ligase-likemember 1		Pfam-B_195,TTL,Pfam-B_3648	
DB918384		GS014081	0.006	34.2				
DB918385		gi|62854718|gb|DN884763.1|DN884763	5.00E-17	88.1	cytochrome c oxidase subunit vic	C:GO:0016020	COX9,COX6C	

DB918388		gi|18511277|gb|BM462237.1|BM462237	0	171	secreted protein	F:GO:0005488	Pfam-B_7013,TPR_1,Pfam-B_3120,TPR_1,Pfam-B_2967,TPR_1,Pfam-B_7103	2.0678
DB918400		gi|49672982|gb|CO426688.1|CO426688	1.40E-45	182	myst histone acetyltransferase 2	F:GO:0003700,C:GO:0005634,F:GO:0008270,P:GO:0006355	MOZ_SAS	0.4232
DB918405		gi|75840928|gb|DT871188.1|DT871188	2.00E-05	50.1	---NA---		Pfam-B_423,zf-C2H2	
DB918409		BY797271	9.00E-21	95.9	formin binding protein 1	F:GO:0005515	SH3_1	
DB918410		gi|32180832|gb|CD679101.1|CD679101	0.0005	48.1				
DB918413		BY797408	0.009	35.9	armadillo protein	F:GO:0005488	Pfam-B_8871	
DB918414		GS020519	0.006	34.2				
DB918417		gi|24723747|gb|CA391663.1|CA391663	1.00E-09	64.8	ef hand calcium binding protein 1			
DB918441		gi|75847670|gb|DT877930.1|DT877930	0.008	37.7				
DB918451		GS020635	0.006	30.9				
DB918461		gi|85205029|gb|DW607145.1|DW607145	0	392	chaperonin isoform 1		Cpn60_TCP1	1.5818
DB918463		gi|49672068|gb|CO425774.1|CO425774	2.00E-26	119	glucose transporter	F:GO:0005215	Sugar_tr,Sugar_tr	0.7429
DB918464		gi|85184852|gb|DW600369.1|DW600369	0	427	fructose-bisphosphate aldolase	P:GO:0006096,F:GO:0004332	Glycolytic	
DB918479		BY796495	0.004	40.1				

DB918481		BY796140	2.00E-22	101	uncharacterized protein kiaa0406	Pfam-B_3076,Pfam-B_5532,Pfam-B_3077	
DB918504		BY795509	0.003	40.1		Pfam-B_10701,Pfam-B_6449,Pfam-B_6449	
DB918507		gi|85164073|gb|DW592560.1|DW592560	1.00E-13	77.1	elegans proteinpartially confirmed by transcript evidence	BACK	
DB918515		gi|9124116|gb|BE253696.1|BE253696	3.00E-23	92.6	novel proteinglutamate receptor interacting protein 1		
DB918517		GS020551	0.001	25.4			
DB918529		gi|37598652|gb|CF724484.1|CF724484	0	177	ring canal kelch protein	Kelch_1,Kelch_1,Kelch_1,Kelch_1,Kelch_1	
DB918534	Positive selectior	gi|58033858|gb|CX731384.1|CX731384	3.00E-31	136	regulator of g protein signaling	DEP,Pfam-B_771	2.50617
DB918535		gi|19030296|gb|BM717038.1|BM717038	2.00E-07	57	aminophospholipid transporter-classtypemember 2	Pfam-B_7818	
DB918540		gi|62020487|gb|DN679079.1|DN679079	2.00E-07	46.9	heme-binding protein	SOUL	
DB918553		gi|46821839|gb|CN509215.1|CN509215	6.00E-20	75.3	isoform c	Elf1	1.10808
DB918557		gi|62844013|gb|DN874072.1|DN874072	0.003	46.1			
DB918577		GS020592	0.006	34.2			

DB918581		gi|15708687|gb|BI731674.1|BI731674	9.00E-22	99.6	nadh dehydrogenase subunit 5	C:GO:0044464	NADH5_C,Oxidored_q1	0.8204
DB918585		gi|13059708|gb|BG296747.1|BG296747	0.003	46.1				
DB918587		gi|13057929|gb|BG295866.1|BG295866	5.00E-43	78.4	chromosome 9 open reading frame 78		Hep_59	0.37597
DB918596		gi|57592505|gb|CX565476.1|CX565476	0	248	pkm2a protein	F:GO:0030955,F:GO:0004743,F:GO:0000287	PK	3.1316
DB918612		BY797401	0.001	42.1				
DB918617		gi|40318338|gb|CK352451.1|CK352451	2.00E-12	73.9	zinc finger protein 533		zf-C2H2_jaz,zf-C2H2_jaz	
DB918626		gi|15706647|gb|BI729634.1|BI729634	6.00E-20	78.5	calponin-like protein	P:GO:0031032,F:GO:0003779	Calponin,Calponin,Calponin,Calponin,Calponin,Calponin	1.7782
DB918643		gi|10681185|gb|BE996337.1|BE996337	6.00E-08	58.8	---NA---			
DB918671		BY796301	0.004	38.2				
DB918674		gi|37598756|gb|CF724588.1|CF724588	0	352	septin 5a	C:GO:0031105,P:GO:0007049,F:GO:0005515,F:GO:0005525	Septin	0.3854
DB918677	Positive selectior	gi|37626470|gb|CF730140.1|CF730140	0	355	mitochondrial citrate synthase precursor	P:GO:0044262,C:GO:0005759,F:GO:0004108,P:GO:0006099	Citrate_synt	4.08072
DB918693		gi|62832182|gb|DN862533.1|DN862533	0	224	annexin a7		Annexin,Annexin	
DB918696		gi|23661204|gb|BU733868.1|BU733868	1.00E-11	71.2	microrchidia 2a	F:GO:0008270,C:GO:0005575	Pfam-B_12816	
DB918710		gi|18500023|gb|BM450983.1|BM450983	6.00E-33	141	ies6-similar protein		YL1_C	0.9255

DB918718		gi|54444263|gb|CV558070.1|CV558070	0	306	myeloid lymphoid or mixed-lineage leukemia		FYRC,SET	1.23405
DB918727		gi|16491547|gb|BB657721.1|BB657721	4.00E-19	95.9	riken cdna d230014k01		Glyco_hydro_85	
DB918730		GS008232	0.006	34.2				
DB918733		GS013972	0.006	34.2				
DB918739	Positive selectior	gi|18524537|gb|BM475495.1|BM475495	0	208	glia maturation factor gamma	F:GO:0005515	Cofilin_ADF	2.17042
DB918749		BY800356	0.007	36.3			Pfam-B_13263	
DB918788		GS013783	0.006	34.2				
DB918821		gi|54474538|gb|CV572201.1|CV572201	0.01	44.1				
DB918823		BY795432	0.006	36.8	---NA---			
DB918827		gi|85172829|gb|DW596553.1|DW596553	0.0004	46	lobe cg10109-pa		Pfam-B_13617	
DB918837		GS013989	0.002	36.2	---NA---			
DB918840		BY798672	0.007	32.2				
DB918850		gi|19354133|gb|BM904029.1|BM904029	0	297	leukotriene a4 hydrolase	F:GO:0005488,P:GO:0044237,P:GO:0044238,F:GO:0008237	Peptidase_M1	0.6401
DB918855		GS004626	3.00E-05	42.1				
DB918861		gi|49672294|gb|CO426000.1|CO426000	0.002	43.7	cg17233-isoform a isoform 2		Pfam-B_897,Pfam-B_897	
DB918862		gi|85204396|gb|DW606834.1|DW606834	1.00E-34	140	cg7154 cg7154-partial		DUF3512	
DB918888		gi|62048371|gb|DN692705.1|DN692705	2.00E-33	143	succinyl-ligase subunitmitochondrial precursor		Ligase_CoA	0.6879

DB918892		gi|14063502|gb|BG752849.1|BG752849	0	227	k6pf_schmaame: full=6-phosphofructokinase short=phosphofructokinaseame: full=phosphohexokinase	F:GO:0003824	PFK	0.81777
DB918895		gi|37635709|gb|CF739372.1|CF739372	5.00E-07	51.5	tpa: sco-spondin protein	F:GO:0005179,F:GO:0003674,F:GO:0005509,F:GO:0030414,P:GO:0007155,P:GO:0008150,C:GO:0005576,F:GO:0005515	TSP_1,Pacifastin_I,Cys_knot	
DB918908		gi|32178072|gb|CD676341.1|CD676341	0	215	eh-domain containing 3	F:GO:0003924,F:GO:0005509	efhand	
DB918911		GS013783	0.005	31.3				
DB918916		BY794962	6.00E-27	116	atp synthetase subunit 6	C:GO:0005743,C:GO:0045263,P:GO:0015992,F:GO:0015078,C:GO:0016021,F:GO:0016820	ATP-synt_A	

DB918920		gi|37598985|gb|CF724817.1|CF724817	2.00E-42	130	myosin vi	P:GO:0051046,C:GO:0031941,P:GO:0030048,C:GO:0031965,F:GO:0043531,P:GO:0050974,C:GO:0005794,P:GO:0006886,C:GO:0005905,C:GO:0001726,F:GO:0003774,P:GO:0006897,F:GO:0051015,P:GO:0030330,C:GO:0016591,P:GO:0045944,C:GO:0048471,F:GO:0005516,P:GO:0042491,C:GO:0005938,C:GO:0045334		
DB918922		gi|62045111|gb|DN691085.1|DN691085	3.00E-43	101	synaptophysin (major synaptic vesicle protein p38)	C:GO:0016021,C:GO:0030672,C:GO:0042734,F:GO:0015485,C:GO:0019717,P:GO:0006897	MARVEL	0.3569
DB918923		gi|62883019|gb|DN898256.1|DN898256	8.00E-39	107	sb:cb283 protein	P:GO:0006508,C:GO:0005622	Pfam-B_1295	0.39108
DB918928		gi|85176339|gb|DW597844.1|DW597844	0	256	elongation factor gmitochondrial	F:GO:0003746,C:GO:0005622,F:GO:0000166,P:GO:0006414,F:GO:0003924	EFG_IV,GTP_EFTU_D2	0.25377
DB918945		gi|49679974|gb|CO433680.1|CO433680	2.80E-45	182	cg17766-pa isoform 1		Pfam-B_9841	
DB918958		gi|75847792|gb|DT878052.1|DT878052	4.00E-19	60.2	---NA---		Pfam-B_5923	

DB918963		gi|11534013|gb|BF464830.1|BF464830	2.94E-44	179	cell death regulator aven		Pfam-B_5059,Pfam-B_18304	
DB918969		gi|15709785|gb|BI732772.1|BI732772	0.008	41.8	---NA---		Pfam-B_13263	
DB918998		gi|54447186|gb|CV559510.1|CV559510	0	383	uba and wwe domain containing 1	C:GO:0005737,P:GO:0006464,F:GO:0003677,P:GO:0051028,F:GO:0004842,C:GO:0005634	HECT	0.71877
DB919005		gi|4562379|gb|AI578003.1|AI578003	0.009	41.8				
DB919009		gi|39634652|gb|CK228548.1|CK228548	0.01	44.1				
DB919012		BY800189	0.001	33.1				
DB919022		gi|19391314|gb|BM932141.1|BM932141	0.002	46.1				
DB919025		gi|46855487|gb|CN527331.1|CN527331	0.002	46.1				
DB919029		gi|91744148|gb|EB380803.1|EB380803	0	196	ubiquitin-like modifier activating enzyme 1	F:GO:0008641,P:GO:0006512,F:GO:0005488	UBACT,UBA_e1_C,UBACT	0.42771
DB919034		gi|77505913|gb|DV216272.1|DV216272	8.00E-23	106	thyroid receptor interacting protein		Pfam-B_2751	
DB919048		gi|75848952|gb|DT879212.1|DT879212	2.00E-15	83.5	serine threonine-protein phosphatase 2a catalytic subunit alpha isoform			
DB919053		gi|57592476|gb|CX565447.1|CX565447	0.0006	48.1	nuclear protein hcc-1	P:GO:0008150,C:GO:0005575		
DB919086		gi|62037484|gb|DN687305.1|DN687305	0	211	---NA---		DUF1394	0.7005

DB919116		gi|85179409|gb|DW598458.1|DW598458	2.00E-07	56.5	5-methyltetrahydrofolate-homocysteine methyltransferase reductase		FAD_binding_1	
DB919122	**Embryo specific**	gi|9123195|gb|BE253045.1|BE253045	3.00E-19	67	glutaminyl-peptide cyclotransferase-like		Peptidase_M28	0.53552
DB919139		gi|54469703|gb|CV569820.1|CV569820	0.003	46.1	nuclear receptor subfamily 1 def			0.8208
DB919144		GS020600	0.002	36.2			Pfam-B_11119,Pfam-B_16930,Pfam-B_843,Rifin_STEVOR,Pfam-B_1037,Pfam-B_470,ASFV_J13L,DUF912	
DB919161		gi|62049971|gb|DN693543.1|DN693543	0.001	40				
DB919164	**Positive selectio**	gi|85209585|gb|DW608973.1|DW608973	0	412	signal recognition particle 54	F:GO:0005525,P:GO:0006614,F:GO:0017111,C:GO:0016020,C:GO:0048500,F:GO:0008312	SRP54_N,VirE,SRP54	2.44455
DB919194		gi|55687965|gb|CV783025.1|CV783025	8.00E-23	90	phosphodiesterase 11a	F:GO:0003824	GAF	0.77045
DB919217		gi|10650927|gb|BE981627.1|BE981627	0.009	44.1				
DB919218		gi|19039147|gb|BM720115.1|BM720115	2.00E-27	86.3	cytokine induced apoptosis inhibitor 1	F:GO:0003674,C:GO:0005575	CIAPIN1,CIAPIN1	0.39746

DB919224		gi|14062542|gb|BG751889.1|BG751889	1.00E-12	55.6	cg32486 cg32486-pd			
DB919232		gi|1483757|gb|AA019950.1|AA019950	2.00E-05	46	---NA---			
DB919247		gi|40321099|gb|CK355167.1|CK355167	0.0007	45.1				
DB919252		GS020699	0.007	30.2				
DB919264		GS004826	0.006	34.2				
DB919265		gi|58061010|gb|CX734174.1|CX734174	6.00E-07	46.4	---NA---		TUDOR	
DB919269		gi|24719522|gb|CA389401.1|CA389401	0.005	35.9			Pfam-B_6449,Pfam-B_4561,Pfam-B_6449	
DB919285	**GO annotation**	gi|57592033|gb|CX565004.1|CX565004	0	179	member ras oncogene family	P:GO:0015031,F:GO:0005525,P:GO:0007264	Ras	0.66686
DB919294		gi|21171127|gb|BQ432051.1|BQ432051	0	412	xpa binding protein 1	F:GO:0017111,C:GO:0005737,F:GO:0005524,F:GO:0005525	ATP_bind_1	0.6069
DB919298	**GO annotation, Positive selection**	gi|32178315|gb|CD676584.1|CD676584	1.00E-36	153	cornichon homolog 4	P:GO:0007242,C:GO:0016021,C:GO:0016020	Cornichon	2.32818
DB919300		GS004638	0.006	34.2				
DB919303		BY798085	0.007	36.3	---NA---		Pfam-B_8589,Pfam-B_8589	
DB919304		gi|16427590|gb|BB469110.2|BB469110	0.003	46.1				
DB919318		gi|62885542|gb|DN900779.1|DN900779	0.003	42.8	---NA---			

DB919327		gi|22684448|gb|BU170464.1|BU170464	0	331	kdel (lys-asp-glu-leu) endoplasmic reticulum protein retention receptor 2	F:GO:0004872,C:GO:0005789,C:GO:0005794,F:GO:0005515,F:GO:0005046,F:GO:0008565,P:GO:0016192,P:GO:0006886,C:GO:0016021,P:GO:0006621	ER_lumen_recept	1.8471
DB919337		gi|75842330|gb|DT872590.1|DT872590	2.00E-13	76.7	cg11699 cg11699-pa		Pfam-B_9283,DUF1358	
DB919351		gi|37638635|gb|CF742296.1|CF742296	0.01	32.7				
DB919363		gi|14974340|gb|BI298060.1|BI298060	0.0004	46.1				
DB919370		gi|58034682|gb|CX732208.1|CX732208	2.00E-17	90	glypican 5		Glypican	
DB919378	Embryo specific	gi|6515768|gb|AW209828.1|AW209828	0.002	40				
DB919382		gi|54443693|gb|CV557783.1|CV557783	1.00E-41	97.7	neprilysin 1 cg5905-isoform a isoform 1		Peptidase_M13_N	0.53162
DB919385		BY795432	3.00E-06	42.3	---NA---			
DB919388		gi|19048616|gb|BM727283.1|BM727283	1.00E-21	103	hermansky-pudlak syndrome protein			
DB919402		gi|85207147|gb|DW608127.1|DW608127	0.004	44.1				
	Positive selection	gi|19029446|gb|BM716188.1|BM716188	8.97E-44	87.7	cat eye syndrome chromosomecandidate 5	F:GO:0016787		2.00032
DB919423		gi|85196593|gb|DW603940.1|DW603940	2.00E-22	106	chromosome 20 open reading frame 116		DUF2042,DDRGK	0.92749

DB919428		gi|58034240|gb|CX731766.1|CX731766	1.00E-13	77	heterochromatin protein 1		Chromo	0.0465
DB919454		gi|62847739|gb|DN877784.1|DN877784	0	159	---NA---		Pfam-B_2982	0.1956
DB919457		gi|49672791|gb|CO426497.1|CO426497	0	253	calcium calmodulin-dependent serine protein kinase (maguk family)	F:GO:0004672,F:GO:0005488	Guanylate_kin	0.6048
DB919463		GS004835	0.005	34.2				
DB919489		BY797860	0.004	40.1				
DB919490		gi|62881650|gb|DN896887.1|DN896887	0	270	60s ribosomal protein l13a	F:GO:0003735,C:GO:0015934,P:GO:0006412	Ribosomal_L13	0.84639
DB919493		gi|46464405|gb|CN458679.1|CN458679	0	195	formylglycineamide ribotide amidotransferase	F:GO:0003824,C:GO:0009536		
DB919497		GS020551	0.005	30.9				
DB919499		gi|85165100|gb|DW593084.1|DW593084	1.00E-05	51	cad96ca cg10244-pa	F:GO:0016740,P:GO:0030324,C:GO:0016021,C:GO:0016020,P:GO:0006468,F:GO:0005524,F:GO:0004714,P:GO:0001759,F:GO:0004872	Pkinase_Tyr	
DB919502		GS014086	0.006	34.2				
DB919504	Positive selectior	gi|49672631|gb|CO426337.1|CO426337	0	240	elav 2-like protein	F:GO:0003676,P:GO:0048255	RRM_1,RRM_1	2.3784
DB919512		gi|54468701|gb|CV569322.1|CV569322	0	396	vacuolar protein sorting 26b-		Vps26	0.63451
DB919522		gi|62045742|gb|DN691402.1|DN691402	0	336	eukaryotic translation initiation factor 3 subunit	P:GO:0006413,F:GO:0003743	eIF-3_zeta	0.9211

DB919532		gi|62889265|gb|DN904502.1|DN904502	0.003	46.1				
DB919535		gi|58060842|gb|CX734006.1|CX734006	9.81E-45	161	sec24 related genemember a	P:GO:0006810,C:GO:0043231,C:GO:0044444	Sec23_helical,Sec23_BS	0.605
DB919573		gi|18502650|gb|BM453610.1|BM453610	2.00E-29	77.6	26s proteasome non-atpase regulatory subunit 3			1.47768
DB919626		gi|62025168|gb|DN681178.1|DN681178	0.002	46.1				
DB919632		GS020635	0.005	31.3				
DB919654		gi|9592429|gb|BB536929.1|BB536929	0.009	44.1				
DB919658		GS004874	0.006	34.2				
DB919673		gi|49680703|gb|CO434409.1|CO434409	2.00E-21	103	diras family protein		Ras	
DB919678		gi|85183348|gb|DW599836.1|DW599836	7.00E-08	58.5	hemagglutinin family protein		Apolipoprotein,Pfam-B_6360	
DB919682		GS004713	0.006	34.2			Pfam-B_13263,Pfam-B_13263,Pfam-B_4326,Pfam-B_4326	
DB919689		gi|22701075|gb|BU187091.1|BU187091	0	323	loc495086 protein	F:GO:0003824	Ligase_CoA	0.0164
DB919694		gi|62029256|gb|DN683163.1|DN683163	0	209	flavoprotein subunit of complex ii isoformpartial	F:GO:0050660,F:GO:0016491,P:GO:0006099	Succ_DH_flav_C,FAD_binding_2	0.56633

DB919698		gi|46818729|gb|CN506105.1|CN506105	0	418	cytochrome c-1	F:GO:0005488	Cytochrom_C1	2.02743
DB919705		gi|62025064|gb|DN681122.1|DN681122	1.00E-14	55.1	pancreatic lipase-related protein		Lipase	
DB919706		gi|75852061|gb|DT882321.1|DT882321	0.009	44.1				
DB919709		GS020584	0.001	36.2				
DB919714		gi|42746528|gb|CK780850.1|CK780850	2.00E-36	152	barrier-to-autointegration factor		BAF	1.00035
DB919719		gi|22707068|gb|BU193084.1|BU193084	3.00E-34	76.7	est1p-like protein b		Tektin	
DB919746		gi|20158553|gb|BQ108899.1|BQ108899	8.00E-15	76.2	---NA---	F:GO:0015171,C:GO:0016020,P:GO:0006810	AA_permease, UNC-50	
DB919748		gi|13057197|gb|BG295500.1|BG295500	0.006	44.1				
DB919755		GS004597	0.0008	34.2				
DB919758		gi|11531219|gb|BF462036.1|BF462036	0	184	replication factor c (activator 1) 3	F:GO:0005524,F:GO:0017111	RFC-E_C	0.64225
DB919760		gi|22361768|gb|BQ946290.1|BQ946290	0	357	developmentally regulated gtp binding protein 2	C:GO:0005622,F:GO:0005525	MMR_HSR1	1.42339
DB919767		gi|19045615|gb|BM724284.1|BM724284	2.00E-41	154	rcor3 protein		Myb_DNA-binding	1.2038
DB919770		GS013833	0.008	30.2				
DB919776		gi|62831095|gb|DN861446.1|DN861446	0.008	40.9				
DB919784		gi|32464087|gb|CD805261.1|CD805261	0	238	subfamilymember 13	F:GO:0003674,P:GO:0008150	Pfam-B_618	

DB919786		gi|57592538|gb|CX565509.1|CX565509	3.00E-28	124	mediator complex subunit 4 cg8609-pa		Med4	0.7867
DB919787		GS014073	0.006	30.2				
DB919798		GS013784	0.005	34.2				
DB919821		GS014124	0.008	30.4				
DB919823		gi|58032180|gb|CX729723.1|CX729723	0	321	escrt-ii complexhomolog	C:GO:0005737,P:GO:0006357,C:GO:0005667,F:GO:0008134,P:GO:0015031	EAP30	
DB919835		gi|22707271|gb|BU193287.1|BU193287	0	347	---NA---	C:GO:0045261,F:GO:0046933,P:GO:0015986,F:GO:0046872,F:GO:0005524,F:GO:0046961	ATP-synt_ab	
DB919848		gi|40321099|gb|CK355167.1|CK355167	0.003	42.8	---NA---			
DB919851		gi|62831095|gb|DN861446.1|DN861446	0.001	44.6				
DB919856		gi|75851478|gb|DT881738.1|DT881738	2.00E-10	65.9				
DB919889		gi|14061048|gb|BG750395.1|BG750395	2.00E-11	70.3	kiaa1813 protein isoform 2			
DB919898		BY795432	0.006	29.5				
DB919902		gi|42747349|gb|CK781671.1|CK781671	3.00E-18	92.7	---NA---		FERM_M	1.46845
DB919908		BY797096	0.0008	42.1				
DB919918		gi|46465385|gb|CN459659.1|CN459659	0.0003	46				
DB919919		gi|23662340|gb|BU734431.1|BU734431	0.002	46.1				

DB919927		gi|20337251|gb|BF455627.2|BF455627	0.01	44.1				
DB919932		BY795145	2.00E-27	72.5	60s ribosomal protein rpl22	F:GO:0003735,P:GO:0006412,C:GO:0005840,C:GO:0005622	Ribosomal_L22e	
DB919941		gi|12771566|gb|BG261750.1|BG261750	0.005	42.3				
DB919943		gi|85207821|gb|DW608344.1|DW608344	4.00E-07	56.1	co-chaperone-curved dna binding protein a	F:GO:0051082,P:GO:0006457,F:GO:0031072	DnaJ	
DB919958		gi|32177529|gb|CD675798.1|CD675798	9.00E-31	133	iron-sulfur assemblymitochondrial precursor		Fe-S_biosyn	1.07501
DB919968		gi|22376010|gb|BQ960532.1|BQ960532	0	472	mgc84682 protein	F:GO:0046872,C:GO:0005737,F:GO:0008237,C:GO:0008180	Mov34,Pfam-B_8170	2.06522
DB919992		gi|85170404|gb|DW595525.1|DW595525	0	214	propionyl coenzyme abeta polypeptide	F:GO:0016874	Carboxyl_trans	
DB920003		BY798745	0.0009	42.1	---NA---			
DB920004		BY795432	0.002	37.7				
DB920010		gi|4561003|gb|AI576627.1|AI576627	4.00E-10	65.2	cytochrome c oxidase copper chaperone		COX17	
DB920013		gi|46821571|gb|CN508947.1|CN508947	4.00E-19	89.3	immunoglobulin binding protein 1	P:GO:0009966,F:GO:0003674,C:GO:0005575	TAP42	
DB920032		GS004683	0.007	32.2				
DB920033		gi|85170536|gb|DW595579.1|DW595579	3.00E-08	59.3	skiv2l protein			
DB920054		BY799424	0.002	38.2				
