# Supplementary material for: Genetic mechanisms involved in the evolution of the cephalopod camera eye revealed by transcriptomic and developmental studies
Source: BMC Evol Biol. 2011 Jun 24;11:180. doi: 10.1186/1471-2148-11-180 (PMC3141435; doi:10.1186/1471-2148-11-180)

Figure S1. Phylogenetic tree based on the Ras domain sequences of centaurin superfamily members of squid, human and fly. Ras domain sequences of squid centaurin gamma homolog was aligned with those of representative centaurin gamma members and analyzed by the neighbor-joining method using Clustal W. The accession number corresponding to each member is centaurin delta1_human AAL04166, centaurin gamma1_human AF413077, centaurin gamma 1A_*Drosophila* NP_523562, RE07016p AAO39542, centaurin gamma2_human AF413078, and centaurin gamma3_human AF413079. Numbers at nodes indicate bootstrap values obtained 1,000 repetition. Bar indicates kimura distance calculated by Clustal W.

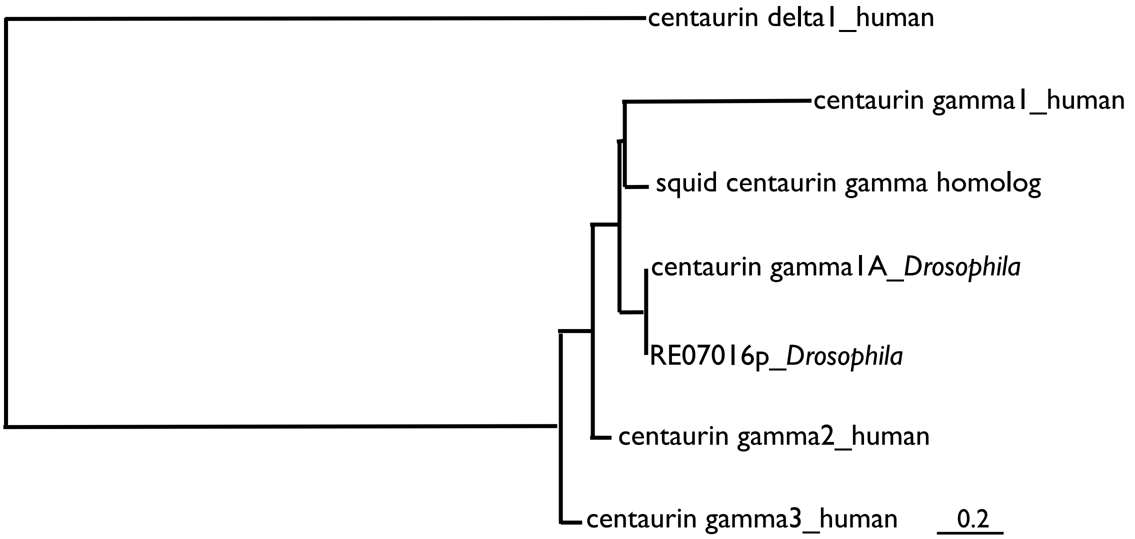

Supplement: Additional file 2 — FigureS1. Phylogenetic tree based on the Ras domain sequences of centaurin superfamily members. [file 1471-2148-11-180-S2.PDF]
