# Supplementary material for: Genetic mechanisms involved in the evolution of the cephalopod camera eye revealed by transcriptomic and developmental studies
Source: BMC Evol Biol. 2011 Jun 24;11:180. doi: 10.1186/1471-2148-11-180 (PMC3141435; doi:10.1186/1471-2148-11-180)

Figure S2. Distribution in camera eye-specific genes of GO terms at *level 2*. (A) biological processes, (B) molecular function, and (C) cellular component. GO terms for categorizing camera eye-specific genes were obtained using a blast2GO program.

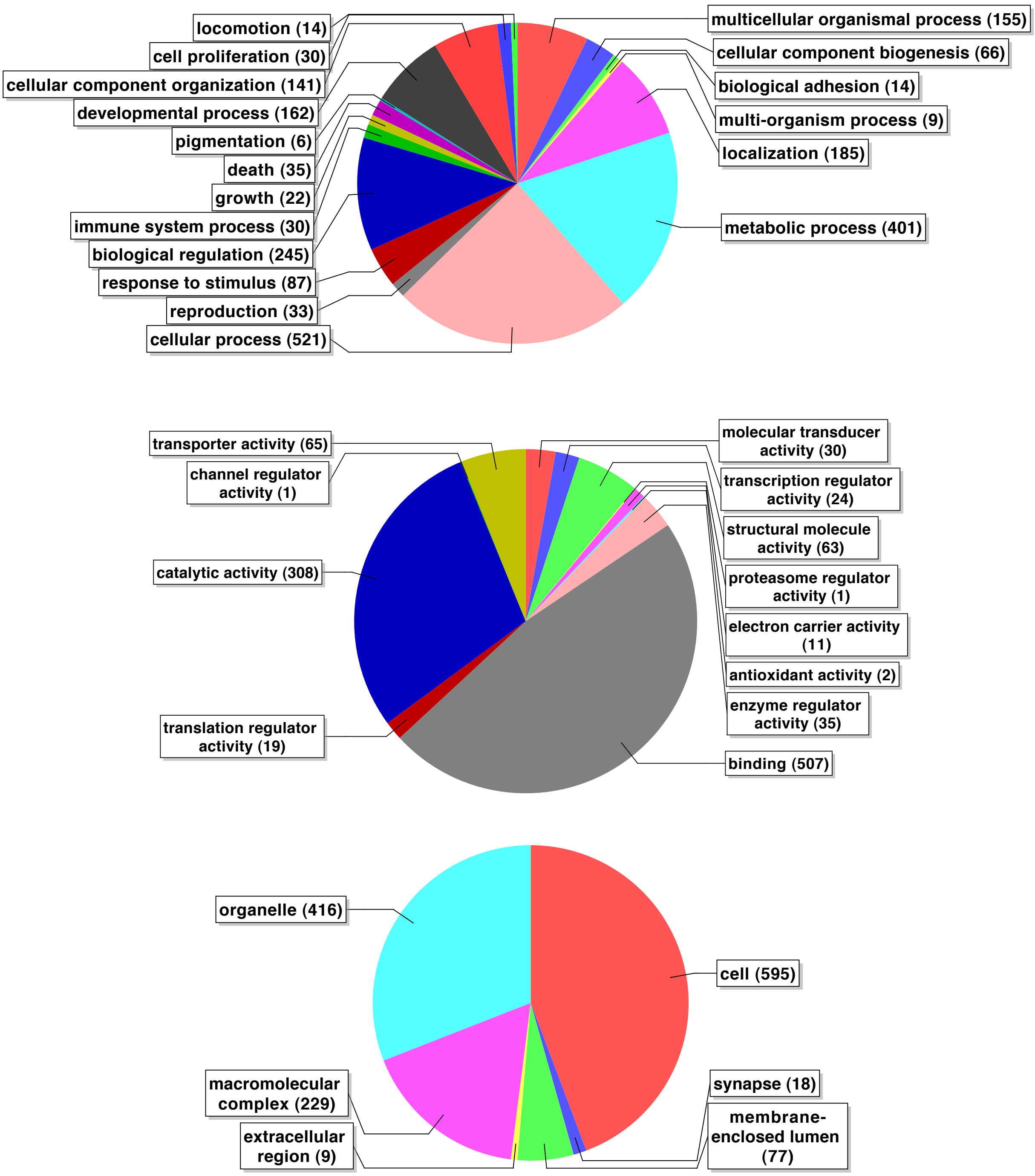

Supplement: Additional file 4 — FigureS2. Distribution in camera eye-specific genes of GO terms at level 2. [file 1471-2148-11-180-S4.PDF]
