# Supplementary material for: Genetic mechanisms involved in the evolution of the cephalopod camera eye revealed by transcriptomic and developmental studies
Source: BMC Evol Biol. 2011 Jun 24;11:180. doi: 10.1186/1471-2148-11-180 (PMC3141435; doi:10.1186/1471-2148-11-180)
Supplement: Additional file 5 — FigureS3. Phylogenetic tree based on Ets domain sequences of Ets transcription factor superfamily members. [file 1471-2148-11-180-S5.PDF]

Figure S3. Phylogenetic tree based on Ets domain sequences of Ets transcription factor superfamily members. ETS domain sequences of squid Ets-4 homolog was aligned with those of representative ETS members and analyzed by the neighbor-joining method using Clustal W. Spi1 were taken as out-group. The squid Ets-4 homolog is most closely related to *Drosophila* D-ets-3 (a homolog of human Fli-1). Close relationships among the squid Ets-4, D-ets-3, and human Fli-1/Erg is supported with high bootstrap value (91%). The accession number corresponding to each ETS member is Spi1_human P17947 as an outgroup, *C. elegans* protein C33A11.4 CAB01862.4, Elf3_human P78545, Etv6_human P41212, POK_*Drosophila* Q01842, Elf-1_human NP_758961, D-ets-4_*Drosophila* P29775, Etv5_human P41161, Elk-1_human P19419, Erg_human P11308, Erf_human P50548, GABPA_human Q06546, Ets-1_human P14921, ETS-family transcription factor_bivalve [*Chlamys farreri*] AAU11487, Ets at 98B_*Drosophila* AAF56746, D-ets-6/Ets at 21C_*Drosophila* AAF51484, D-ets-3/Ets at 65A_*Drosophila* AAF50697, D-ets-2/ *pointed*_*Drosophila* NP_524461, Fev_human NP_059991, and Fli-1_human AAH10115. Numbers at nodes indicate bootstrap values obtained 1,000 repetition. Bar indicates kimura distance calculated by Clustal W.

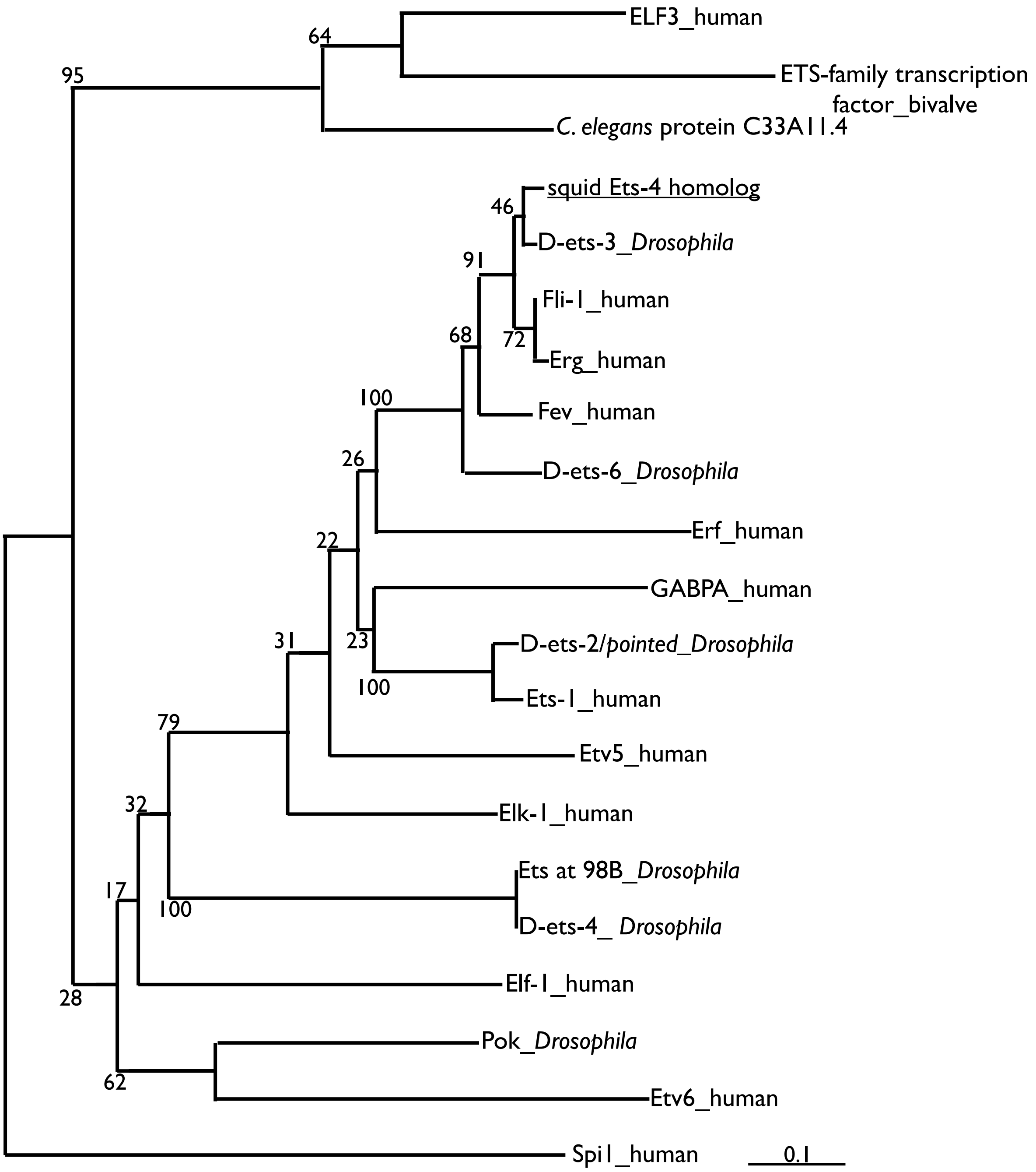
ELF3_human
ETS-family transcription factor_bivalve
C. elegans protein C33A11.4
squid Ets-4 homolog
D-ets-3_Drosophila
Fli-1_human
Erg_human
Fev_human
D-ets-6_Drosophila
Erf_human
GABPA_human
D-ets-2/pointed_Drosophila
Ets-1_human
Etv5_human
Elk-1_human
Ets at 98B_Drosophila
D-ets-4_ Drosophila
Elf-1_human
Pok_Drosophila
Etv6_human
Spi1_human
0.1
64
95
46
91
72
68
100
26
22
23
31
100
79
32
17
100
28
62
