# Supplementary material for: Genetic mechanisms involved in the evolution of the cephalopod camera eye revealed by transcriptomic and developmental studies
Source: BMC Evol Biol. 2011 Jun 24;11:180. doi: 10.1186/1471-2148-11-180 (PMC3141435; doi:10.1186/1471-2148-11-180)
Supplement: Additional file 7 — TableS2. Numbers of microarray probes showing positive signals to the molluscan RNAs. [file 1471-2148-11-180-S7.PDF]

Table S2. Positive signals obtained from *in vitro* homology search array

probe \ sample	Pygmy Squid stage 20 whole	Pygmy Squid stage 25 eye	Pygmy Squid stage 30 whole	Pygmy Squid adult eye	Pygmy Squid adult brain	nautilus adult eye	nautilus adult retina	scallop adult eye
squid	211 (3.8%)	253 (4.2%)	192 (3.3%)	256 (3.9%)	202 (3.3%)	189 (4.9%)	204 (4.8%)	432 (6.4%)
octopus	469 (8.3%)	600 (9.9%)	413 (7.1%)	612 (9.3%)	473 (7.8%)	521 (13.6%)	571 (13.3%)	1090 (16.1%)
scallop	124 (2.2%)	161 (2.7%)	152 (2.6%)	162 (2.5%)	128 (2.1%)	172 (4.5%)	339 (7.9%)	1322 (19.5%)
nautilus	110 (2.0%)	165 (2.7%)	98(1.7%)	173 (2.6%)	136 (2.2%)	1026 (26.8%)	1075 (25.0%)	398 (5.9%)
pygmy squid	3841 (68.4%)	3820 (63.2%)	4233 (72.4%)	4229 (64.4%)	4292 (70.9%)	630 (16.5%)	760 (17.7%)	1372 (20.2%)
Control	213 (3.8%)	219 (3.6%)	209 (3.6%)	240 (3.7%)	211 (3.5%)	263 (6.9%)	268 (6.2%)	289 (4.3%)
Total	4,968	5,218	5,297	5,672	5,442	2,801	3,217	4,903
