# Supplementary material for: Genetic mechanisms involved in the evolution of the cephalopod camera eye revealed by transcriptomic and developmental studies
Source: BMC Evol Biol. 2011 Jun 24;11:180. doi: 10.1186/1471-2148-11-180 (PMC3141435; doi:10.1186/1471-2148-11-180)
Supplement: Additional file 11 — TableS3. Primers to amplify homologs in the pygmy squid. [file 1471-2148-11-180-S11.PDF]

Table S3. List of primers to amplify homologs in the pygmy squid

Ets-4	F, TTGGATAGATGTTTCCGTTCGGCG
	R, AAGTTGTATCACCTGGGAGGGCCG
HMGb3	F, GGVATCCGTGGAGTGACGGAAGT
	R, GGTCCCGGCTCTGGCGGCCT
centaurin gamma	F1, AARYTGGGNATWYTNGGNAGTGT
	R1, GTDGCACANGTYTVRTARTANGWACA
	R2, ATMAYRCGNGGRTTRCTYTCACT
Hla-b associated	F1, GAYGTNCTNGARTTYAAYCARGT
	F2, AAGTCNGTNCCARCGNTGYATGGC
	R1, TCDATYTCRTCNGGNAGYTCTGT
